# Design and synthesis of novel thiazole/1,2,4-triazole/quinoline hybrids as antiproliferative agents, apoptosis inducers, immunomodulators, and multi-EGFR/BRAF^V600E^/HER-2 inhibitors

**DOI:** 10.1007/s11030-026-11467-9

**Published:** 2026-01-21

**Authors:** Aliaa M. Mohassab, Bahaa G. M. Youssif, Abdullah Yahya Abdullah Alzahrani, Hesham A. Abou-Zied, Stefan Bräse, Mohamed A. A. Abdel-Aal, Kamal S. Abdelrahman, Samar H. Abbas

**Affiliations:** 1 Medicinal Chemistry Department, Faculty of Pharmacy, Minia University, 61519 Minia, Egypt; 2 Medicinal Chemistry Department, Faculty of Pharmacy, Minia National University, New Minia, Egypt; 3 Pharmaceutical Organic Chemistry Department, Faculty of Pharmacy, Assiut University, 71526 Assiut, Egypt; 4 Department of Chemistry, Faculty of Science, King Khalid University, 61413 Abha, Saudi Arabia; 5 Medicinal Chemistry Department, Faculty of Pharmacy, Deraya University, Minia, Egypt; 6 Institute of Biological and Chemical Systems, IBCS-FMS, Karlsruhe Institute of Technology, 76131 Karlsruhe, Germany; 7 Department of Pharmaceutical Chemistry, Faculty of Pharmacy, Al-Azhar University, Assiut Branch, 71524 Assiut, Egypt

**Keywords:** Quinoline, 1,2,4-Triazole, Thiazole, Antiproliferative, Kinase inhibitors

## Abstract

We developed a novel category of quinoline-based compounds that operate as multi-target inhibitors of EGFR, HER-2, and BRAF^V600E^. The new compounds’ antiproliferative activity was tested against four cancer cell lines. All compounds had GI_50_ values ranging from 25 to 76 nM. Compounds **8f**, **8k**, and **8 m** exhibited the most potent antiproliferative action, with **8f** and **8k** surpassing erlotinib in all evaluated cancer cell lines. **8f** and **8k** are the most potent multi-target inhibitors of EGFR, HER-2, and BRAF^V600E^, surpassing the reference EGFR inhibitor, while failing to match the efficacy of Lapatinib and Vemurafenib. We evaluated the apoptotic potential of **8f** and **8k**, revealing that both compounds induce apoptosis by activating caspase-3, 8, 9, p53, and Bax, while downregulating the expression of Bcl-2, a protein that inhibits apoptosis. An integrated in silico workflow validated docking, 100-ns molecular dynamics (MD), and SwissADME were applied to compound **8f** against EGFR, BRAF^V600E^, and HER-2.

**Supplementary Information:**

The online version contains supplementary material available at 10.1007/s11030-026-11467-9.

## Introduction

Cancer is a predominant cause of mortality and a significant determinant in the enhancement of life expectancy. It is defined as a rebound mechanism of unregulated cellular proliferation [1]. In 2020, there were roughly 19.3 million new cancer cases and 10 million fatalities attributed to the disease globally [2, 3]. Although highly selective anticancer medicines demonstrate less unexpected toxicity, chemotherapeutic resistance frequently occurs with monotherapy [4–6]. To overcome this issue, clinical trials are using combination therapy of anticancer medicines with different modes of action. However, combined therapy has frequently resulted in undesirable side effects, including increased toxicity [7–9]. As a result, a single anticancer medication that inhibits many targets at the same time may have greater safety, fewer drug-drug interactions, a better pharmacokinetic profile, and higher patient satisfaction [10, 11].

Protein tyrosine kinases (PTKs) are vital in human cell signaling, controlling cellular proliferation, differentiation, angiogenesis, and a variety of regulatory activities [12, 13]. The overexpression of PTKs is essential in the genesis and progression of cancer due to their significant roles in cellular hemostasis. Moreover, PTK failure is fundamental to most cancer types, with PTKs representing over 60% of all oncoproteins and proto-oncoproteins, which are pivotal in cancer pathology [14, 15]. The epidermal growth factor receptor (EGFR or ErbB1) and human epidermal growth factor receptor 2 (Her2 or ErbB2) are two protein tyrosine kinases (PTKs) belonging to the Erb-B family, which are overexpressed in several solid tumors, including colon, breast, ovarian, lung, and prostate cancers. As a result, both receptors have been identified as effective targets for cancer therapy. Studies indicate that prolonged use of the EGFR inhibitor gefitinib can decrease EGFR expression and increase HER2 levels. Anti-EGFR therapy primarily inhibits EGFR signaling and has minimal impact on HER-2 signaling [16–18]. Thus, targeting both EGFR and HER-2 concurrently may effectively address the resistance observed with single-agent therapy [19, 20].

**Fig. 1:**
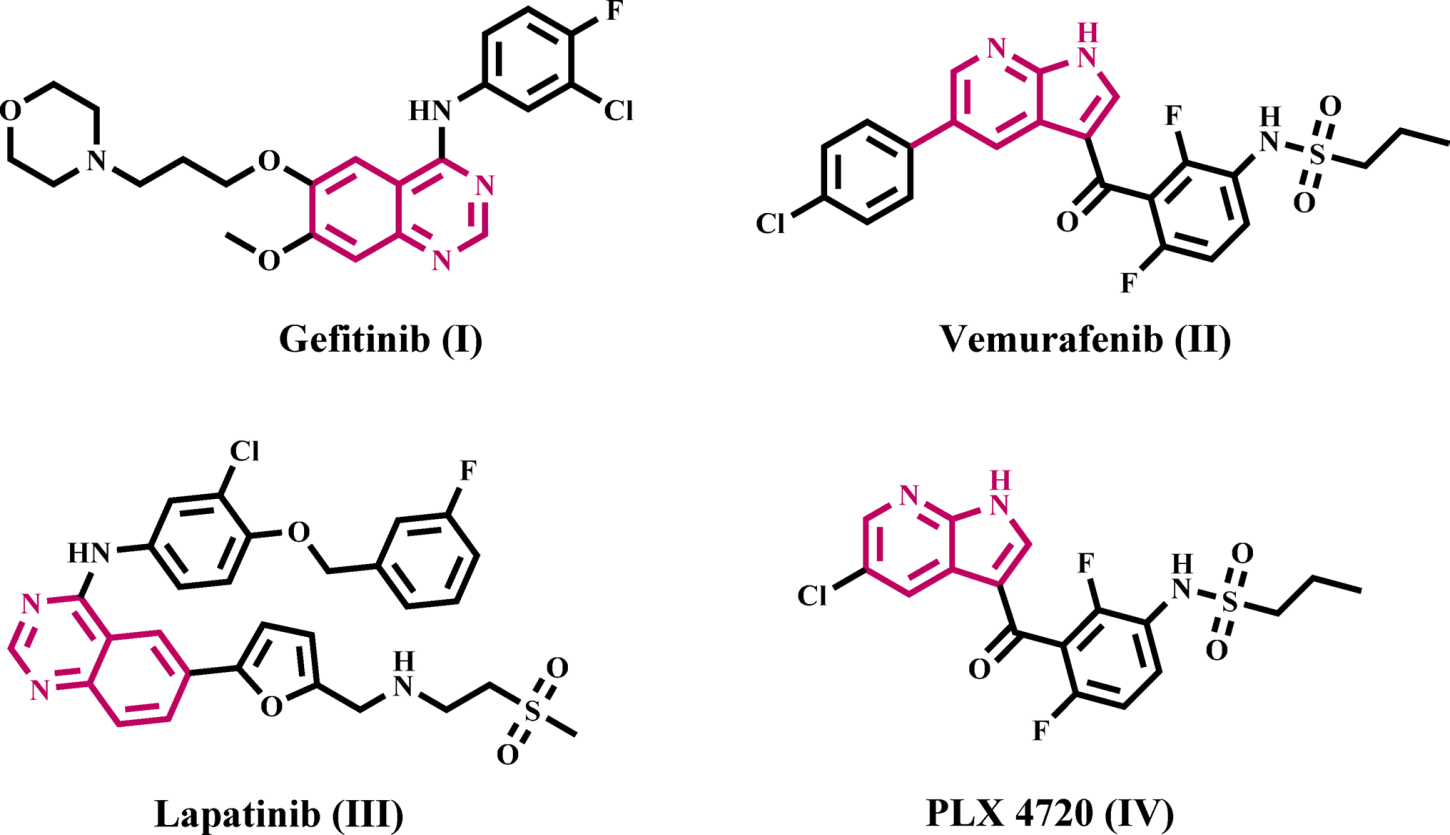
Structures of compounds **I-IV**

Unfortunately, EGFR mutations and compensatory mechanisms have significantly constrained the therapeutic effectiveness of EGFR monotherapy. The development of dual inhibitors targeting both EGFR and additional compensatory targets represents a potential strategy for addressing drug resistance in clinical contexts and warrants further investigation [21, 22]. For example, studies indicate that prolonged administration of the EGFR inhibitor gefitinib (compound **I**, Fig. 1) can diminish EGFR expression while elevating HER-2 levels. Nonetheless, anti-EGFR treatment solely obstructs EGFR signaling and exerts minimal influence on HER-2 signaling [23, 24]. Consequently, targeting both EGFR and HER-2 concurrently may serve as an efficacious strategy to overcome the resistance associated with monotherapy [25].

On the other hand, clinical investigations indicate that the combination of B-Rapidly Accelerated Fibrosarcoma (BRAF) and tyrosine kinase (TK) inhibitors successfully halts tumor proliferation and surmounts resistance [26, 27]. The simultaneous administration of EGFR inhibitors may reduce resistance to vemurafenib (compound **II**, Fig. 1), a mutant BRAF (BRAF^V600E^) inhibitor, in thyroid cancer. This combination has also shown promising results in BRAF^V600E^ colorectal cancer (CRC) [28, 29]. Additionally, the synergistic effects of the dual EGFR/HER-2 inhibitor lapatinib (compound **III**, Fig. 1) were comparable to those of the combination of PLX4720 (a BRAF^V600E^ inhibitor, compound **IV**, Fig. 1) with masitinib, a tyrosine kinase inhibitor (TKI), yet surpassed the effects of gefitinib, a selective EGFR inhibitor lacking activity against HER-2, thereby underscoring the beneficent role of HER family kinase inhibition [30]. Lapatinib was also effective in eliminating BRAF mutant CRC cells when used in combination with the protein kinase inhibitor MK2206, demonstrating notable synergistic effects [31]. Furthermore, lapatinib-induced HER inhibition made BRAF^V600E^ thyroid cancer cells more sensitive to BRAF^V600E^ inhibitor therapy and prevented the MAPK rebound effect in papillary thyroid carcinoma [31]. Further experiments revealed that lapatinib also increased radioiodine uptake, which is relevant given that BRAF^V600E^ mutant cells are resistant to radioiodine therapy [32]. In another study, Afatinib, an EGFR/HER-2 inhibitor, showed significant efficacy against COLO-205 CRC cells, characterized by elevated HER-2 expression, whereas its combination with vemurafenib exhibited synergistic effects on BRAF^V600E^ CRC [33].

Quinoline has been one of the most important scaffolds in drug development for the past few decades, especially in cancer research [34]. Quinoline, an N-based heterocyclic molecule, has numerous biological effects. Quinoline-containing compounds have dramatically increased basicity due to the presence of nitrogen atoms [35]. Many anticancer medicines that incorporate the quinoline molecule are now undergoing clinical testing [36]. Quinoline derivatives are effective in cancer treatment via a variety of mechanisms, including tyrosine kinase, EGFR, and mitogen-activated protein kinases [34]. Quinoline-derived anticancer medications include protein kinase inhibitors such as bosutinib, lenvatinib, and cabozantinib. Quinoline derivatives have shown promise in a variety of cancer cell lines, including those from the breast, colon, lung, colorectal, and kidney [37].

**Fig. 2:**
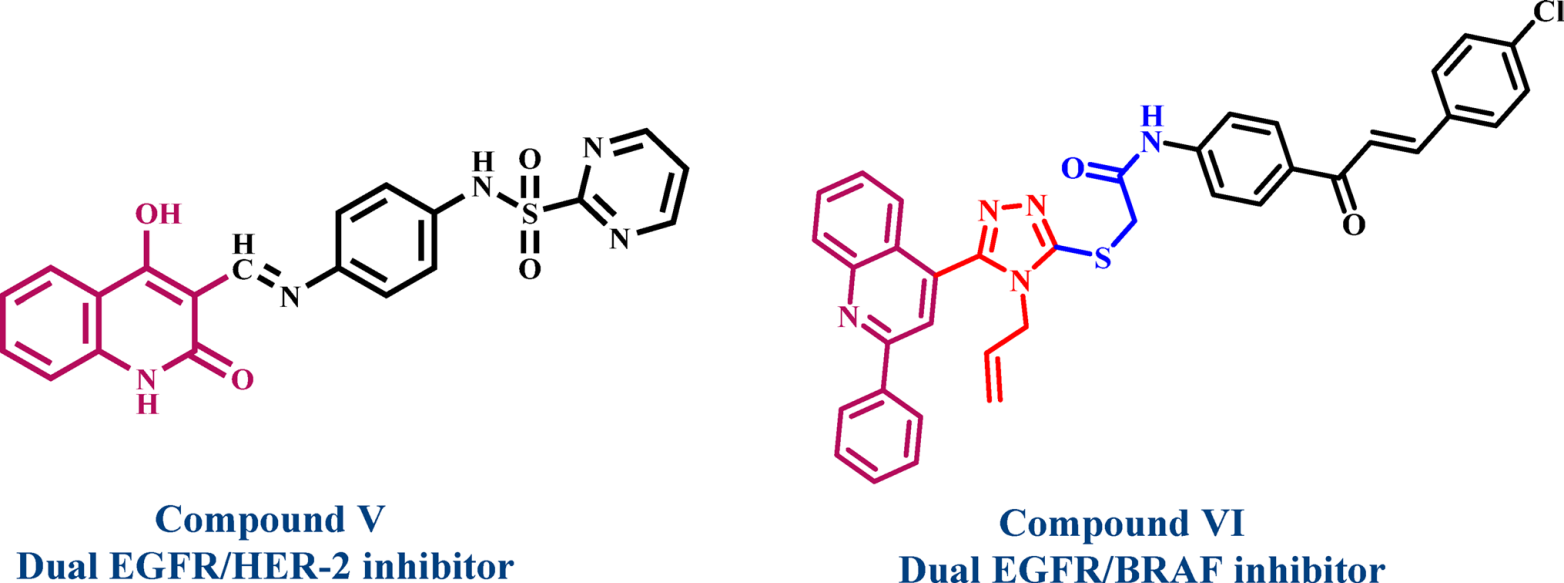
Structures of compounds **V** and **VI** as dual kinase inhibitors

In a recent work [38], we described a new class of EGFR/HER-2 dual-target inhibitors generated from quinoline moiety. The new compounds were tested for their antiproliferative activity against four cancer cell lines. All compounds had GI_50_ values ranging from 25 to 82 nM, with breast (MCF-7) and lung (A-549) cancer cell lines showing the highest sensitivity. Compound **V** (Fig. 2) had the most antiproliferative activity. Compound **V** outperformed the reference drug erlotinib (IC_50_ = 80 nM) as an EGFR inhibitor but fell short of the clinically used agent lapatinib (IC_50_ = 26 nM) as a HER-2 inhibitor, demonstrating the highest efficacy of dual-target inhibitors of EGFR and HER-2 with inhibitory (IC_50_) values of 71 and 31 nM, respectively. Furthermore, compound **I** reduces the expression of the anti-apoptotic protein Bcl-2 while increasing apoptosis by activating caspases 3, 8, and Bax.

In another work [39], we describe the synthesis of a new class of quinoline-based compounds that act as antiproliferative agents against EGFR and BRAF^V600E^. Compound **VI** (Fig. 2) demonstrated greater antiproliferative efficacy compared to doxorubicin (GI_50_ = 1.15 µM). It had a GI_50_ value of 3.30 µM against four human cancer cell lines. The compound effectively inhibited EGFR and BRAF^V600E^, with IC_50_ values of 1.30 ± 0.12 µM and 3.80 ± 0.15 µM, respectively. The reference erlotinib reported IC_50_ values of 0.08 ± 0.005 µM for EGFR and 0.06 ± 0.01 µM for BRAF^V600E^.

**Fig. 3:**
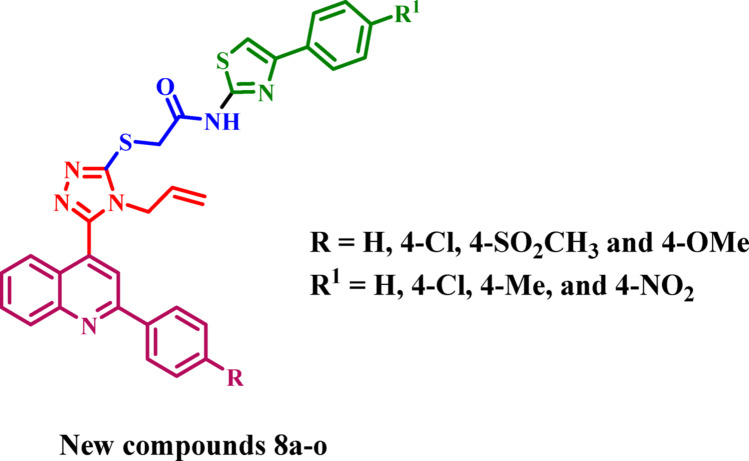
Structures of new compounds **8a-o**

This study expands on the advancement, synthesis, and biological evaluation of novel multi-kinase inhibitors targeting EGFR, HER-2, and BRAF^V600E^, building on previously published results as part of our ongoing initiative of developing dual- or multi-targeted antiproliferative medicines [9, 22, 38–46]. The new compounds **8a-o** are synthesized through the hybridization of the quinoline moiety, triazole, thiazole, and acetamido group (Fig. 3), aimed at enhancing their activity by facilitating hydrogen bonding through the acetamido group, thiazole, or triazole moiety. The hydrogen bonds, together with hydrophobic interactions, enhance binding and conformational stability at the receptor sites of molecular targets.

The newly synthesized compounds **8a–o** were tested for antiproliferative efficacy against a panel of four cancer cell lines. The most promising compounds were further tested as multi-EGFR/HER2/BRAF^V600E^ inhibitors. Additionally, we investigated the apoptotic and immunomodulatory potentials of the most effective derivatives. Finally, we performed molecular docking study to investigate the possible binding mechanisms and interactions between these compounds and receptor sites.

## Results and discussion

### Chemistry

**Scheme 1:**
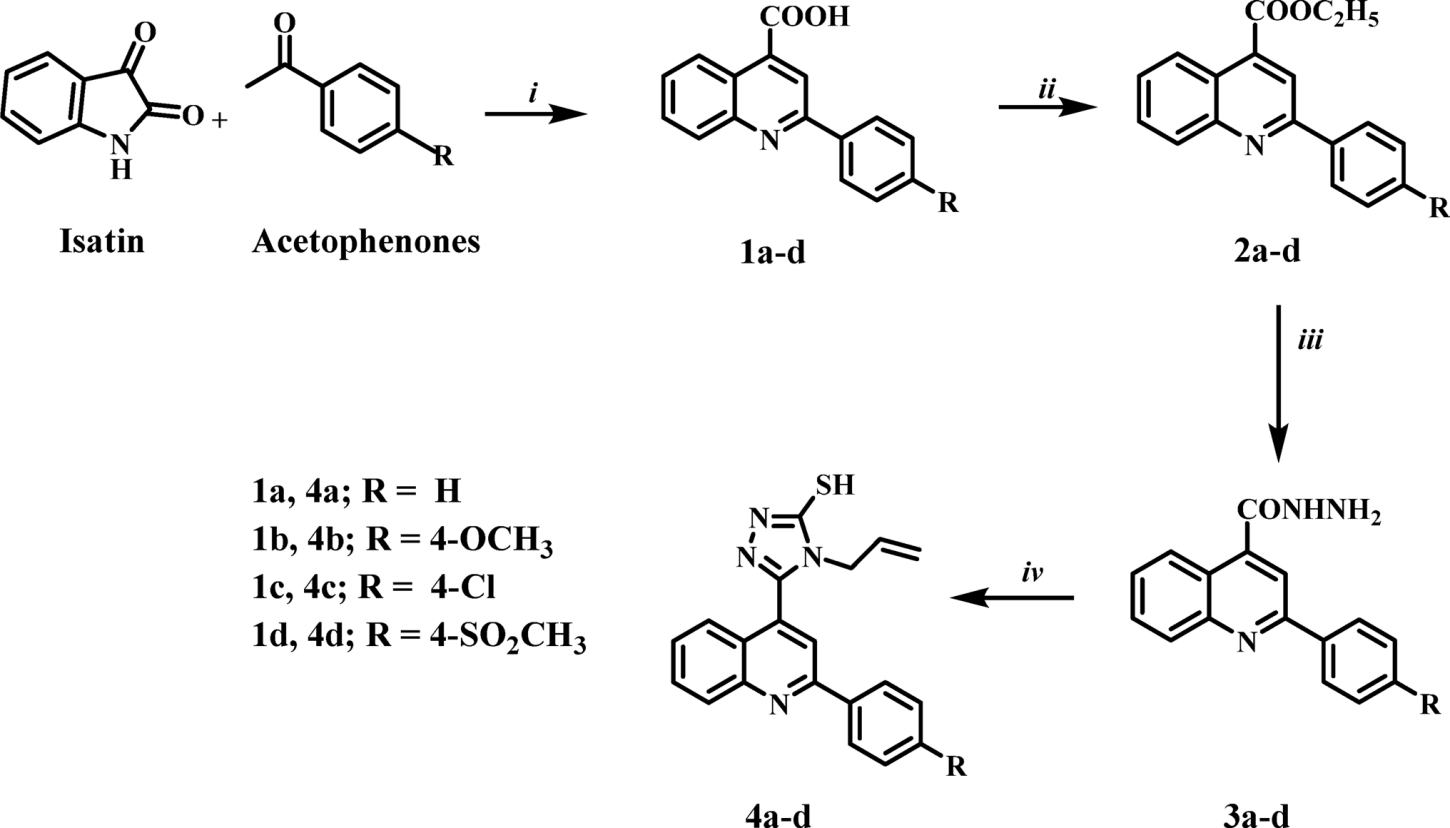
Synthesis of quinoline/1,2,4-triazole-3-thiol derivatives **4a-d**. Reagent and reaction conditions: **(i)** KOH, EtOH, reflux, 9–18 h; (75–93%) **(ii)** EtOH, Conc. H_2_SO_4_, reflux 10 h; (70–80%) **(iii)** NH_2_NH_2_, EtOH, reflux 3–7 h; (82–93%) **(iv)** 1-Allyl isothiocyanate, EtOH, reflux, 5 h, 2- NaOH, EtOH, reflux, 4 h then acidification with HCl, (68.0–84.0%)

**Scheme 2:**
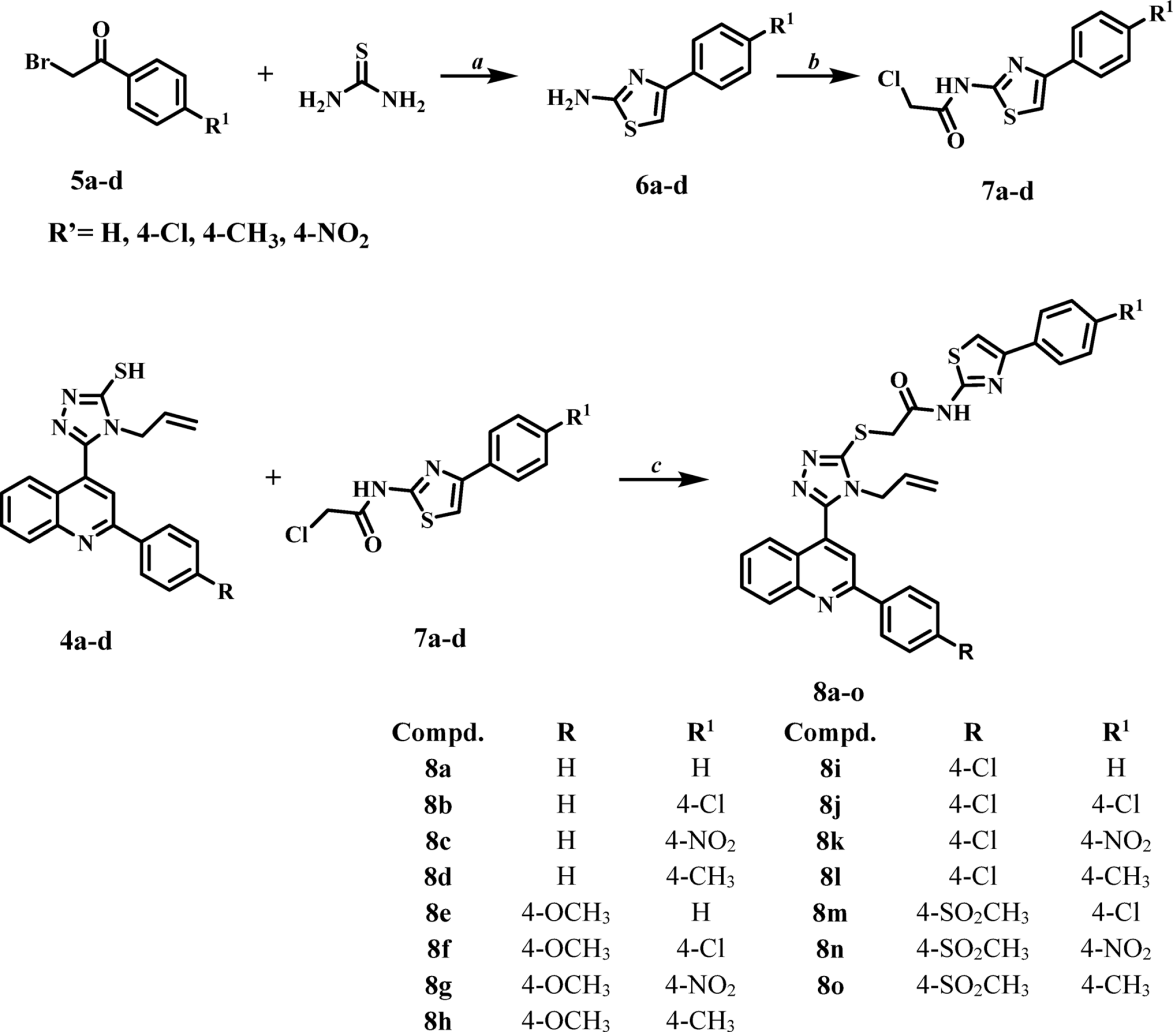
Synthesis of quinoline/triazole/thiazole derivatives **8a–o**. Reagent and reaction conditions: **(a)** EtOH, reflux, 1 h; (60–82%) **(b)** Chloroacetyl chloride, K_2_CO_3_ anhydrous, DCM, 2 h at 0 °C, rt, 24 h; (68–73%) **(c)** TEA, acetonitrile, rt, 4–8 h

 Schemes 1 and **2** explain the reaction sequences that were employed to synthesize target hybrids **8a-o** from the starting materials and intermediates (Supporting Information: Appendix A). Synthesis of triazoles **4a-d** as described in literature [39, 47] (Scheme 1). Initially, 2-(substituted-phenyl)quinoline-4-carboxylic acids **1a–d** were synthesized by refluxing isatin with the corresponding acetophenone in aqueous ethanol, utilizing potassium hydroxide as a catalyst. Subsequently, these acids **1a–d** were refluxed with absolute ethanol in the presence of concentrated H_2_SO_4_ as a dehydrating agent to yield the corresponding esters **2a–d**. Refluxing the ethyl esters **2a–d** with hydrazine monohydrate yielded carbohydrazide derivatives **3a–d**. The carbohydrazides **3a-d** were subjected to reflux with allyl isothiocyanate in ethanol. Aqueous 2 N NaOH is subsequently added, followed by acidification with concentrated HCl, yielding 1,2,4-triazole-3-thiol derivatives **4a–d** [48].

The thiazole amine intermediates **6a-d** were synthesized using the Hantzsch thiazole synthesis method [48, 49], involving the reaction of the corresponding phenacyl bromides **5a-d** with thiourea in heated ethanol, **Scheme 2**. The synthesized thiazole amine derivatives **6a-d** were exposed to an acylation process using chloroacetyl chloride in a biphasic solvent system consisting of water and DCM, with K_2_CO_3_ as the base [50]. The process yielded the corresponding acylated compounds **7a-d**. The validation of these compounds was accomplished by comparing their melting points with the documented data.

Target compounds **8a-o** (**Scheme 2**) were synthesized through the alkylation of the corresponding triazole derivatives **4a-d** with equimolar amounts of the appropriate acylated derivatives **7a-d** by stirring in acetonitrile at ambient temperature in the presence of triethyl amine (TEA) until precipitation ensued. The course of the reaction was monitored using thin-layer chromatography (TLC). The final **8a-o** were synthesized with yields between 60% and 86%.

The ^1^H NMR and ^13^C NMR as well as elemental analyses elucidated the new compounds **8a-o** structures (See supplementary File, **Figures ****S1****-S55**). The ^1^H NMR spectra of compounds **8a-o** showed singlet signals at δ 4.27–4.43 ppm related to the methylene protons of the linker (S-*CH*_*2*_-CO). Moreover, the allyl signals appeared as a doublet at δ: 4.47–4.67 ppm related to (N-C*H*_*2*_-CH = CH_2_), doublet of doublet at δ: 4.84–4.99 ppm and δ:5.11–5.26 ppm for (N-CH_2_-CH = C*H*_*2*_), with coupling constant *J* = 17.0–17.3 Hz and 10.1–11.5 Hz, respectively. As aforementioned, the appearance of allylic protons is due to the restricted rotation around the double bond. In addition to multiplet signals at δ: 5.55–6.04 ppm related to (N-CH_2_-*CH* = CH_2_). Furthermore, the amidic N*H* proton appeared as singlet signals at δ: 12.58–12.86 ppm. Additionally, their ^13^C NMR spectra revealed the presence of S*C*H_2_ signals, which appeared at δ: 36.05–36.82 ppm. Furthermore, the N*C*H_2_ signal appeared at δ: 47.21–47.80 ppm. All other carbons appeared at their expected chemical shifts. Elemental analyses proved the structure of the hybrids **8a-o**.

### Biology

#### Cell viability assay

To establish the safety of the newly synthesized compounds, the impact of new compounds **8a–o** on the viability of a normal human cell line, specifically human mammary gland epithelial cells (MCF-10 A), was investigated. The novel compounds were incubated with MCF-10 A cells for four days, and their cell viability was assessed using the MTT assay [51, 52]. Table 1 shows that at a concentration of 50 µM, all of the compounds under investigation maintained cell viability over 88%; none of them showed cytotoxicity towards normal cells.

#### Antiproliferative assay

Compounds **8a-o**’s antiproliferative properties on four human cancer cell lines: HT-29 (colon cancer), Panc-1 (pancreatic cancer), A-549 (lung cancer), and MCF-7 (breast cancer) were evaluated using the MTT test. Erlotinib was used as a reference [53, 54]. The average IC_50_ (GI_50_) and median inhibitory concentration (IC_50_) values for each substance assessed across the four cancer cell lines are shown in Table 1.

Generally, compounds **8a-o** exhibited significant antiproliferative activity, with GI_50_ values between 25 and 76 nM, compared to the reference erlotinib (GI_50_ = 33 nM). Moreover, in comparison to the other cell lines analyzed, all evaluated compounds exhibited a greater affinity for the lung (A-549) cancer cell line. Compounds **8c**, **8f**, **8k**, and **8 m** had the most significant antiproliferative activity, with GI_50_ values of 37, 25, 28, and 32 nM, respectively. Compounds **8c**, **8f**, **8k**, and **8 m** exhibited superior efficacy to erlotinib against the MCF-7 breast cancer cell line, with IC_50_ values between 25 and 37 nM, compared to erlotinib’s 40 nM. Furthermore, compounds **8f**, **8k**, and **8 m** outperformed erlotinib in terms of activity against the lung (A-549) cancer cell line, with IC_50_ values of 23, 25, and 27 nM, respectively, compared to erlotinib’s 30 nM.

Compound **8f** (*R* = 4-OMe, R^1^ = 4-Cl) outperformed all other compounds tested, with a GI_50_ of 25 nM, making it 1.3 times more potent than erlotinib (GI_50_ = 33 nM) against the four cancer cell lines examined. Compound **8f** demonstrated potent antiproliferative activity against the lung (A-549) and breast (MCF-7) cancer cell lines, with IC_50_ values of 23 and 25 nM, respectively, which are 1.3- and 1.6-folds as potent as erlotinib’s IC_50_ values of 30 and 40 nM against the same two cancer cell lines.

The substitution pattern at position four of the quinoline phenyl group (R) has a considerable impact on the antiproliferative activity of compounds **8a-o**, as does position four of the phenyl group of the thiazole moiety (R^1^). For example, compared to the *p*-methoxyphenyl derivative, **8f** (*R* = 4-OMe, R^1^ = 4-Cl), the compounds **8b** (R = H, R^1^ = 4-Cl), **8j** (*R* = 4-Cl, R^1^ = 4-Cl), and **8m** (*R* = 4-SO_2_CH_3_, R^1^ = 4-Cl), which possess an unsubstituted phenyl group, *p*-chlorophenyl, and *p*-methyl sulphonyl phenyl at position two of the quinoline moiety, exhibited reduced antiproliferative activity. Compounds **8b**, **8j**, and **8m** demonstrated GI_50_ values of 58, 42, and 32 nM, respectively, signifying a potency that is 2.3-, 1.7-, and 1.3-fold inferior to that of **8f**, thereby illustrating that the *p-*methoxyphenyl group at the second position of the quinoline moiety enhances antiproliferative activity, with activity increasing in the following order: *p-*methoxyphenyl > *p*-methyl sulphonyl phenyl > *p*-chlorophenyl > phenyl.

The antiproliferative action of compounds **8a-o** can be modified by the substitution pattern of the phenyl group at the fourth position of the thiazole moiety. For example, the unsubstituted derivative, compound **8e** (*R* = 4-OMe = R^1^ = H), the 4-nitro derivative, compound **8g** (*R* = 4-OMe = R^1^ = 4-NO_2_), and the 4-methyl derivative, compound **8h** (*R* = 4-OMe = R^1^ = 4-Me), all showed a marketed decrease in the antiproliferative activity when compared to the 4-chloro derivative, compound **8f** (*R* = 4-OMe = R^1^ = 4-Cl). Compounds **8e**, **8g**, and **8h** demonstrated IC_50_ values of 61, 76, and 72 nM, respectively, indicating they are 2.5-, 3-, and 2.9-fold less potent than compound **8f** (GI_50_ = 25 nM) against the four tested cancer cell lines. Compounds **8g** and **8h** were the least potent two derivatives among the newly synthesized compounds, showing that both nitro and methyl groups at the *para* position of the phenyl group in the thiazole moiety have reduced antiproliferative activity.

**Table 1:** Cell viability%, IC_50s_, and GI_50s_ values of compounds **8a-o** against four cancer cell lines


Comp.			Cell viability %	Antiproliferative activity IC_50_ ± SEM (nM)				
	R	R^1^		A-549	MCF-7	Panc-1	HT-29	Average (GI_50_)
**8a**	H	H	99	43 ± 3	48 ± 3	47 ± 3	49 ± 3	47
**8b**	H	4-Cl	88	55 ± 4	59 ± 4	60 ± 4	58 ± 4	58
**8c**	H	4-NO_2_	93	31 ± 2	37 ± 2	39± 2	40 ± 2	37
**8d**	H	4-Me	90	46 ± 3	51 ± 3	54 ± 3	52 ± 3	52
**8e**	4-OMe	H	92	57 ± 4	62 ± 5	64 ± 5	61 ± 5	61
**8f**	4-OMe	4-Cl	91	23 ± 1	25 ± 1	26 ± 1	25 ± 1	25
**8g**	4-OMe	4-NO_2_	88	71 ± 6	75 ± 6	77 ± 6	80 ± 6	76
**8h**	4-OMe	4-Me	90	67 ± 5	71 ± 5	75 ± 6	76 ± 6	72
**8i**	4-Cl	H	93	48 ± 3	54 ± 4	56 ± 4	54± 4	53
**8j**	4-Cl	4-Cl	91	37 ± 2	43 ± 3	45 ± 3	44 ± 3	42
**8k**	4-Cl	4-NO_2_	90	25 ± 1	28 ± 1	29 ± 1	30 ± 1	28
**8l**	4-Cl	4-Me	89	51 ± 4	56 ± 4	59 ± 4	58 ± 4	56
**8m**	4-SO_2_CH_3_	4-Cl	92	27 ± 1	32 ± 2	35 ± 2	34 ± 2	32
**8n**	4-SO_2_CH_3_	4-NO_2_	88	62 ± 5	68 ± 5	66 ± 5	64 ± 5	65
**8o**	4-SO_2_CH_3_	4-Me	89	39 ± 2	46 ± 3	45 ± 3	47 ± 3	44
**Erlotinib**	--	--	ND	30 ± 3	40 ± 3	30 ± 3	30 ± 3	33

#### EGFR inhibitory assay

The EGFR-TK assay was used to evaluate the efficacy of compounds **8f**, **8k**, and **8 m**, the most potent antiproliferative agents, to inhibit EGFR [55, 56]. Table 2 displays the results using erlotinib as the reference medication. The results of this assay are consistent with the findings of the antiproliferative assay. Compounds **8f**, **8k**, and **8 m** showed potent EGFR inhibitory activity, with IC_50_ values ranging from 67 to 76 nM. In all cases, the compounds tested outperformed erlotinib, which had an IC_50_ value of 80 nM. Compound **8f** (*R* = 4-OMe; R^1^ = 4-Cl), the most efficient antiproliferative derivative, had the highest EGFR inhibitory activity with an IC_50_ value of 67 nM, 1.2-fold more potent than the reference erlotinib. Compound **8k** (*R* = 4-Cl; R^1^ = 4-NO_2_) ranked second in EGFR inhibitory activity, with an IC_50_ value of 72 nM, exceeding the reference erlotinib (IC_50_ = 80 nM). Finally, compound **8 m** (*R* = 4-SO_2_CH_3_; R^1^ = 4-Cl) was the least efficient EGFR inhibitor, with an IC_50_ of 76 nM, but had comparable efficacy to erlotinib, Table 2. According to these results, compounds **8f**, **8k**, and **8 m** are potent antiproliferative agents that might also have EGFR inhibitory effects.

**Table 2:** IC_50_ values of **8f**, **8k**, and **8 m** against EGFR, BRAF^V600E^, and HER-2

Compound	EGFR inhibition IC_50_ ± SEM (nM)	BRAF^V600E^ inhibition IC_50_ ± SEM (nM)	HER-2 inhibition IC_50_ ± SEM (nM)
**8f**	67 ± 3	49	33
**8k**	72 ± 4	53	38
**8 m**	76 ± 4	58	41
**Erlotinib**	80 ± 5	--	--
**Vemurafenib**	--	30 ± 2	--
**Lapatinib**	--	--	26 ± 2

--, not determined

#### BRAF^V600E^ inhibitory assay

The most potent derivatives, **8f**, **8k**, and **8 m**, with interesting antiproliferative traits, were tested for their potential to inhibit mutant BRAF using vemurafenib as a reference [41, 42]. Table 2 presents the results as IC_50_ values. The three experiment means ± SD are used for all values. Compounds **8f**, **8k**, and **8 m** exhibited enhanced BRAF^V600E^ inhibitory action, with IC_50_ values of 49, 53, and 58 nM, respectively. In every instance, the compounds evaluated exhibited lower potency than the reference vemurafenib, which has an IC_50_ value of 30 nM. Once again, compound **8f**, the most effective antiproliferative and EGFR inhibitor, exhibited the highest potency as a BRAF^V600E^ inhibitor with an IC_50_ value of 49 nM, which is 1.6-fold less potent than vemurafenib. These findings showed that compounds **8f**, **8k**, and **8 m** can operate as antiproliferative agents targeting both EGFR and mutant BRAF. Still, they require structural adaptations to boost their efficacy against their molecular targets.

#### HER-2 inhibitory assay

The compounds **8f**, **8k**, and **8 m** were investigated for their propensity to inhibit HER-2 utilizing the kinase assay [38, 46]. The results are shown in Table 2. Lapatinib served as the reference control. The results indicated that the compounds tested substantially inhibited HER-2, with IC_50_ values ranging from 33 to 41 nM, compared to lapatinib’s IC_50_ of 26 nM. All of the compounds that were tested were less potent than the lapatinib reference drug. Compound **8f** was once again the most effective HER-2 inhibitor, with an IC_50_ value of 33 nM, which is 1.2 times less potent than lapatinib. The results indicated that compound **8f** is a promising antiproliferative candidate exhibiting multi-EGFR/BRAF^V600E^/HER-2 inhibitory action, necessitating structural modifications for lead optimization.

#### Apoptotic markers assays

One of the fundamental hallmarks of human cancer is apoptosis dysregulation, which causes uncontrolled cell division, a poor response to therapy, and the development of drug-resistant cells [48]. As a result, contemporary anticancer treatments have been shown to induce apoptosis in cancer cells via both intrinsic and extrinsic routes. Therefore, compounds **8f** and **8k** were tested for their potential to trigger apoptosis in A-549 lung cancer cells by measuring the expression of important apoptotic markers such as Bcl-2, p53, and Bax [57]. The results are displayed in Table 3.

Apoptosis is primarily regulated by the Bcl-2 protein family, which comprises both pro-apoptotic (Bax) and anti-apoptotic (Bcl-2) [58]. Several studies have found a substantial correlation between increased Bcl-2 levels and decreased Bax levels, both of which are connected to tumor cell proliferation [59]. The Bcl-2 and Bax protein levels in A-549 lung cancer cells treated with compounds **8 g** and **8 h** were thus determined. Table 3 demonstrates that compound **8f** elevated Bax levels by 8.70-fold and reduced Bcl-2 levels by 4.15-fold in comparison to control, untreated cells. Moreover, compound **8k** diminished Bcl-2 levels by 3.60-fold and augmented Bax levels by 8.25-fold. These data indicate that the antiproliferative effects of the investigated medicines may be influenced by apoptosis.

**Table 3:** Results of apoptotic assays for **8f** and **8k** against Bax, p53, and Bcl-2

Compound No.	Bcl-2 (ng/mL)	Fold reduction	Bax (pg/mL)	Fold change	p53 (pg/mL)	Fold change
8f	1.20 ± 0.001	4.15	520 ± 3	8.70	360 ± 2	5.50
8k	1.40 ± 0.001	3.60	495 ± 2	8.25	345 ± 2	5.30
Control	5	1	60	1	65	1

Cancer cells frequently inactivate p53 enzymes during transformation, which could be explained by p53 overexpression’s ability to trigger apoptosis [60]. Cancer cells treated with compounds **8f** and **8k** demonstrated a considerable increase in p53 levels, which were at least five times greater than those of untreated control cells. These data imply that the apoptotic process in these new compounds is mediated by increased p53 proteins.

Furthermore, caspase activity is required for both initiating and terminating the apoptotic process. Apoptosis is caused by caspase-3, a key enzyme that cleaves many proteins inside cells [61]. The effects of compounds **8f** and **8k** on caspase-3 were tested on the lung (A-549) cancer cell line and compared to staurosporine as a reference medicine, Table 4.

Compared to the reference staurosporine (465 ± 4 pg/mL), compound **8f** was the most effective derivative, with significantly higher caspase-3 protein levels (535 ± 5 pg/mL). Compound **8f** significantly increased caspase-3 levels, resulting in an 8.20-fold increase compared to control A-549 cells. Additionally, compound **8k** increased active caspase-3 levels by 7.80-fold (505 ± 5 pg/mL) compared to untreated lung cells (A-549).

**Table 4:** Caspases 3, 8, and 9 assays of compounds **8f** and **8k**

Compd. No.	Caspase-3		Caspase-8		Caspase-9	
	Conc (Pg/ml)	Fold change	Conc (ng/ml)	Fold change	Conc (ng/ml)	Fold change
8f	535 ± 5	8.20	2.05 ± 0.20	20.50	24 ± 3	24
8k	505 ± 5	7.80	1.95 ± 0.10	19.50	22 ± 2	22
Staurosporine	465 ± 4	7.00	1.85 ± 0.10	18.50	20 ± 1	20
Control	65	1.0	0.10	1	1	1

To determine whether compounds **8f** and **8k** cause apoptosis via the intrinsic or extrinsic pathway, their effects on caspase-8 and caspase-9 were investigated. The results show that compound **8f** increases caspase-8 and caspase-9 levels by 20.5 and 24 times, respectively, whereas compound **8k** increases caspase-8 and caspase-9 levels by 19.5 and 22 times, respectively, when compared to the control A-549 cancer cells. This suggests that both the intrinsic and extrinsic pathways are activated, with the intrinsic pathway having a greater impact, as evidenced by elevated caspase-9 levels (Table 4).

#### Effects on immunomodulatory proteins (TNF-α and IL-6)

Cytokines have an important role in cancer genesis and progression. Anti-cytokine drugs are being studied extensively, which could lead to new treatments for symptoms that are now difficult to manage [62, 63]. Interleukin-6 (**IL-6**) and tumor necrosis factor-alpha (**TNF-α**) are two cytokines that contribute to tumor growth and metastasis. TNF-α has been linked to cancer development and metastasis in both experimental and human models [64]. Pharmaceutical development benefits from developing anticancer medicines that inhibit both TNF-α and IL-6.

The qRT-PCR test was used to assess the impact of the most potent compounds, **8f** and **8k**, on immunomodulatory protein levels (TNF-α and IL-6) [65]. Compounds **8f** and **8k** were applied to A-549 cells for 24 h at doses of 25 nM and 28 nM (IC_50_ against A-549), respectively. Dexamethasone, a medication that consistently controls the immune system, served as the reference drug.

Compounds **8f** and **8k** significantly reduced TNF-α levels, with inhibition rates of about 80%, equivalent to Dexamethasone’s 83%. Compound **8f** suppressed IL-6 (89%), comparable to that of dexamethasone (93%), while compound **8k** suppressed IL-6 by 84%, Table 5. These findings revealed that both **8f** and **8k** may act as apoptotic antiproliferative agents with immunomodulatory properties.

**Table 5:** % Inhibition of compounds **8f** and **8k** against TNF-α and IL-6

Compound	TNF-α (% inhibition)	IL-6 (% inhibition)
**8f**	81	89
**8k**	77	84
**Dexamethasone**	83	93

#### In vitro cytotoxicity against normal human cell line

To determine the selectivity of the target compounds for cancer cells over normal cells, the safety profiles of the most effective compounds, **8f** and **8k**, were evaluated on the normal human diploid cell line (WI-38) using the MTT assay [51, 52]. Compounds **8f** and **8k** had IC_50_ values larger than 200 nM. Table 6 shows that the examined compounds had a favorable safety margin for normal cells.

**Table 6:** IC_50_ values of **8f** and **8k** against normal (WI-38) and cancer cell lines A-549 and MCF-7

Compound	Cytotoxicity (WI-38) IC_50_ (nM)	Selectivity Index (SI)	
		A-549	MCF-7
**8f**	> 200	> 8.7	> 8.0
**8k**	> 200	> 8.0	> 7.1

### Docking study into EGFR, BRAF^V600E^, and HER-2

To gain molecular-level insights into the binding behavior of the most potent derivative, compound **8f**, a comprehensive molecular docking analysis was performed against EGFR, BRAF^V600E^, and HER-2. This study aimed to correlate the experimentally observed multi-kinase inhibitory activity of **8f** with its structural pharmacophore features, namely, the quinoline core, 1,2,4-triazole linker, thioether-acetamide bridge, and the *para*-methoxyphenyl and *p*-chlorophenyl terminal motif.

Docking calculations were carried out using validated crystallographic structures from the Protein Data Bank: EGFR in complex with erlotinib (PDB ID: 1M17) [66], BRAF^V600E^ in complex with vemurafenib (PDB ID: 3OG7) [67], and HER-2 in complex with a known inhibitor (PDB ID: 3PP0) [25]. These high-resolution structures served as reliable templates for evaluating the ligand-binding orientations and intermolecular interactions of compound **8f**. Protein structures were preprocessed by removing crystallographic water molecules, protonating titratable residues, and assigning appropriate bond orders. Energy minimization was performed using the OPLS-AA force field to ensure optimal side chain conformations and preserve binding site geometry.

**Fig. 4:**
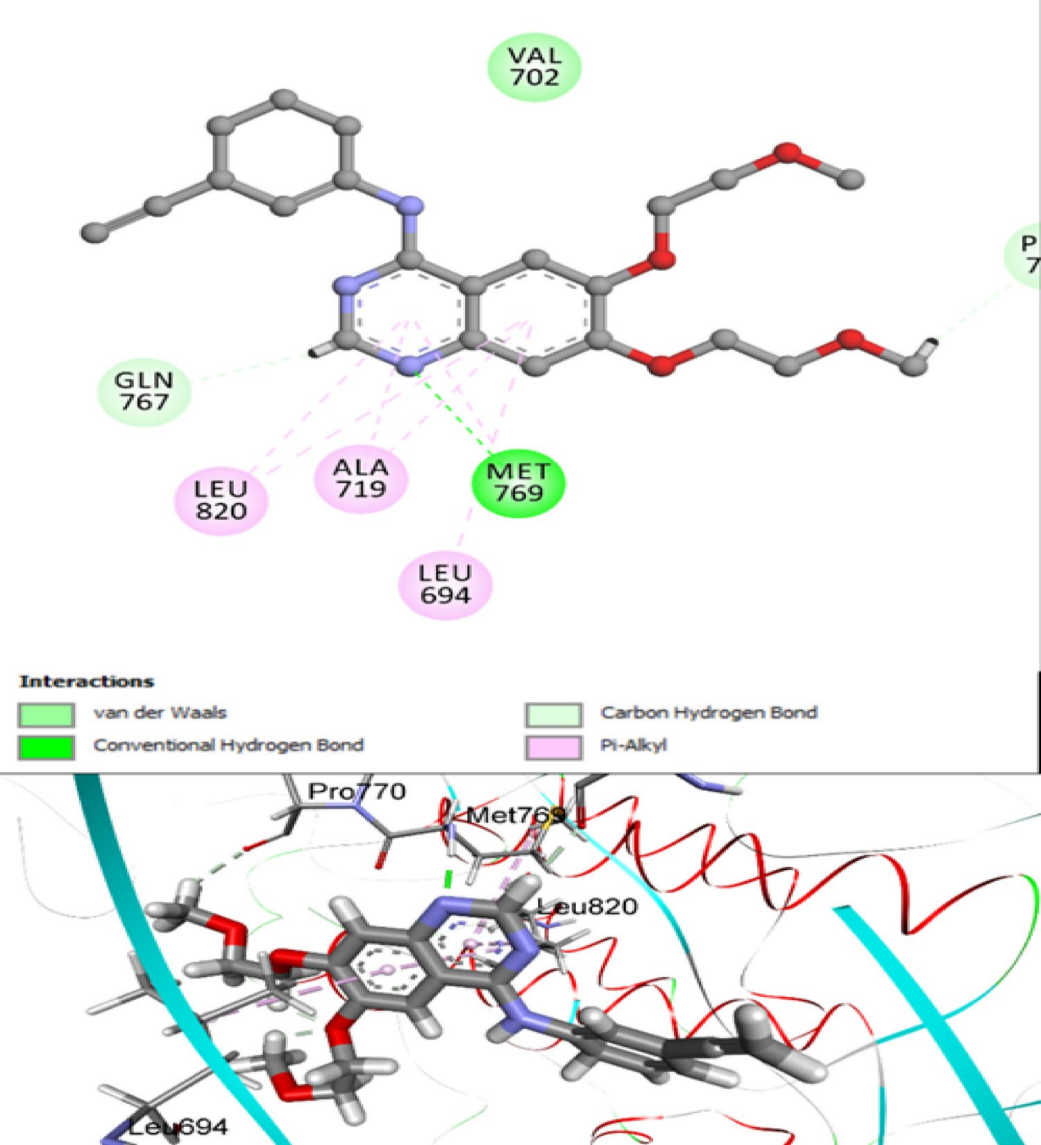
Validation of the docking protocol through the superimposition of erlotinib re-docked into the EGFR active site (PDB ID: 1M17) with its crystallographic pose. The re-docked conformation closely overlaps the experimental orientation (RMSD = 1.31 Å), retaining the key hydrogen bond between the pyrimidine nitrogen and the hinge residue Met769. This concordance confirms the accuracy and reliability of the docking methodology for predicting ligand binding modes

The redocking of erlotinib into the EGFR active site produced an RMSD value of 1.31 Å relative to its crystallographic conformation, with an S-score of − 7.73 kcal/mol. This result accurately reproduced the native binding orientation, including the critical hydrogen bond between the pyrimidine nitrogen and the hinge-region residue Met769. Such agreement between the docked and experimental poses confirms the validity and robustness of the docking protocol (Fig. 4).

**Fig. 5:**
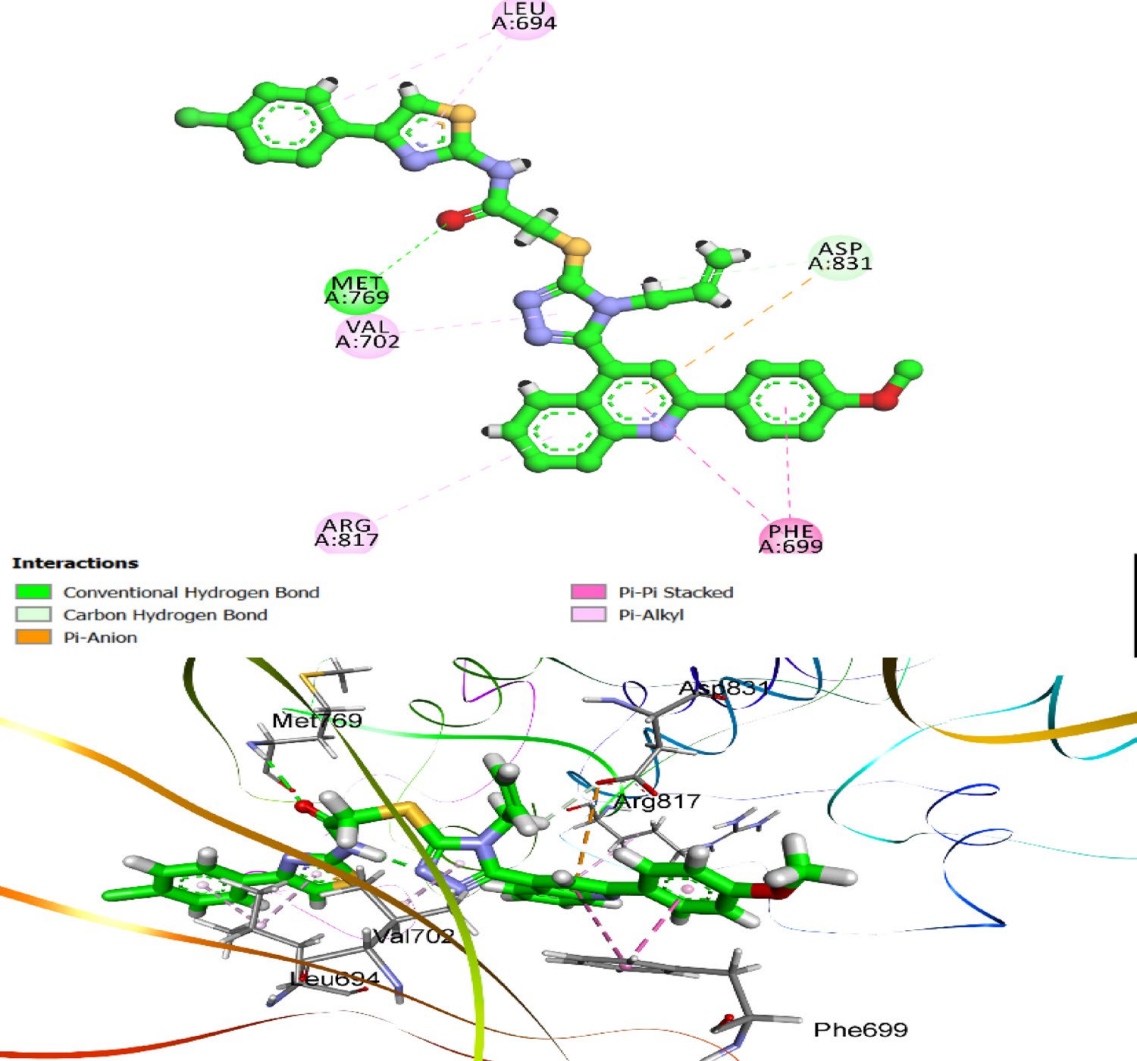
Docking pose of compound **8f** in the ATP-binding site of EGFR (PDB ID: 1M17). The ligand is anchored via a hinge hydrogen bond between its linker amide motif and Met769, while the quinoline core engages in a π–anion interaction with Asp831. The methoxyphenyl substituent on the quinoline forms π–π stacking with Phe699, and the thiazole ring establishes π–alkyl contacts with Leu694. The triazole ring interacts hydrophobically with Val702, and the *p*-chlorophenyl group occupies a lipophilic subpocket, enhancing van der Waals interactions. Additional stabilization is provided by π–π-alkyl contacts with Arg817. Docking score: − 8.21 kcal/mol; RMSD: 1.37 Å

Docking of compound **8f** into the ATP-binding site of EGFR resulted in a docking score of − 8.21 kcal/mol and an RMSD of 1.37 Å from the reference pose, indicating a reliable and well-aligned binding mode. The ligand is positioned deeply within the ATP-binding cleft, where the linker amide motif forms a key hydrogen bond with the backbone of Met769 in the hinge region, an anchoring interaction characteristic of potent EGFR inhibitors (Fig. 5).

The quinoline core engages in a π–π-anion interaction with Asp831, contributing an additional polar stabilization point in the active site. The methoxyphenyl substituent on the quinoline participates in π–π stacking with Phe699, further stabilizing the aromatic region of the ligand within the binding pocket. The thiazole ring forms hydrophobic π–π-alkyl contacts with Leu694, anchoring the terminal portion of the molecule in a lipophilic subpocket, while the triazole ring engages in hydrophobic interactions with Val702, reinforcing the scaffold’s spatial fit. The *p*-chlorophenyl group linked to the thiazole also occupies the hydrophobic subpocket, enhancing van der Waals interactions and matching SAR observations that *para*-chloro substitution increases potency. Additional quinoline-π–alkyl interactions with Arg817 further stabilize the complex.

Collectively, these interactions map directly onto the designed pharmacophore: the quinoline aromatic anchor (π–anion with Asp831 and π–alkyl with Arg817), the methoxyphenyl aromatic extension (π–π with Phe699), the triazole (hydrophobic with Val702), the amide hydrogen bond donor (hinge anchoring with Met769), and the thiazole–p-chlorophenyl hydrophobic moiety (contacts with Leu694). This comprehensive binding profile explains the experimentally observed high potency of compound 8f (EGFR IC₅₀ = 67 nM).

**Fig. 6:**
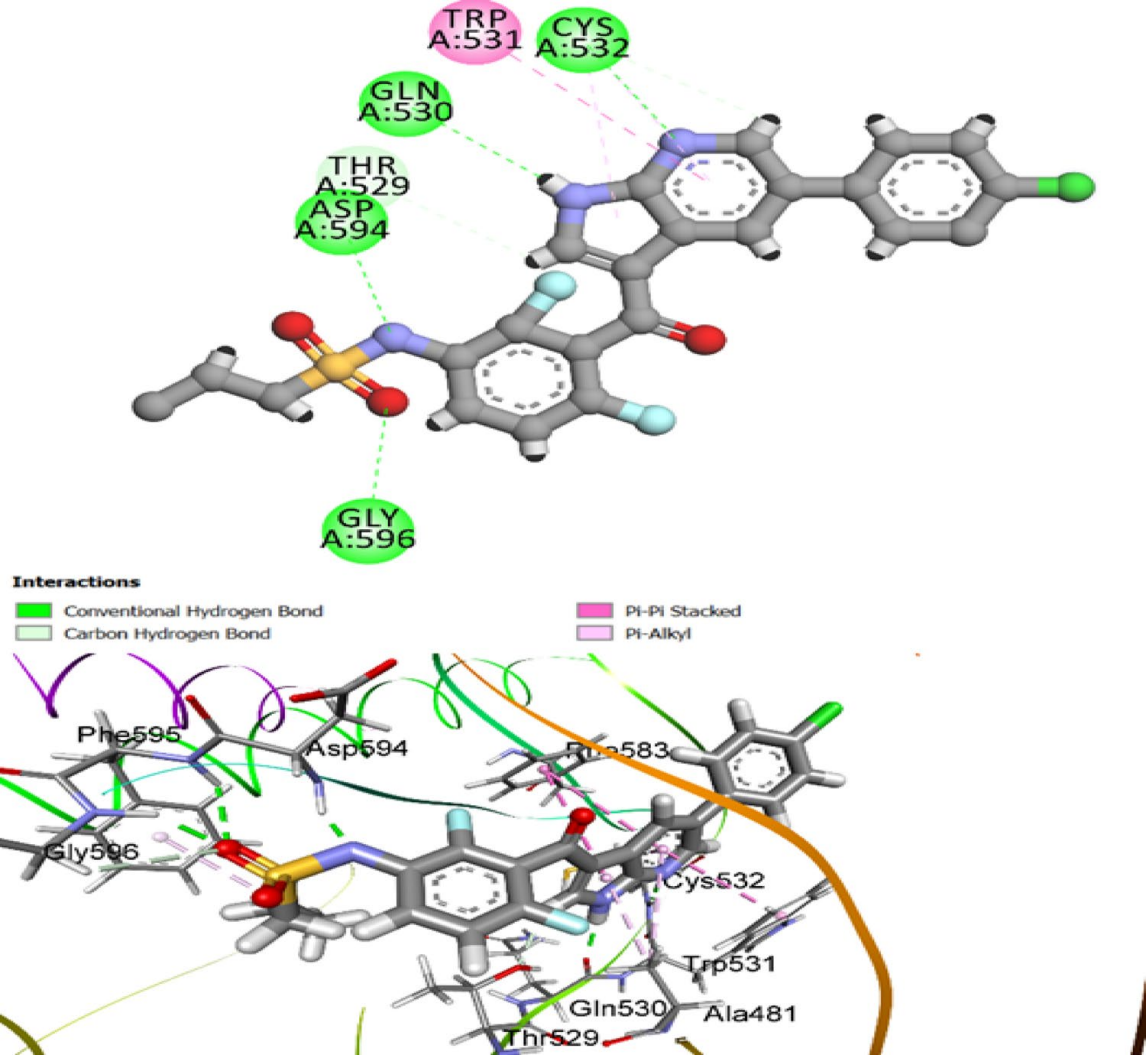
Validation of the docking protocol by redocking vemurafenib into the BRAF^V600E^ active site (PDB ID: 3OG7). The reproduced pose closely matches the crystallographic orientation (RMSD = 1.51 Å) and retains key hydrogen bonds with Cys532, Gly596, Thr529, and Asp594. Additional π–π stacking and π–alkyl interactions with Trp531 and neighboring hydrophobic residues further stabilize the complex. The favorable docking score (–9.34 kcal/mol) confirms the reliability of the docking methodology for predicting ligand binding modes

To validate the docking procedure for BRAF^V600E^, the co-crystallized ligand vemurafenib was re-docked into its binding pocket (PDB ID: 3OG7). The redocking produced a docking score of − 9.34 kcal/mol and an RMSD of 1.51 Å relative to the experimental pose, indicating excellent agreement and confirming the robustness of the docking protocol. The reproduced binding orientation preserved the critical hydrogen bonds with Cys532 and Gly596, key interactions that stabilize inhibitors within the ATP-binding site. These results substantiate the reliability of the docking methodology and provide a solid framework for subsequent analysis of the binding interactions of the most potent compound **8f** with BRAF^V600E^ (Fig. 6).

**Fig. 7:**
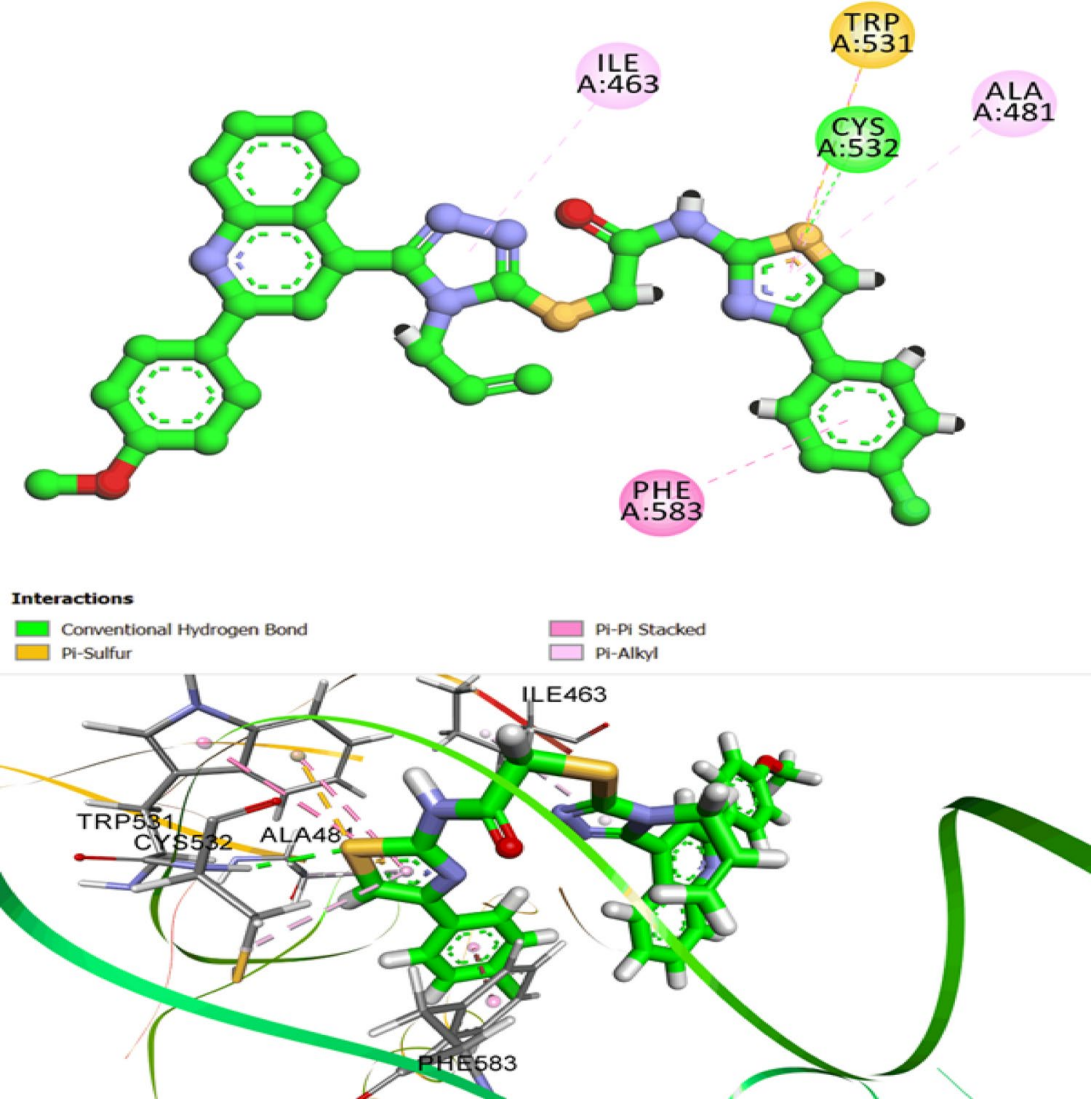
Docking pose of compound **8f** in the ATP-binding site of BRAF^V600E^ (PDB ID: 3OG7). The thiazole ring forms a conventional hydrogen bond with Cys532 and engages in π–π-sulfur interactions, π–π-π stacking with Trp531, and π–π-alkyl contacts with Ala481. The triazole ring interacts hydrophobically with Ile463, while the p-chlorophenyl group occupies a lipophilic subpocket through π–π-alkyl contacts with Phe583. The methoxyphenyl substituent extends toward the solvent-exposed channel. Docking score: − 7.54 kcal/mol; RMSD: 1.48 Å

Docking of **8f** into the ATP-binding site of BRAF^V600E^ yielded a docking score of − 7.54 kcal/mol and an RMSD of 1.48 Å from the reference pose, confirming a well-aligned and energetically favorable binding mode. The ligand is deeply accommodated within the active site, where the thiazole ring forms a conventional hydrogen bond with the backbone of Cys532, in addition to engaging in π–π-sulfur interactions with Trp531 as well as π–π-alkyl contacts with Ala481 (Fig. 7).

The triazole ring establishes π–π-alkyl interactions with Ile463, reinforcing scaffold positioning near the hinge region. The *p*-chlorophenyl group attached to the thiazole occupies a hydrophobic subpocket, forming π–π-alkyl interactions with Phe583, an interaction consistent with SAR trends showing potency enhancement by *para*-chloro substitution. The methoxyphenyl substituent extends toward the solvent interface. This binding arrangement matches the designed pharmacophore model, hydrogen bond anchoring via thiazole–Cys532 interaction, hydrophobic engagement through the *p*-chlorophenyl group, and hinge-adjacent stabilization by the triazole–Ile463 contact, thereby rationalizing the potent inhibitory activity of **8f** against BRAF^V600E^.

**Fig. 8:**
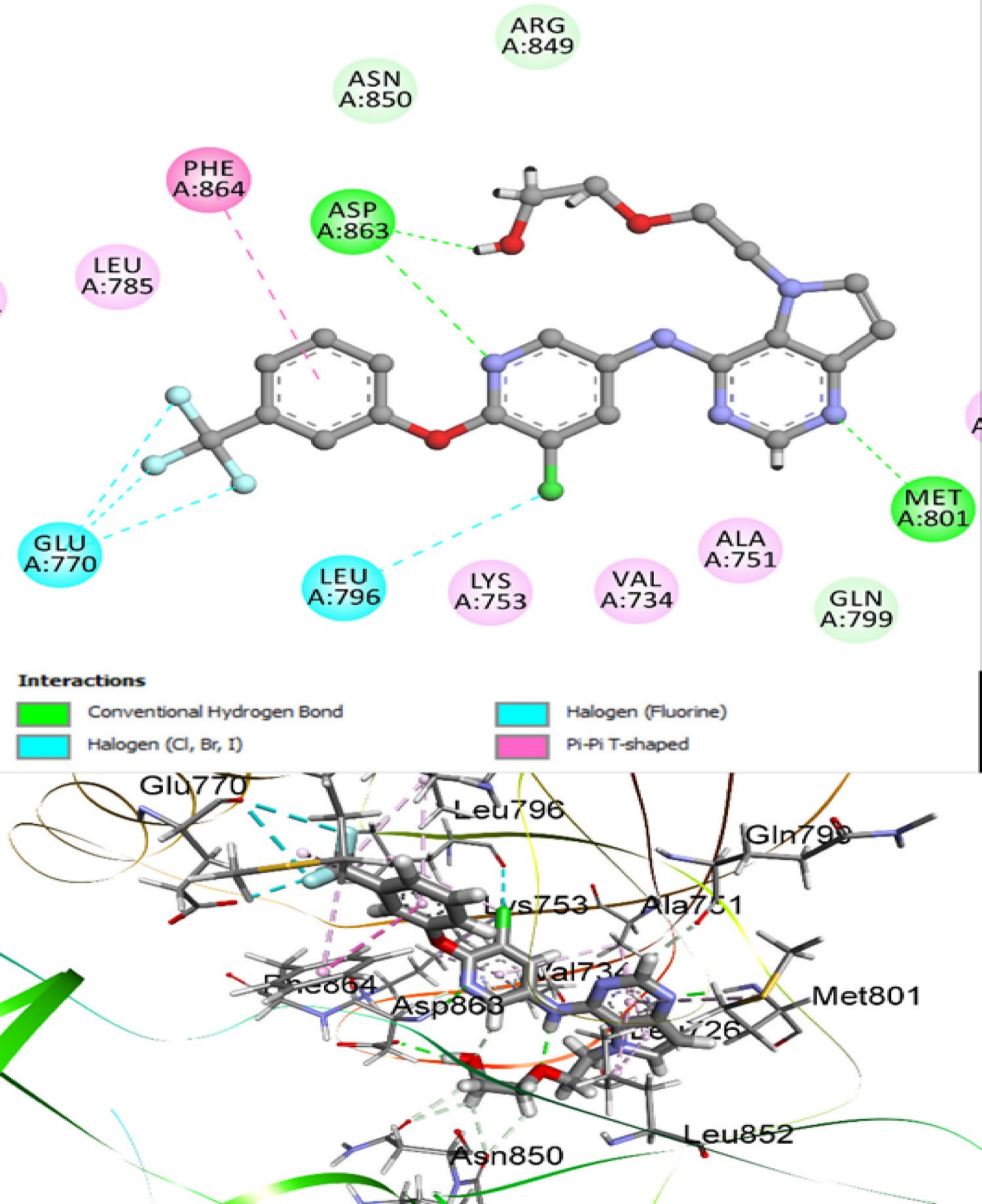
Validation of the docking protocol by redocking the co-crystallized ligand into the HER-2 active site. The reproduced pose closely matches the crystallographic orientation (RMSD = 1.31 Å) and yields a docking score of − 8.11 kcal/mol. Key stabilizing interactions include hydrogen bonds between the pyrimidine nitrogen and Met801, and between the pyridine nitrogen and Asp863, along with an additional hydrogen bond to Met801. π–π T-shaped stacking with Phe864 and halogen contacts involving Glu770 and Leu796 further reinforce ligand stability within the binding pocket

To validate the docking protocol for HER-2, the native co-crystallized ligand was re-docked into its binding pocket. The procedure yielded a binding affinity score (S-score) of − 8.11 kcal/mol and an RMSD of 1.31 Å relative to the crystallographic pose, demonstrating high accuracy of the docking approach. The reproduced orientation retained the critical hydrogen bond between the pyrimidine nitrogen of the ligand and the backbone of Met801, a key anchoring interaction within the HER-2 active site. Additional stabilizing contacts were observed, including a hydrogen bond between the pyridine nitrogen and Asp863, an extra hydrogen bond to Met801, π–π T-shaped stacking with Phe864, and halogen interactions involving Glu770 and Leu796. These results confirm the robustness of the docking methodology and provide a reliable framework for subsequent analysis of **8f** binding to HER-2 (Fig. 8).

**Fig. 9:**
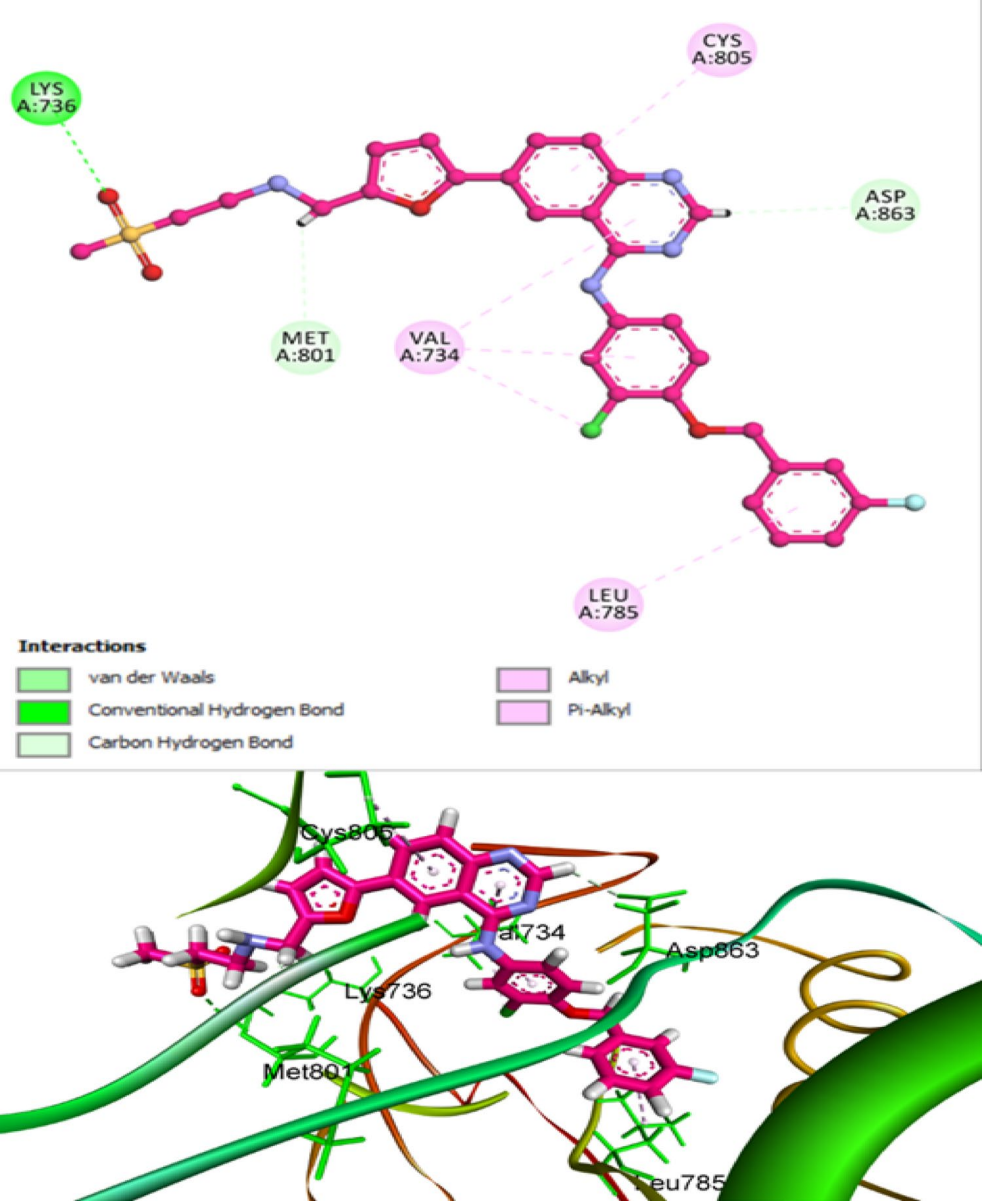
Predicted binding pose of lapatinib within the HER-2 active site. The reproduced orientation yields a docking score of − 8.42 kcal/mol and an RMSD of 0.98 Å, confirming a stable and well-aligned fit within the binding pocket. Key interactions include a hydrogen bond between the sulfone oxygen and Lys753, carbon–hydrogen bonds with Met801 and Asp863, and hydrophobic contacts with Leu785. π–alkyl interactions with Val734 further stabilize the complex, illustrating the tight and well-fitted accommodation of lapatinib in the HER-2 active site

Docking of lapatinib, a reference HER-2 inhibitor evaluated in the in vitro assays, was performed to characterize its interaction profile within the HER-2 active site. The docking produced a binding energy of − 8.42 kcal/mol and an RMSD of 0.98 Å relative to the crystallographic conformation, indicating a highly stable and accurate binding pose. The ligand is anchored through a key hydrogen bond between its sulfone oxygen and Lys753, which plays a central role in positioning within the pocket. Additional stabilization arises from carbon–hydrogen bonds with Met801 and Asp863, as well as strong hydrophobic contacts with Leu785 that contribute to a compact hydrophobic environment. π π-alkyl interactions with Val734 further enhance the molecular interaction network, reinforcing overall binding affinity. Collectively, these interactions rationalize the tight and stable accommodation of lapatinib within the HER-2 binding site, consistent with its potent inhibitory activity (Fig. 9).

**Fig. 10:**
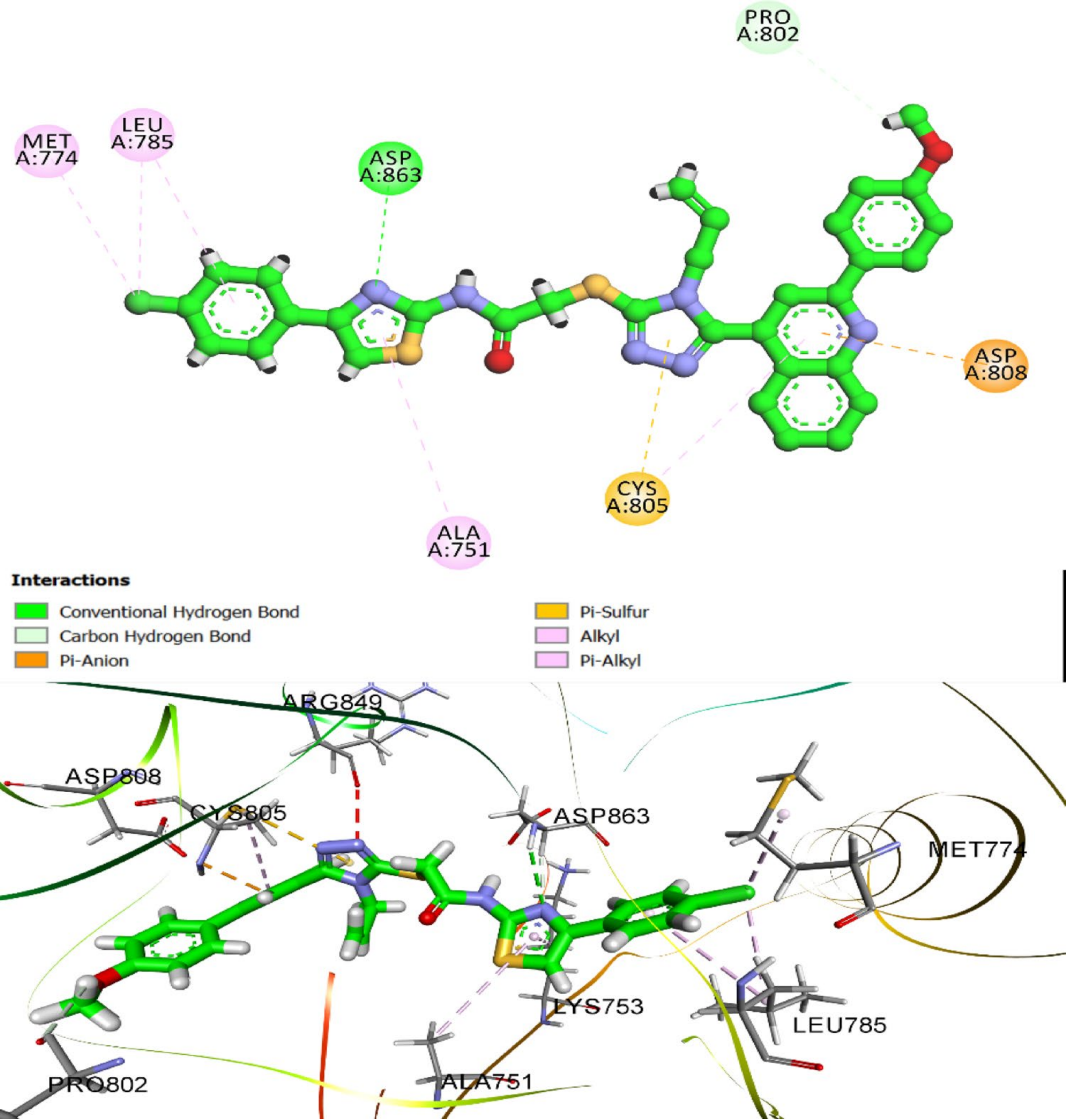
Docking pose of compound **8f** in the ATP-binding site of HER-2 (PDB ID: 3PP0). The thiazole ring forms a hydrogen bond with Asp863 and hydrophobic contacts with Ala751, while the triazole ring engages in a π–sulfur interaction with Cys805. The quinoline core establishes a π–anion interaction with Asp808, and its methoxyphenyl substituent forms a carbon–hydrogen bond with Pro802. The p-chlorophenyl group interacts hydrophobically with Leu785 and Met774, occupying a lipophilic subpocket. Docking score: − 7.97 kcal/mol; RMSD: 1.71 Å

Docking of compound **8f** into the ATP-binding site of HER-2 produced a docking score of − 7.97 kcal/mol and an RMSD of 1.71 Å from the reference pose, indicating a favorable and well-aligned binding orientation. The ligand adopts a conformation that allows its pharmacophoric features to engage key residues within the active site. The thiazole ring interacts with Asp863 through a conventional hydrogen bond and forms hydrophobic contacts with Ala751, anchoring this moiety in a polar-hydrophobic interface (Fig. 10**)**. The triazole ring establishes a π–sulfur interaction with Cys805, contributing to hinge-proximal stabilization of the scaffold. The quinoline core forms a π–anion interaction with Asp808, positioning the aromatic system within the adenine-binding region. The p-chlorophenyl group engages in hydrophobic π–alkyl contacts with Leu785 and Met774, fitting into a lipophilic subpocket that complements the halogen-substituted ring. While the methoxyphenyl substituent extends toward Pro802, forming a carbon–hydrogen bond that supports solvent-channel orientation and enhances molecular stability. Collectively, these interactions align with the designed pharmacophoric framework—hydrogen bond anchoring via thiazole–Asp863, hinge-proximal π–sulfur contact through triazole–Cys805, aromatic and electrostatic stabilization through the quinoline–Asp808 interaction, solvent-channel adaptation via the methoxyphenyl–Pro802 contact, and hydrophobic locking by the p-chlorophenyl–Leu785/Met774 interactions—rationalizing the strong binding affinity of compound 8f toward HER-2 and its potential as a multi-target kinase inhibitor.

### Molecular dynamics simulation of the EGFR–8f complex

We selected EGFR for the MD simulations based on four converging criteria. (i) Biochemical potency: compound **8f** shows low-nanomolar inhibition of EGFR (IC₅₀ = 67 nM), fully consistent with its strong cellular antiproliferative profile and making EGFR a primary pharmacological driver in our system. (ii) Docking quality: among the three targets, EGFR delivered the most favorable docking score (8f S-score = − 8.21 kcal/mol) together with a well-aligned pose (RMSD = 1.37 Å) that preserves the canonical hinge H-bond to Met769 and maps cleanly onto our pharmacophore (quinoline–hinge region, triazole/amide anchor, thiazole–p-chlorophenyl hydrophobic plug). (iii) Protocol robustness and benchmarking: EGFR offers a high-quality co-crystal (PDB 1M17) and a clinically validated reference (erlotinib), for which redocking reproduced the crystallographic pose (RMSD 1.31 Å, S-score − 7.73 kcal/mol). This enables a rigorous, apples-to-apples MD benchmark (EGFR–8f vs. EGFR–erlotinib) under identical conditions.

MD simulations were performed using GROMACS 2023 to assess the stability and binding dynamics of the EGFR–**8f** complex in comparison with the EGFR–erlotinib reference [68]. The protein–ligand complex was prepared in UCSF Chimera, adding hydrogen atoms to maintain correct geometry and bonding. The CHARMM36 force field was applied to the protein, while ligand parameters were generated using the CHARMM General Force Field (CGenFF) via the ParamChem server [69]. All parameter penalty scores were below 10, indicating high reliability and no need for reparameterization. Structural integrity was verified by energy minimization and short equilibration runs, followed by visual inspection, with no abnormalities observed [70, 71].

Each complex was embedded in a periodic cubic simulation box solvated with TIP3P water, maintaining a 1 nm buffer, and neutralized with Na⁺/Cl⁻ ions at 150 mM [72]. Energy minimization was performed using the steepest descent algorithm. NVT equilibration for 100 ps at 300 K (V-rescale thermostat) and NPT equilibration for 100 ps at 1.0 bar (Parrinello–Rahman barostat) were carried out with position restraints on heavy atoms [73, 74]. Production MD was then run for 100 ns without restraints, with a 2 fs time step and trajectory snapshots saved every 10 ps. Bond lengths involving hydrogens were constrained using LINCS, and long-range electrostatics were treated with the Particle Mesh Ewald method (10 Å cutoff) [75, 76].

**Fig. 11:**
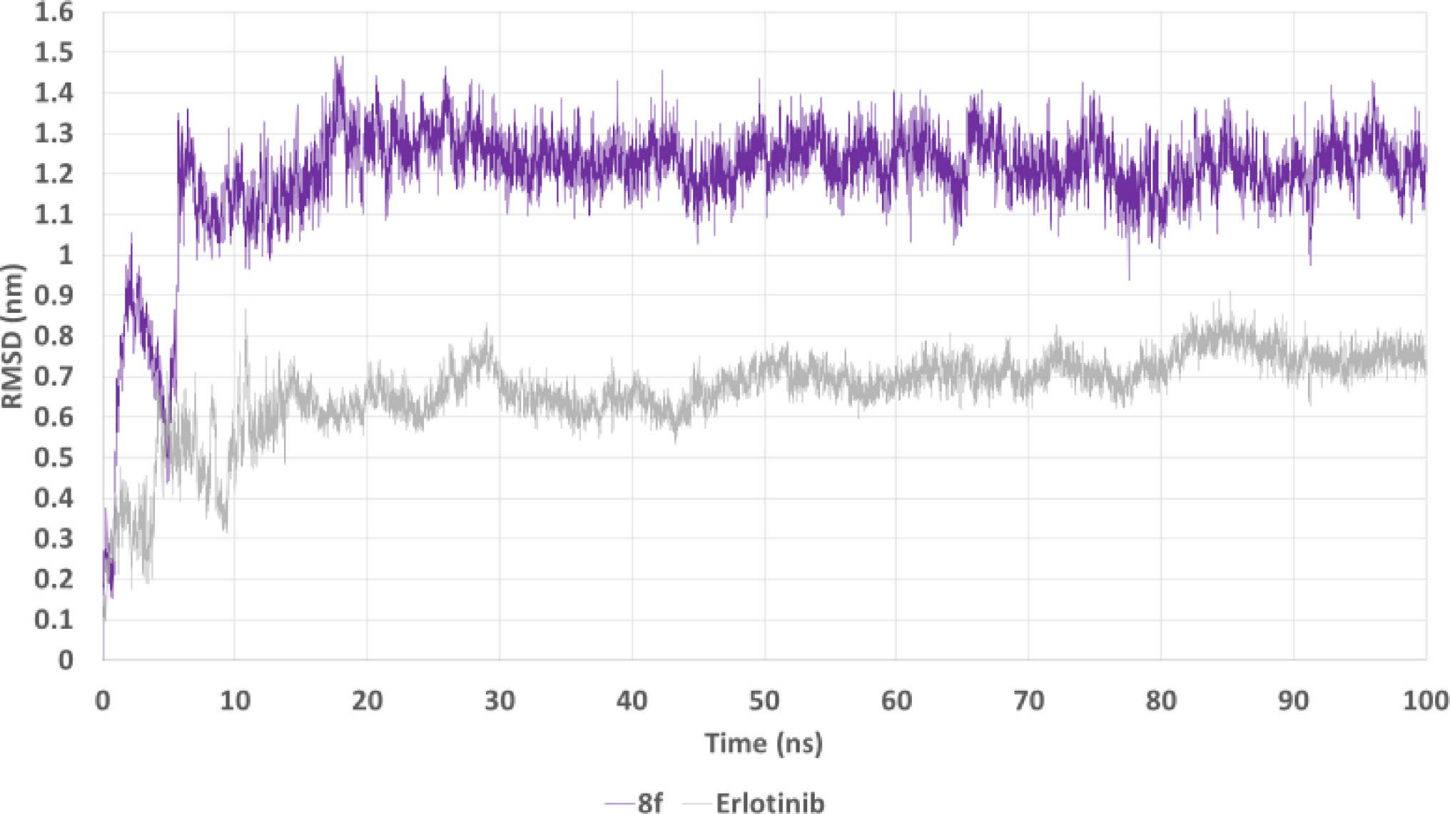
Backbone RMSD plots of EGFR–**8f** and EGFR–erlotinib complexes over 100 ns, showing stable plateaus after equilibration, with EGFR–8f exhibiting slightly higher RMSD due to pocket accommodation of its bulkier scaffold

Trajectory analysis revealed that both EGFR–**8f** and EGFR–erlotinib equilibrated quickly and maintained structural stability throughout the simulation. Backbone RMSD plots showed EGFR–**8f** stabilizing after ~ 8–10 ns, fluctuating narrowly between 1.15 and 1.30 nm, while erlotinib stabilized between 0.60 and 0.80 nm. The slightly higher RMSD for **8f** reflects an induced fit model of the ATP pocket to accommodate its bulkier scaffold (Fig. 11).

**Fig. 12:**
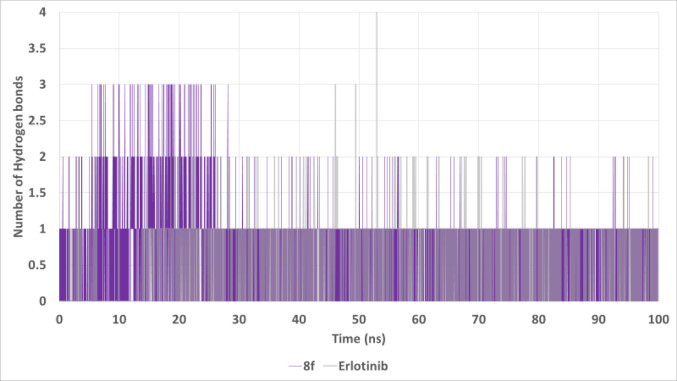
Hydrogen bond occupancy plots for EGFR–**8f** and EGFR–erlotinib over 100 ns, indicating persistent hinge H-bonding for 8f with occasional secondary contacts

Hydrogen bond occupancy analysis (Fig. 12) confirmed the persistence of the critical hinge interaction between the linker–amide moiety of 8f and Met769, as predicted by docking. This hydrogen bond remained intact for the majority of the simulation, occasionally complemented by transient secondary hydrogen bonds, enhancing local stability. In contrast, erlotinib displayed a more variable hydrogen bonding pattern, frequently alternating between one and two simultaneous interactions. The maintained hinge contact for 8f underscores the importance of this pharmacophoric element in securing the ligand within the binding region.

**Fig. 13:**
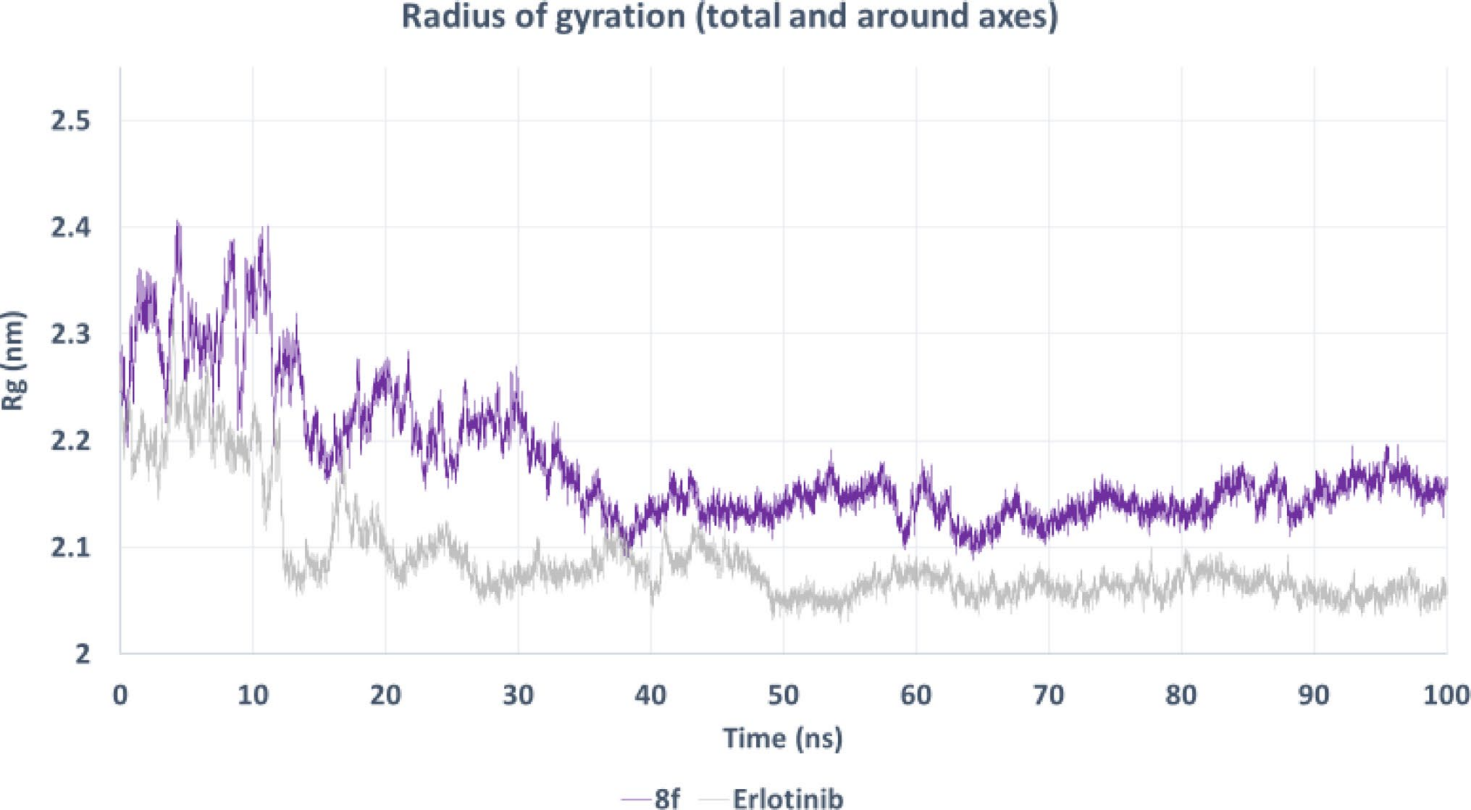
Radius of gyration (Rg) plots for EGFR–**8f** and EGFR–erlotinib complexes, demonstrating stable global compactness throughout the simulation

The radius of gyration (Rg) profile (Fig. 13) indicated that both complexes preserved their global compactness over time, with EGFR–**8f** exhibiting a slightly higher Rg (~ 2.15–2.25 nm) compared to EGFR–erlotinib (~ 2.08–2.12 nm). This marginal increase is consistent with the incorporation of the bulkier quinoline, methoxyphenyl, and *p*-chlorophenyl groups in **8f**, which extend into the hydrophobic subpockets while maintaining overall protein integrity.

**Fig. 14:**
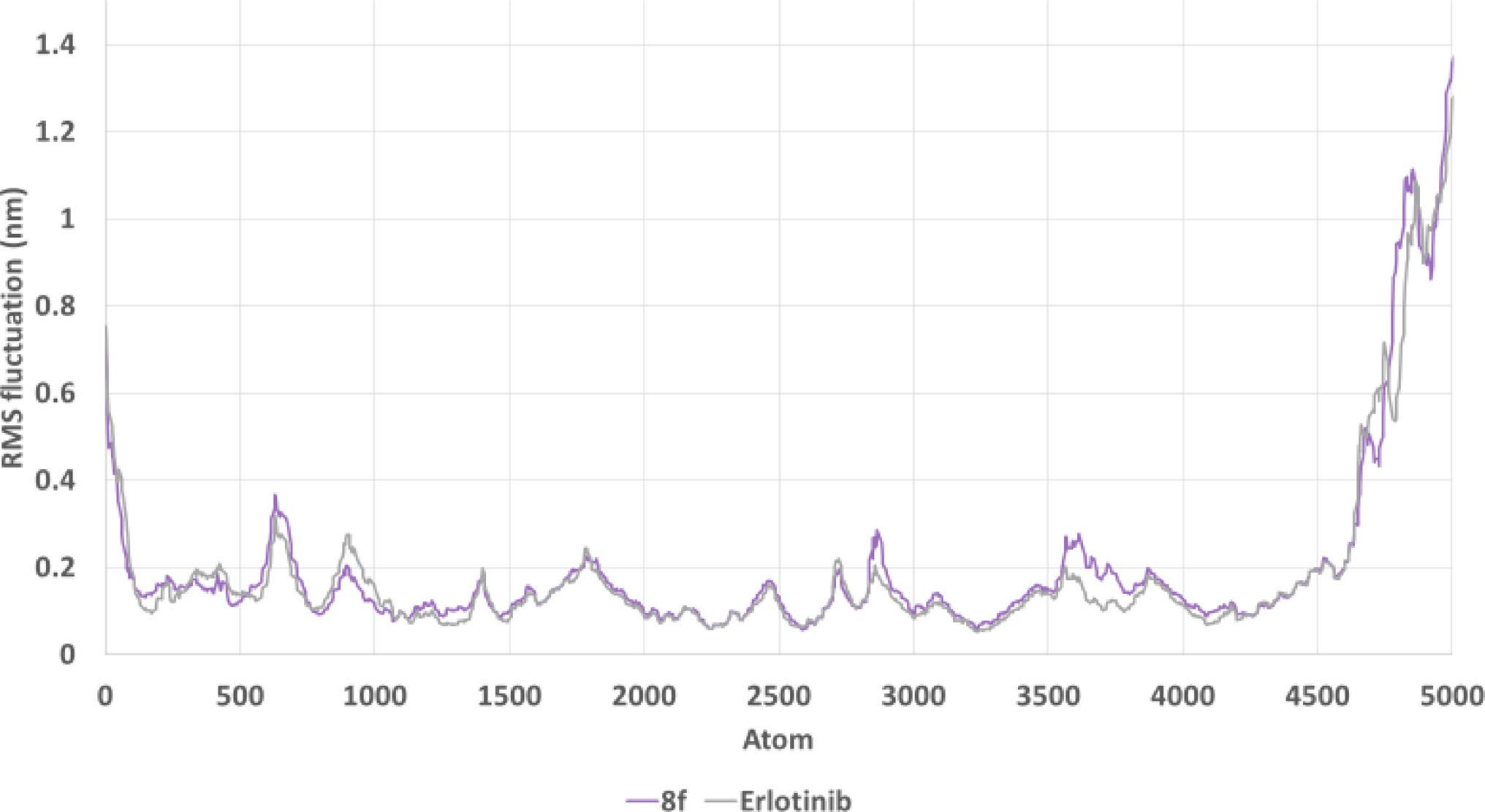
RMSF profiles of EGFR–**8f** and EGFR–erlotinib complexes, highlighting low fluctuations in hinge-binding residues, consistent with rigid binding cores

Root mean square fluctuation (RMSF) analysis (Fig. 14) demonstrated minimal deviations in the hinge region (Leu694, Val702, Met769), confirming that these pharmacophore-associated residues remain conformationally stable during the simulation. Peripheral loops displayed modest fluctuations, which are typical for solvent-exposed regions and not directly involved in ligand anchoring.

**Fig. 15:**
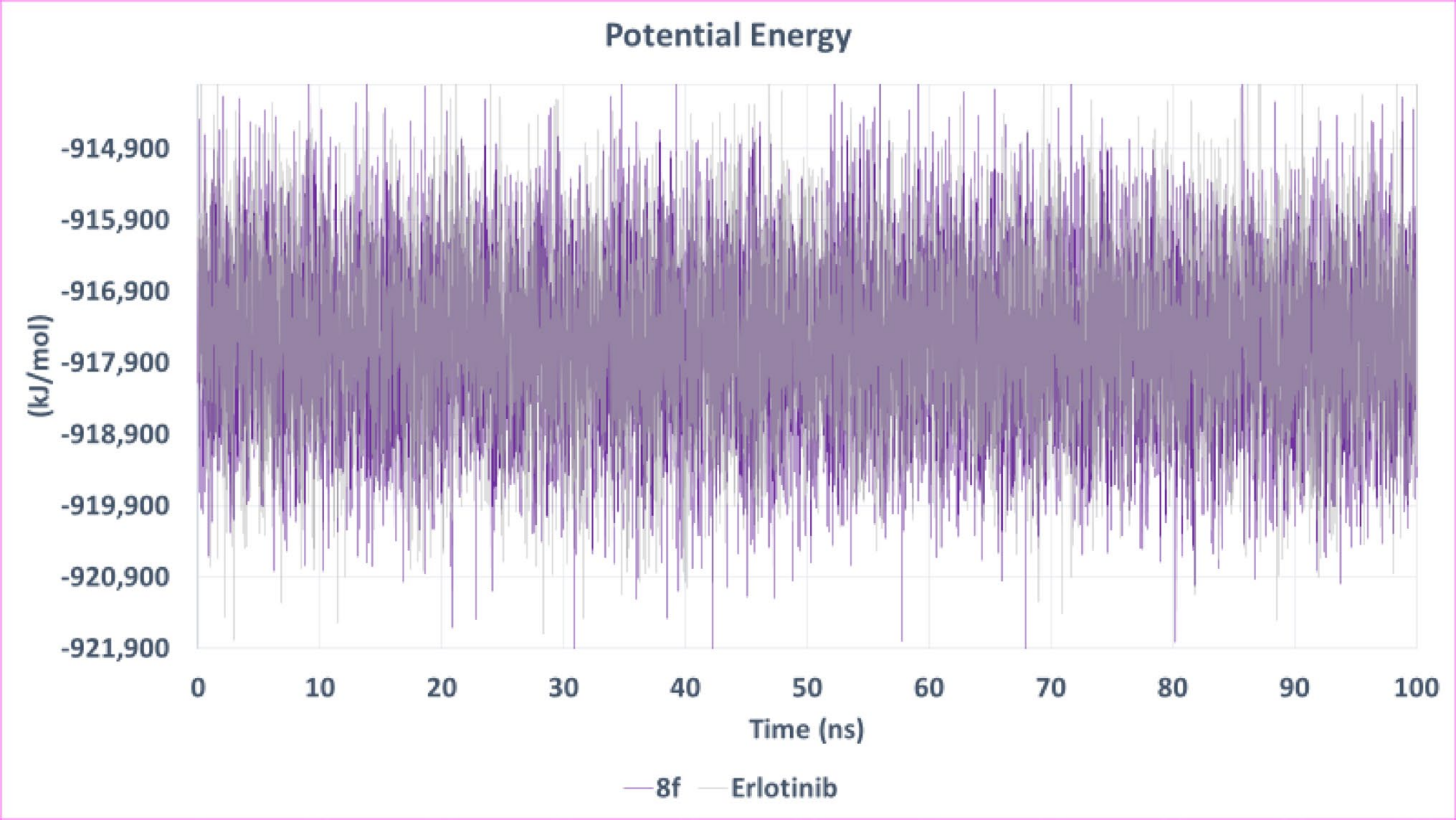
Potential energy trajectories for EGFR–**8f** and EGFR–erlotinib complexes, showing overlapping, stable energy profiles across the 100 ns simulation

The potential energy trajectories (Fig. 15) for EGFR–**8f** and EGFR–erlotinib were nearly superimposable, with no significant energy drift over 100 ns, indicating thermodynamically stable complexes under physiological simulation conditions.

These convergent computational data, together with the low-nanomolar biochemical activity of **8f**, identify this scaffold as a viable lead for further optimization toward multi-EGFR–BRAF^V600E^–HER-2 inhibition.

### In silico ADME assessment

To contextualize developability alongside target potency, we profiled compound **8f** using SwissADME and compared the outputs with the clinical benchmark erlotinib. **8f** presents as a large, highly lipophilic scaffold (MW 625.2 g·mol^−1^; consensus logP 6.11) with high polarity (TPSA 148.4 Å^2^), 11 rotatable bonds, six hydrogen-bond acceptors, and two donors. All solubility models converge on very low aqueous solubility (ESOL logS − 8.04; Ali − 10.08; SILICOS-IT − 12.08), and gastrointestinal absorption is predicted to be low.

The compound is not a P-gp substrate and is non-BBB permeant, but it is predicted to inhibit CYP2C9 and CYP3A4, flagging potential drug–drug interaction risk. Rule-based drug-likeness is only partially satisfied: Lipinski passes with one violation (MW > 500), whereas Ghose, Veber, Egan, and Muegge filters are not met (excess MW/logP, TPSA > 140 Å², and > 10 rotors); the bioavailability score is 0.55. Medicinal-chemistry alerts are minimal (PAINS 0; one Brenk alert, isolated alkene), and synthetic accessibility is moderate (4.21). Erlotinib, used here as a reference, exhibits an oral-drug-like profile (MW 393.4 g·mol^−1^; consensus logP 3.20; TPSA 74.7 Å^2^; 10 rotatable bonds) with model-dependent but acceptable solubility (ESOL logS − 4.11; Ali − 4.56; SILICOS-IT − 7.26) and predicted high GI absorption; it is not a P-gp substrate and is BBB permeant.

Erlotinib satisfies Lipinski, Ghose, Veber, Egan, and Muegge rules (bioavailability score 0.55), carries no PAINS alerts and one Brenk alert (alkyne), and is synthetically accessible (3.19). However, broad CYP inhibition (1A2/2C19/2C9/2D6/3A4) is predicted as expected for this chemotype. Taken together, the ADME comparison clarifies that the potency of 8f is accompanied by a suboptimal physicochemical envelope, simultaneously high MW, high lipophilicity, and high TPSA, which collectively drive poor solubility and low predicted intestinal absorption and raise the likelihood of CYP2C9/3A4-mediated interactions.

In line to advance **8f** as a multi-kinase lead, these data define concrete optimization axes: reduce size and lipophilicity (target consensus logP ~ 2–4 and MW ≤ 550 g·mol^−1^), trim rotatable bonds (≤ 8–10), and introduce solubilizing polarity while preserving the validated hinge-binding and hydrophobic pharmacophore. If needed for near-term in vivo work, lipid-based systems or nanosuspensions could bridge the solubility gap during SAR refinement.

## Conclusion

The present article discloses a new class of quinoline-based compounds that were particularly developed to simultaneously block EGFR, HER-2, and BRAF^V600E^, implying that they could be effective in cancer treatment. All target compounds underwent initial in vitro screening against four cancer cell lines. Compounds **8f** and **8k** were shown to be the most potent and selectively active derivatives on both the enzyme and cellular levels. Apoptotic marker tests found that **8f** and **8k** increased apoptotic markers such as caspases 3, 8, and 9, p53, and Bax while decreasing anti-apoptotic Bcl-2. Docking and 100-ns MD confirm a stable, pharmacophore-consistent binding mode for 8f with sustained hinge engagement at EGFR. SwissADME indicates oral-developability risks, size, lipophilicity, solubility, and CYP liability, guiding optimization toward reduced MW/logP and improved permeability without disrupting the hinge-binding motif.

## Experimental

### Chemistry

#### General details

See Appendix A (Supplementary File).

Compounds **1a-d** [47], **2a-d** [47], **3a-d** [47], **4a-d** [39], **6a-d** [48, 49], and **7a-d** [50] were prepared according to reported procedures and were confirmed by comparing their melting points with the reported ones.

#### General procedure for the synthesis of compounds (8a-o)

An equimolar mixture of compounds **4a-d**, **7a-d**, and TEA (1.2 mmol) in acetonitrile (50 mL) was stirred at room temperature for 4–8 h. The reaction progress was monitored *via* TLC (eluent DCM/Ethyl acetate 9:1). The resultant precipitate was filtered, dried, and subsequently recrystallized from aqueous ethanol, yielding the pure new compounds **8a-o**.

##### 2-((4-Allyl-5-(2-phenylquinolin-4-yl)-4*H*-1,2,4-triazol-3-yl)thio)-*N*-(4-phenylthiazol-2-yl) acetamide (8a)

Buff solid (70% yield); R_f_ = 0.31; m.p: 266–268 °C; ^1^H NMR (400 MHz, DMSO-*d*_*6*_) δ (ppm): 4.40 (2 H, s, SC*H*_*2*_), 4.65 (2 H, d, *J* = 3.5 Hz, NC*H*_*2*_), 4.84 (1H, d, *J*_*trans*_ = 17.2 Hz, N-CH_2_CH = C*H*_*2*_), 5.13 (1H, d, *J*_*cis*_ = 10.7 Hz, N-CH_2_CH = C*H*_*2*_), 5.82–5.91 (1H, m, C*H* = CH_2_), 7.36 (1H, d, *J* = 7.3 Hz, Ar-H), 7.42–7.51 (3 H, m, Ar-H), 7.59 (2 H, d, *J* = 7.5 Hz, Ar-H), 7.70 (1H, s, Ar-H), 7.87 (2 H, d, *J* = 8.3 Hz, Ar-H), 7.94 (2 H, d, *J* = 7.4 Hz, Ar-*H*), 8.03 (1H, d, *J* = 7.5 Hz, Ar-H), 8.21 (1H, d, *J* = 8.3 Hz, Ar-*H*), 8.29–8.37 (3 H, m, Ar-*H*), 12.68 (1H, s, N*H*); ^13^C NMR (100 MHz, DMSO-*d*_*6*_) δ (ppm): 36.8, 47.2, 108.8, 114.3, 118.0, 120.9, 125.3, 125.7, 126.1, 127.7, 128.3, 129.2, 129.4, 130.1, 130.5, 131.1, 132.6, 134.6, 138.3, 147.2, 148.4, 149.4, 150.5, 151.4, 152.6, 156.0, 166.9; Anal. calcd. for C_31_H_24_N_6_OS_2_: C, 66.41; H, 4.31; N, 14.99; S, 11.44; Found: C, 66.70; H, 4.49; N, 15.17; S, 11.65.

##### 2-((4-Allyl-5-(2-phenylquinolin-4-yl)-4*H*-1,2,4-triazol-3-yl)thio)-*N*-(4-(4-chlorophenyl) thiazol-2-yl)acetamide (8b)

White solid (75% yield); R_f_ = 0.3; m.p: 208–210 °C; ^1^H NMR (400 MHz, DMSO-*d*_*6*_) δ (ppm): 4.41 (2 H, s, SC*H*_*2*_), 4.65 (2 H, d, *J* = 4.0 Hz, NC*H*_*2*_), 4.85 (1H, d, *J*_*trans*_ = 17.2 Hz, N-CH_2_CH = C*H*_*2*_), 5.13 (1H, d, *J*_*cis*_ = 10.4 Hz, N-CH_2_CH = C*H*_*2*_), 5.55–6.04 (1H, m, C*H* = CH_2_), 7.52 (2 H, d, *J* = 8.4 Hz, Ar-H), 7.54–7.63 (3 H, m, Ar-H), 7.65 (1H, d, *J* = 7.8 Hz, Ar-H), 7.76 (1H, s, Ar-H), 7.87 (2 H, d, *J* = 8.0 Hz, Ar-H), 7.95 (2 H, d, *J* = 8.4 Hz, Ar-*H*), 8.21 (1H, d, *J* = 8.6 Hz, Ar-*H*), 8.26–8.83 (3 H, m, Ar-*H*), 12.73 (1H, s, N*H*); ^13^C NMR (100 MHz, DMSO-*d*_*6*_) δ (ppm): 36.7, 47.2, 109.6, 118.0, 120.1, 125.3, 125.6, 127.7, 127.8, 128.1, 129.2, 129.4, 130.2, 130.5, 131.1, 132.6, 132.8, 133.5, 134.0, 138.3, 148.2, 148.4, 151.3, 152.5, 156.0, 158.3, 166.9; Anal. calcd. for C_31_H_23_ClN_6_OS_2_: C, 62.56; H, 3.90; N, 14.12; S, 10.77; Found: C, 62.77; H, 4.13; N, 14.35; S, 10.85.

##### 2-((4-Allyl-5-(2-phenylquinolin-4-yl)-4*H*-1,2,4-triazol-3-yl)thio)-*N*-(4-(4-nitrophenyl) thiazol-2-yl)acetamide (8c)

Yellow solid (72% yield); R_f_ = 0.27; m.p: 245–247 °C; ^1^H NMR (400 MHz, DMSO-*d*_*6*_) δ (ppm): 4.42 (2 H, s, SC*H*_*2*_), 4.65 (2 H, d, *J* = 4.3 Hz, NC*H*_*2*_), 4.85 (1H, d, *J*_*trans*_ = 17.3 Hz, N-CH_2_CH = C*H*_*2*_), 5.13 (1H, d, *J*_*cis*_ = 10.5 Hz, N-CH_2_CH = C*H*_*2*_), 5.79–5.93 (1H, m, C*H* = CH_2_), 7.52–7.58 (2 H, m, Ar-H), 7.59–7.63 (1H, m, Ar-H), 7.65 (1H, d, *J* = 8.2 Hz, Ar-H), 7.80–7.94 (2 H, m, Ar-H), 8.07 (1H, s, Ar-H), 8.16–8.23 (3 H, m, Ar-*H*), 8.28–8.32 (2 H, m, Ar-*H*), 8.33–8.35 (3 H, m, Ar-*H*), 12.83 (1H, s, N*H*); ^13^C NMR (100 MHz, DMSO-*d*_*6*_) δ (ppm): 36.8, 47.2, 113.3, 118.0, 120.2, 124.8, 125.3, 125.7, 127.1, 127.8, 128.1, 129.5, 130.2, 130.6, 131.1, 132.6, 134.1, 138.4, 140.7, 147.0, 147.3, 148.5, 151.4, 152.6, 156.1, 158.9, 167.2; Anal. calcd. for C_31_H_23_N_7_O_3_S_2_: C, 61.47; H, 3.83; N, 16.19; S, 10.59; Found: C, 61.68; H, 3.97; N, 16.35; S,10.62.

##### 2-((4-Allyl-5-(2-phenylquinolin-4-yl)-4*H*-1,2,4-triazol-3-yl)thio)-*N*-(4-(p-tolyl)thiazol-2-yl) acetamide (8d)

White solid (64% yield); R_f_ = 0.29; m.p: 168–170 °C; ^1^H NMR (400 MHz, DMSO-*d*_*6*_) δ (ppm): 2.34 (3 H, s, C*H*_*3*_), 4.40 (2 H, s, SC*H*_*2*_), 4.64 (2 H, d, *J* = 3.5 Hz, NC*H*_*2*_), 4.85 (1H, d, *J*_*trans*_ = 17.3 Hz, N-CH_2_CH = C*H*_*2*_), 5.13 (1H, d, *J*_*cis*_ = 10.5 Hz, N-CH_2_CH = C*H*_*2*_), 5.79–5.94 (1H, m, C*H* = CH_2_), 7.26 (2 H, d, *J* = 8.0 Hz, Ar-H), 7.50–7.60 (3 H, m, Ar-H), 7.62 (1H, s, Ar-H), 7.65 (1H, d, *J* = 7.8 Hz, Ar-H), 7.82 (2 H, d, *J* = 8.0 Hz, Ar-H), 7.87 (2 H, d, *J* = 8.0 Hz, Ar-*H*), 8.21 (1H, d, *J* = 8.3 Hz, Ar-*H*), 8.28–8.37 (3 H, m, Ar-*H*), 12.70 (1H, s, N*H*); ^13^C NMR (100 MHz, DMSO-*d*_*6*_) δ (ppm): 21.3, 36.7, 47.2, 108.0, 118.0, 120.2, 125.3, 125.7, 126.1, 127.8, 128.1, 129.5, 129.8, 130.2, 130.6, 131.1, 132.0, 132.6, 134.1, 137.7, 138.4, 148.5, 149.6, 151.4, 152.6, 156.1, 158.1, 166.8; Anal. calcd. for C_32_H_26_N_6_OS_2_: C, 66.88; H, 4.56; N, 14.62; S, 11.16; Found: C, 67.09; H, 4.63; N, 14.89; S, 11.09.

##### 2-((4-Allyl-5-(2-(4-methoxyphenyl)quinolin-4-yl)-4*H*-1,2,4-triazol-3-yl)thio)-*N*-(4-phenylthiazol-2-yl)acetamide (8e)

Buff solid (60% yield); R_f_ = 0.31; m.p: 228–230 °C; ^1^H NMR (400 MHz, DMSO-*d*_*6*_) δ (ppm): 3.87 (3 H, s, OCH_3_), 4.40 (2 H, s, SC*H*_*2*_), 4.63 (2 H, d, *J* = 3.7 Hz, NC*H*_*2*_), 4.85 (1H, d, *J*_*trans*_ = 17.3 Hz, N-CH_2_CH = C*H*_*2*_), 5.14 (1H, d, *J*_*cis*_ = 11.5 Hz, N-CH_2_CH = C*H*_*2*_), 5.79–5.92 (1H, m, C*H* = CH_2_), 7.13 (2 H, d, *J* = 8.8 Hz, Ar-H), 7.35 (1H, t, *J* = 7.3 Hz, Ar-H), 7.46 (2 H, t, J = 7.7 Hz, Ar-H), 7.59 (1H, t, *J* = 7.6 Hz, Ar-H), 7.77 (1H, s, Ar-H), 7.78–7.90 (2 H, m, Ar-H), 7.94 (2 H, d, *J* = 7.5 Hz, Ar-*H*), 8.16 (1H, d, *J* = 8.4 Hz, Ar-*H*), 8.27 (1H, s, Ar-H), 8.29 (2 H, d, *J* = 8.8 Hz, Ar-H), 12.71 (1H, s, N*H*); ^13^C NMR (100 MHz, DMSO-*d*_*6*_) δ (ppm): 36.7, 47.2, 55.8, 108.9, 114.8, 118.0, 119.8, 125.0, 125.6, 126.2, 126.3, 127.6, 128.4, 129.3, 130.0, 130.8, 131.0, 132.6, 133.9, 134.7, 148.5, 149.5, 151.3, 152.7, 155.8, 158.2, 161.5, 166.9; Anal. calcd. for C_32_H_26_N_6_O_2_S_2_: C, 65.07; H, 4.44; N, 14.23; S, 10.85; Found: C, 65.34; H, 4.63; N, 14.51; S, 10.78.

##### 2-((4-Allyl-5-(2-(4-methoxyphenyl)quinolin-4-yl)-4*H*-1,2,4-triazol-3-yl)thio)-*N*-(4-(4-chlorophenyl)thiazol-2-yl)acetamide (8f)

White solid (64% yield); R_f_ = 0.28; m.p: 236–237 °C; ^1^H NMR (400 MHz, DMSO-*d*_*6*_) δ (ppm): 3.87 (3 H, s, OCH_3_), 4.40 (2 H, s, SC*H*_*2*_), 4.63 (2 H, d, *J* = 3.6 Hz, NC*H*_*2*_), 4.85 (1H, d, *J*_*trans*_ = 17.3 Hz, N-CH_2_CH = C*H*_*2*_), 5.13 (1H, d, *J*_*cis*_ = 10.4 Hz, N-CH_2_CH = C*H*_*2*_), 5.78–5.94 (1H, m, C*H* = CH_2_), 7.13 (2 H, d, *J* = 8.7 Hz, Ar-H), 7.52 (2 H, d, *J* = 8.4 Hz, Ar-H), 7.59 (1H, t, *J* = 7.5 Hz, Ar-H), 7.77 (1H, s, Ar-H), 7.80–7.88 (2 H, m, Ar-H), 7.95 (2 H, d, *J* = 8.4 Hz, Ar-*H*), 8.16 (1H, d, *J* = 8.3 Hz, Ar-*H*), 8.27 (1H, s, Ar-H), 8.29 (2 H, d, *J* = 8.7 Hz, Ar-H), 12.68 (1H, s, N*H*); ^13^C NMR (100 MHz, DMSO-*d*_*6*_) δ (ppm): 36.7, 47.2, 109.6, 118.0, 120.2, 125.3, 125.7, 127.8, 127.9, 128.1, 129.3, 129.5, 130.2, 130.6, 131.1, 132.6, 132.8, 133.5, 134.1, 138.4, 148.2, 148.5, 151.4, 152.6, 156.1, 158.4, 166.9; Anal. calcd. for C_32_H_25_ClN_6_O_2_S_2_: C 61.48, H 4.03, N 13.44, S 10.26. Found: C 61.70, H 4.21, N 13.69, S 10.38; MS (EI +) *m*/*z* 625.26 (M + H)^+^.

##### 2-((4-Allyl-5-(2-(4-methoxyphenyl)quinolin-4-yl)-4*H*-1,2,4-triazol-3-yl)thio)-*N*-(4-(4-nitrophenyl)thiazol-2-yl)acetamide (8 g)

Yellow solid (86% yield); R_f_ = 0.29; m.p: 267–269 °C; ^1^H NMR (400 MHz, DMSO-*d*_*6*_) δ (ppm): 3.87 (3 H, s, OCH_3_), 4.42 (2 H, s, SC*H*_*2*_), 4.63 (2 H, d, *J* = 3.6 Hz, NC*H*_*2*_), 4.86 (1H, d, *J*_*trans*_ = 17.1 Hz, N-CH_2_CH = C*H*_*2*_), 5.14 (1H, d, *J*_*cis*_ = 10.1 Hz, N-CH_2_CH = C*H*_*2*_), 5.76–5.92 (1H, m, C*H* = CH_2_), 7.13 (2 H, d, *J* = 8.2 Hz, Ar-H), 7.59 (1H, t, *J* = 7.5 Hz, Ar-H), 7.76–7.89 (2 H, m, Ar-H), 8.07 (1H, s, Ar-H), 8.15 (1H, d, *J* = 8.3 Hz, Ar-*H*), 8.19 (2 H, d, *J* = 8.2 Hz, Ar-*H*), 8.27 (2 H, d, *J* = 5.9 Hz, Ar-H), 8.29–8.35 (3 H, m, Ar-H), 12.80 (1H, s, N*H*); ^13^C NMR (100 MHz, DMSO-*d*_*6*_) δ (ppm): 36.7, 47.2, 55.8, 113.3, 114.8, 118.0, 119.8, 124.8, 125.0, 125.6, 127.1, 127.6, 129.3, 130.0, 130.8, 131.0, 132.6, 133.9, 140.6, 147.0, 147.3, 148.5, 151.3, 152.7, 155.8, 158.8, 161.5, 167.2; Anal. calcd. for C_32_H_25_N_7_O_4_S_2_: C, 60.46; H, 3.96; N, 15.42; S, 10.09; Found: C, 60.65; H, 3.78; N, 15.67; S, 10.24.

##### 2-((4-Allyl-5-(2-(4-methoxyphenyl)quinolin-4-yl)-4*H*-1,2,4-triazol-3-yl)thio)-*N*-(4-(p-tolyl) thiazol-2-yl)acetamide (8 h)

White solid (81% yield); R_f_ = 0.3; m.p: 243–245 °C; ^1^H NMR (400 MHz, CDCl_3_) δ (ppm): 2.3 (3 H, s, CH_3_), 3.87 (3 H, s, OCH_3_), 4.27 (2 H, s, SC*H*_*2*_), 4.47 (2 H, d, *J* = 4.0 Hz, NC*H*_*2*_), 4.99 (1H, d, *J*_*trans*_ = 17.0 Hz, N-CH_2_CH = C*H*_*2*_), 5.26 (1H, d, *J*_*cis*_ = 10.3 Hz, N-CH_2_CH = C*H*_*2*_), 5.64–5.85 (1H, m, C*H* = CH_2_), 7.03 (2 H, d, *J* = 8.3 Hz, Ar-H), 7.08 (1H, s, Ar-H), 7.14 (2 H, d, *J* = 7.9 Hz, Ar-*H*), 7.53 (1H, t, *J* = 7.5 Hz, Ar-*H*), 7.69 (2 H, d, J = 8.0 Hz, Ar-H), 7.76–7.83 (1H, m, Ar-H), 7.86 (1H, d, *J* = 8.3 Hz, Ar-*H*), 7.96 (1H, s, Ar-H), 8.17 (2 H, d, *J* = 8.2 Hz, Ar-*H*), 8.45 (1H, d, *J* = 8.1 Hz, Ar-*H*)); ^13^C NMR (100 MHz, CDCl_3_) δ (ppm): 21.4, 36.1, 47.8, 55.7, 107.1, 114.9, 119.9, 120.5, 125.0, 125.5, 126.1, 127.3, 128.1, 128.5, 129.1, 129.6, 130.4, 130.5, 130.7, 131.6, 132.3, 132.4, 138.4, 149.3, 152.6, 153.0, 155.4, 158.0, 162.7, 166.3; Anal. calcd. for C_33_H_28_N_6_O_2_S_2_: C, 65.54; H, 4.67; N, 13.90; S, 10.60; Found: C, 65.82; H, 4.72; N, 14.06; S, 10.71.

##### 2-((4-Allyl-5-(2-(4-chlorophenyl)quinolin-4-yl)-4*H*-1,2,4-triazol-3-yl)thio)-*N*-(4-phenylthiazol-2-yl)acetamide (8i)

Buff solid (63% yield); R_f_ = 0.27; m.p: 247–248 °C; ^1^H NMR (400 MHz, DMSO-*d*_*6*_) δ (ppm): 4.40 (2 H, s, SC*H*_*2*_), 4.64 (2 H, d, *J* = 3.6 Hz, NC*H*_*2*_), 4.84 (1H, d, *J*_*trans*_ = 17.3 Hz, N-CH_2_CH = C*H*_*2*_), 5.12 (1H, d, *J*_*cis*_ = 10.5 Hz, N-CH_2_CH = C*H*_*2*_), 5.76–5.91 (1H, m, C*H* = CH_2_), 7.35 (1H, t, *J* = 7.2 Hz, Ar-H), 7.46 (3 H, t, *J* = 7.6 Hz, Ar-H), 7.66 (3 H, d, *J* = 8.5 Hz, Ar-*H*), 7.70 (1H, s, Ar-H), 7.82–8.91 (2 H, m, Ar-H), 7.94 (2 H, d, *J* = 7.5 Hz, Ar-*H*), 8.21 (1H, d, *J* = 8.4 Hz, Ar-*H*), 8.36 (2 H, d, *J* = 8.1 Hz, Ar-*H*), 12.68 (1H, s, N*H*); ^13^C NMR (100 MHz, DMSO-*d*_*6*_) δ (ppm): 36.7, 47.2, 108.9, 118.1, 120.1, 125.3, 125.4, 125.7, 126.2, 128.4, 129.3, 129.5, 129.6, 130.2, 131.3, 132.6, 134.3, 134.7, 135.5, 137.2, 148.4, 149.5, 151.4, 152.5, 154.9, 158.2, 166.9; Anal. calcd. for C_31_H_23_ClN_6_OS_2_: C, 62.56; H, 3.90; N, 14.12; S, 10.77; Found: C, 62.78; H, 4.12; N, 14.30; S, 10.86.

##### 2-((4-Allyl-5-(2-(4-chlorophenyl)quinolin-4-yl)-4*H*-1,2,4-triazol-3-yl)thio)-*N*-(4-(4-chlorophenyl)thiazol-2-yl)acetamide (8j)

White solid (61% yield); R_f_ = 0.28; m.p: 230–231 °C; ^1^H NMR (400 MHz, DMSO-*d*_*6*_) δ (ppm): 4.41 (2 H, s, SC*H*_*2*_), 4.64 (2 H, d, *J* = 3.0 Hz, NC*H*_*2*_), 4.84 (1H, d, *J*_*trans*_ = 17.2 Hz, N-CH_2_CH = C*H*_*2*_), 5.12 (1H, d, *J*_*cis*_ = 10.4 Hz, N-CH_2_CH = C*H*_*2*_), 5.73–5.93(1H, m, C*H* = CH_2_), 7.52 (2 H, d, *J* = 8.4 Hz, Ar-H), 7.65 (3 H, d, *J* = 8.4 Hz, Ar-*H*), 7.76 (1H, s, Ar-H), 7.83–7.92 (2 H, m, Ar-*H*), 7.95 (2 H, d, *J* = 8.4 Hz, Ar-*H*), 8.20 (1H, d, *J* = 8.3 Hz, Ar-*H*), 8.35 (3 H, d, *J* = 7.8 Hz, Ar-*H*), 12.66 (1H, s, N*H*); ^13^C NMR (100 MHz, DMSO-*d*_*6*_) δ (ppm): 36.7, 47.2, 109.6, 118.1, 120.1, 125.4, 125.7, 127.9, 128.3, 129.3, 129.5, 129.6, 130.2, 131.2, 132.6, 132.8, 133.5, 134.3, 135.5, 137.2, 148.2, 148.4, 151.4, 152.5, 154.9, 158.4, 166.9; Anal. calcd. for C_31_H_22_Cl_2_N_6_OS_2_: C, 59.14; H, 3.52; N, 13.35; S, 10.18; Found: C, 59.38; H, 3.64; N, 13.61; S, 10.31.

##### 2-((4-Allyl-5-(2-(4-chlorophenyl)quinolin-4-yl)-4*H*-1,2,4-triazol-3-yl)thio)-*N*-(4-(4-nitrophenyl)thiazol-2-yl)acetamide (8k)

Yellow solid (68% yield); R_f_ = 0.29; m.p: 245–247 °C; ^1^H NMR (400 MHz, DMSO-*d*_*6*_) δ (ppm): 4.41 (2 H, s, SC*H*_*2*_), 4.65 (2 H, d, *J* = 3.3 Hz, NC*H*_*2*_), 4.84 (1H, d, *J*_*trans*_ = 17.1 Hz, N-CH_2_CH = C*H*_*2*_), 5.12 (1H, d, *J*_*cis*_ = 10.4 Hz, N-CH_2_CH = C*H*_*2*_), 5.78–5.91 (1H, m, C*H* = CH_2_), 7.66 (3 H, d, *J* = 8.5 Hz, Ar-H), 7.87 (1H, d, *J* = 8.3 Hz, Ar-H), 7.89–8.97 (1H, m, Ar-H), 8.07 (1H, s, Ar-H), 8.18–8.26 (4 H, m, Ar-H), 8.34 (4 H, t, *J* = 8.1 Hz, Ar-*H*), 12.58 (1H, s, N*H*); ^13^C NMR (100 MHz, DMSO-*d*_*6*_) δ (ppm): 36.8, 47.2, 113.3, 118.1, 120.1, 124.6, 124.8, 125.4, 126.9, 127.0, 128.3, 129.5, 129.6, 130.2, 131.3, 132.6, 134.3, 135.5, 137.2, 140.7, 147.0, 147.3, 148.4, 151.4, 152.5, 154.9, 159.0, 167.2; Anal. calcd. for C_31_H_22_ClN_7_O_3_S_2_: C, 58.17; H, 3.46; N, 15.32; S, 10.02; Found: C, 58.41; H, 3.62; N, 15.69; S, 10.29; MS (EI +) *m*/*z* 640.06 (M + H)^+^.

##### 2-((4-Allyl-5-(2-(4-chlorophenyl)quinolin-4-yl)-4*H*-1,2,4-triazol-3-yl)thio)-*N*-(4-tolylthiazol-2-yl)acetamide (8 L)

White solid (70% yield); R_f_ = 0.31; m.p: 210–212 °C; ^1^H NMR (400 MHz, DMSO-*d*_*6*_) δ (ppm): 2.34 (3 H, s, CH_3_), 4.40 (2 H, s, SC*H*_*2*_), 4.64 (2 H, d, *J* = 4.0 Hz, NC*H*_*2*_), 4.84 (1H, d, *J*_*trans*_ = 17.2 Hz, N-CH_2_CH = C*H*_*2*_), 5.11 (1H, d, *J*_*cis*_ = 10.5 Hz, N-CH_2_CH = C*H*_*2*_), 5.76–5.91 (1H, m, C*H* = CH_2_), 7.26 (2 H, d, *J* = 8.0 Hz, Ar-H), 7.62 (1H, s, Ar-H), 7.65 (3 H, d, *J* = 8.6 Hz, Ar-*H*), 7.82 (2 H, d, *J* = 8.0 Hz, Ar-*H*), 7.84–8.92 (2 H, m, Ar-H), 8.20 (1H, d, *J* = 8.3 Hz, Ar-*H*), 8.29–8.40 (3 H, m, Ar-H), 12.68 (1H, s, N*H*); ^13^C NMR (100 MHz, DMSO-*d*_*6*_) δ (ppm): 21.3, 36.7, 47.2, 108.0, 118.1, 120.1, 125.4, 125.7, 126.1, 128.3, 129.5, 129.6, 129.8, 130.2, 131.3, 132.0, 132.6, 134.3, 135.5, 137.2, 137.7, 148.4, 149.6, 151.4, 152.5, 154.9, 158.1, 166.8; Anal. calcd. for C_32_H_25_ClN_6_OS_2_: C, 63.10; H, 4.14; N, 13.80; S, 10.53; Found: C, 62.98; H, 4.37; N, 14.06; S, 10.67.

##### 2-((4-Allyl-5-(2-(4-(methylsulfonyl)phenyl)quinolin-4-yl)-4*H*-1,2,4-triazol-3-yl)thio)-*N*-(4-(4-chlorophenyl)thiazol-2-yl)acetamide (8 m)

White solid (75% yield); R_f_ = 0.3; m.p: 215–216 °C; ^1^H NMR (400 MHz, DMSO-*d*_*6*_) δ (ppm): 3.33 (3 H, s, SO_2_CH_3_), 4.42 (2 H, s, SC*H*_*2*_), 4.66 (2 H, d, *J* = 4.0 Hz, NC*H*_*2*_), 4.85 (1H, d, *J*_*trans*_ = 17.3 Hz, N-CH_2_CH = C*H*_*2*_), 5.12 (1H, d, *J*_*cis*_ = 10.5 Hz, N-CH_2_CH = C*H*_*2*_), 5.78–5.92 (1H, m, C*H* = CH_2_), 7.52 (2 H, d, *J* = 8.5 Hz, Ar-H), 7.70 (1H, t, *J* = 7.6 Hz, Ar-H), 7.77 (1H, s, Ar-H), 7.91 (1H, d, *J* = 8.6 Hz, Ar-H), 7.95 (3 H, t, *J* = 8.5 Hz, Ar-H), 8.14 (2 H, d, *J* = 8.5 Hz, Ar-H), 8.26 (1H, d, *J* = 8.3 Hz, Ar-H), 8.45 (1H, s, Ar-H), 8.58 (2 H, d, *J* = 8.5 Hz, Ar-H), 12.75 (1H, s, NH); ^13^C NMR (100 MHz, DMSO-d6) δ (ppm): 36.7, 43.9, 47.3, 109.6, 118.1, 120.6, 125.7, 125.8, 127.9, 128.1, 128.7, 128.8, 129.3, 130.4, 131.4, 132.6, 132.8, 133.5, 134.5, 142.2, 142.9, 148.2, 148.4, 151.5, 152.4, 154.5, 158.4, 166.9; Anal. calcd. for C_32_H_25_ClN_6_O_3_S_3_: C, 57.09; H, 3.74; N, 12.48; S, 14.29; Found: C, 57.30; H, 3.92; N, 12.71; S, 14.33; MS (EI +) *m*/*z* 673.69 (M + H)^+^.

##### 2-((4-Allyl-5-(2-(4-(methylsulfonyl)phenyl)quinolin-4-yl)-4*H*-1,2,4-triazol-3-yl)thio)-*N*-(4-(4-nitrophenyl)thiazol-2-yl)acetamide (8n)

White solid (81% yield); R_f_ = 0.28; m.p: 237–238 °C; ^1^H NMR (400 MHz, DMSO-*d*_*6*_) δ (ppm): 3.33 (3 H, s, SO_2_CH_3_), 4.43 (2 H, s, SC*H*_*2*_), 4.67 (2 H, d, *J* = 3.9 Hz, NC*H*_*2*_), 4.85 (1H, d, *J*_*trans*_ = 17.3 Hz, N-CH_2_CH = C*H*_*2*_), 5.12 (1H, d, *J*_*cis*_ = 10.5 Hz, N-CH_2_CH = C*H*_*2*_), 5.77–5.92 (1H, m, C*H* = CH_2_), 7.70 (1H, t, *J* = 7.6 Hz, Ar-H), 7.91 (2 H, d, *J* = 7.8 Hz, Ar-H), 8.07 ( 1H, s, Ar-H), 8.14 (2 H, d, *J* = 8.5 Hz, Ar-H), 8.19 (2 H, d, *J* = 8.9 Hz, Ar-H), 8.26 (1H, d, *J* = 8.4 Hz, Ar-H), 8.33 (2 H, d, *J* = 8.9 Hz, Ar-H), 8.45 (1H, s, Ar-H), 8.58 (2 H, d, *J* = 8.5 Hz, Ar-H), 12.86 (1H, s, NH); ^13^C NMR (100 MHz, DMSO-d6) δ (ppm): 36.8, 44.0, 47.3, 113.3, 118.1, 120.6, 124.8, 125.7, 125.8, 127.0, 128.1, 128.7, 128.8, 130.4, 131.4, 132.6, 134.5, 140.7, 142.2, 142.9, 147.0, 147.3, 148.4, 151.5, 152.4, 154.5, 159.0, 167.2; Anal. calcd. for C_32_H_25_N_7_O_5_S_3_: C, 56.21; H, 3.69; N, 14.34; S, 14.07; Found: C, 56.43; H, 3.85; N, 14.42; S, 14.31.

##### 2-((4-Allyl-5-(2-(4-(methylsulfonyl)phenyl)quinolin-4-yl)-4*H*-1,2,4-triazol-3-yl)thio)-*N*-(4-(*p*-tolyl)thiazol-2-yl)acetamide (8o)

White solid (76% yield); R_f_ = 0.29; m.p: 204–206 °C; ^1^H NMR (400 MHz, DMSO-*d*_*6*_) δ (ppm): 2.34 (3 H, s, CH_3_), 3.33 (3 H, s, SO_2_CH_3_), 4.41 (2 H, s, SC*H*_*2*_), 4.66 (2 H, d, *J* = 3.9 Hz, NC*H*_*2*_), 4.84 (1H, d, *J*_*trans*_ = 17.2 Hz, N-CH_2_CH = C*H*_*2*_), 5.11 (1H, d, *J*_*cis*_ = 10.5 Hz, N-CH_2_CH = C*H*_*2*_), 5.76–5.92 (1H, m, C*H* = CH_2_), 7.26 (2 H, d, *J* = 8.0 Hz, Ar-H), 7.62 (1H, s, Ar-H), 7.66–7.73 (1H, m, Ar-H), 7.82 (2 H, d, *J* = 8.1 Hz, Ar-H), 7.92 (2 H, t, *J* = 8.9 Hz, Ar-H), 8.14 (2 H, d, *J* = 8.4 Hz, Ar-H), 8.26 (1H, d, *J* = 8.4 Hz, Ar-H), 8.45 (1H, s, Ar-H), 8.58 (2 H, t, *J* = 8.1 Hz, Ar-*H*), 12.72 (1H, s, N*H*); ^13^C NMR (100 MHz, DMSO-*d*_*6*_) δ (ppm): 21.3, 36.7, 44.0, 47.3, 108.0, 118.1, 120.6, 125.7, 125.8, 126.1, 128.1, 128.7, 128.8, 129.8, 130.4, 131.4, 132.0, 132.6, 134.5, 137.7, 142.2, 142.9, 148.4, 149.6, 151.5, 152.4, 154.5, 158.1, 166.8; Anal. calcd. for C_33_H_28_N_6_O_3_S_3_: C, 60.72; H, 4.32; N, 12.87; S, 14.73; Found: C, 61.01; H, 4.45; N, 13.09; S, 14.68.

### Biology

#### Cell viability assay

The viability of **8a-o** was tested on the normal human mammary gland epithelial (MCF-10 A) cell line. After incubating MCF-10 A cells for four days with 50 µM of each tested compound [51, 52]. See Appendix A for more details.

#### Antiproliferative assay

The MTT assay was used to determine the antiproliferative activity of **8a-o** against four human cancer cell lines, with Erlotinib serving as a control [53, 54]. Appendix A has more information.

#### EGFR inhibitory assay

The EGFR-TK assay investigated the inhibitory activity of the most potent antiproliferative derivatives (**8f**, **8k**, and **8 m**) against EGFR [55, 56]. For more details, see Appendix A.

#### BRAF^V600E^ inhibitory assay

The most potent derivatives, **8f**, **8k**, and **8 m**, with highest antiproliferative traits, were tested for their potential to inhibit BRAF^V600E^ using vemurafenib as a reference [41, 42]. Refer to Appendix A for more details.

#### HER-2 inhibitory assay

The HER-2 kinase assay was used to assess the inhibitory activity of compounds **8f**, **8k**, and **8 m** on HER-2 enzyme [38, 46]. Lapatinib was the reference chemical. See Appendix A for more details.

#### Apoptotic markers tests

Compounds **8f** and **8k** were tested against the A-549 lung cancer cell line as activators of caspases-3, 8, 9. Bax and p53 and as down-regulators of the anti-apoptotic protein Bcl-2 [57]. Appendix A contains more experimental details.

#### Immunomodulators evaluation

qRT-PCR test was used to assess the impact of the most potent compounds, **8f** and **8k**, on immunomodulatory protein levels (TNF-α and IL-6) [65]. Dexamethasone served as the reference drug. See Appendix A for more experimental details.

### Computational studies

Molecular docking simulations for EGFR (PDB ID: 1M17) [66], BRAF^V600E^ in complex with vemurafenib (PDB ID: 3OG7) [67], and HER-2 in complex with a known inhibitor (PDB ID: 3PP0) [25] were validated through a redocking test, in which the structures of the test proteins were maintained in a fixed state while the co-crystallized ligands. Refer to Appendix A for additional information.

## Supplementary Information

Below is the link to the electronic supplementary material.

Supplementary Material 1

Supplementary Material 2

## Data Availability

The authors declare that the data supporting the findings of this study are available within the supplementary materials.
