# Supplementary material for: Design and synthesis of novel thiazole/1,2,4-triazole/quinoline hybrids as antiproliferative agents, apoptosis inducers, immunomodulators, and multi-EGFR/BRAFV600E/HER-2 inhibitors
Source: Mol Divers. 2026 Jan 21;30(4):5791–816. doi: 10.1007/s11030-026-11467-9 (PMC13332894; doi:10.1007/s11030-026-11467-9)
Supplement: Supplementary file 2 — Supplementary Material 2 [file 11030_2026_11467_MOESM2_ESM.docx]

**Supporting Information**

**Design and synthesis of novel thiazole/1,2,4-triazole/quinoline hybrids as antiproliferative agents, apoptosis inducers, immunomodulators, and multi-EGFR/BRAF^V600E^/HER-2 inhibitors**

Aliaa M. Mohassab^1,2,#^, Bahaa G.M. Youssif^3,#^*, Abdullah Yahya Abdullah Alzahrani^4^, Hesham A. Abou-Zied^5^, Stefan Bräse^6^*, Mohamed A.A. Abdel-Aal^7^, Kamal S. Abdelrahman^7^, Samar H. Abbas^1,2*^

^1^Medicinal Chemistry Department, Faculty of Pharmacy, Minia University, Minia 61519, Egypt; ^2^Medicinal Chemistry Department, Faculty of Pharmacy, Minia National University, New Minia, Egypt; ^3^Pharmaceutical Organic Chemistry Department, Faculty of Pharmacy, Assiut University, Assiut 71526, Egypt; ^4^Department of Chemistry, Faculty of Science, King Khalid University, Abha 61413, Saudi Arabia; ^5^Medicinal Chemistry Department, Faculty of Pharmacy, Deraya University, Minia, Egypt; ^6^Institute of Biological and Chemical Systems, IBCS-FMS, Karlsruhe Institute of Technology, 76131 Karlsruhe, Germany; ^7^Department of Pharmaceutical Chemistry, Faculty of Pharmacy, Al-Azhar University, Assiut Branch, Assiut 71524, Egypt.

**To whom correspondence should be addressed:*

**Bahaa G. M. Youssif**, Ph.D. Pharmaceutical Organic Chemistry Department, Faculty of Pharmacy, Assiut University, Assiut 71526, Egypt.

Tel.: (002)-01098294419

E-mail address: [bgyoussif2@gmail.com](mailto:bgyoussif2@gmail.com)

**Stefan Bräse**

Institute of Biological and Chemical Systems, IBCS-FMS, Karlsruhe Institute of Technology, 76131 Karlsruhe, Germany. E-mail: [braese@kit.edu](mailto:braese@kit.edu)

^#^ Equally contributed

**Chemistry**

| **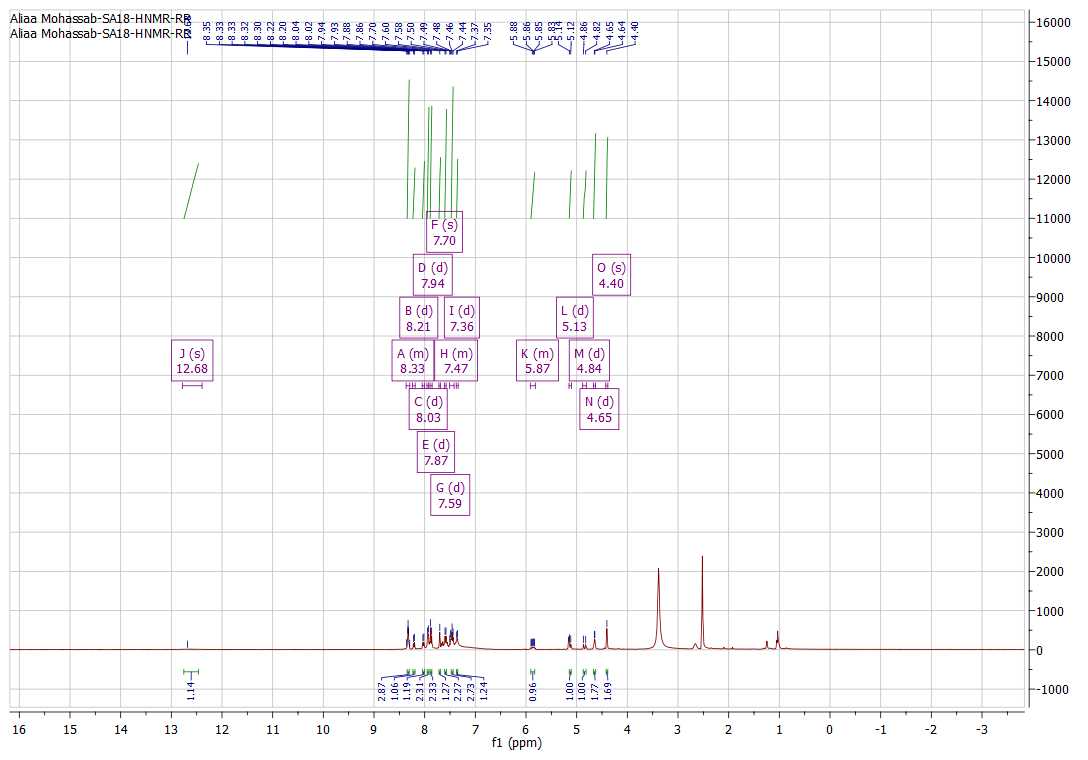** |
| --- |

**Figure S1: ^1^H NMR spectrum of compound 8a**

| **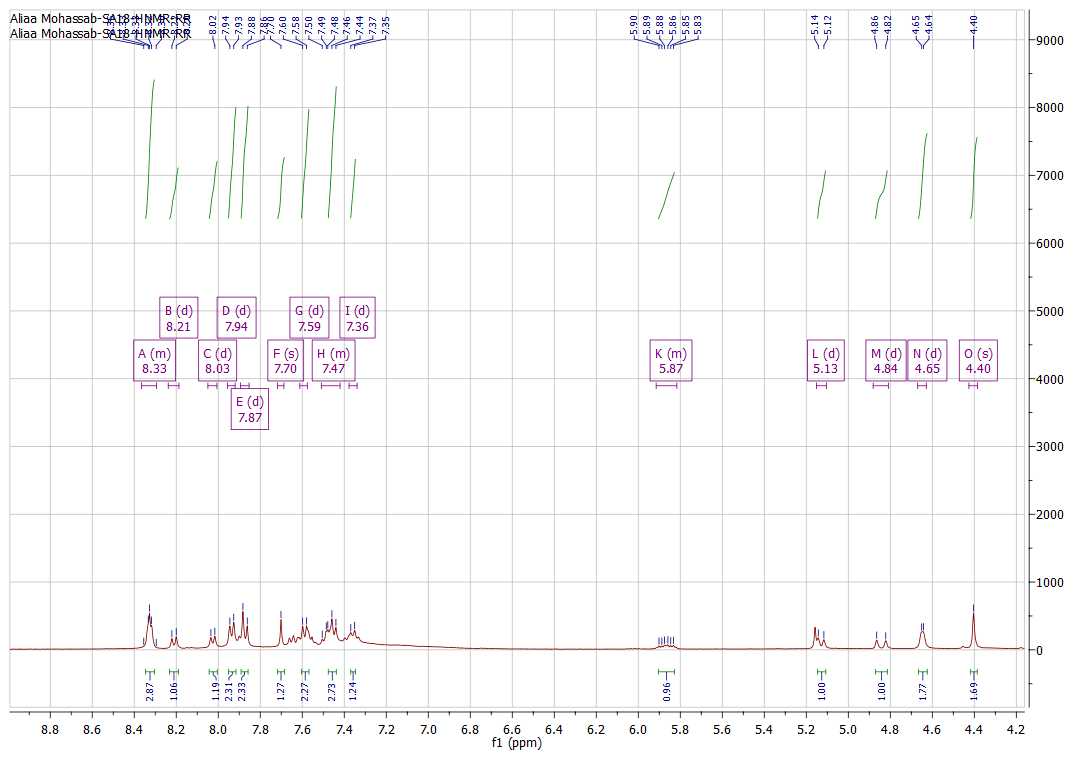** |
| --- |

**Figure S2: Expanded ^1^H NMR spectrum of compound 8a**

| 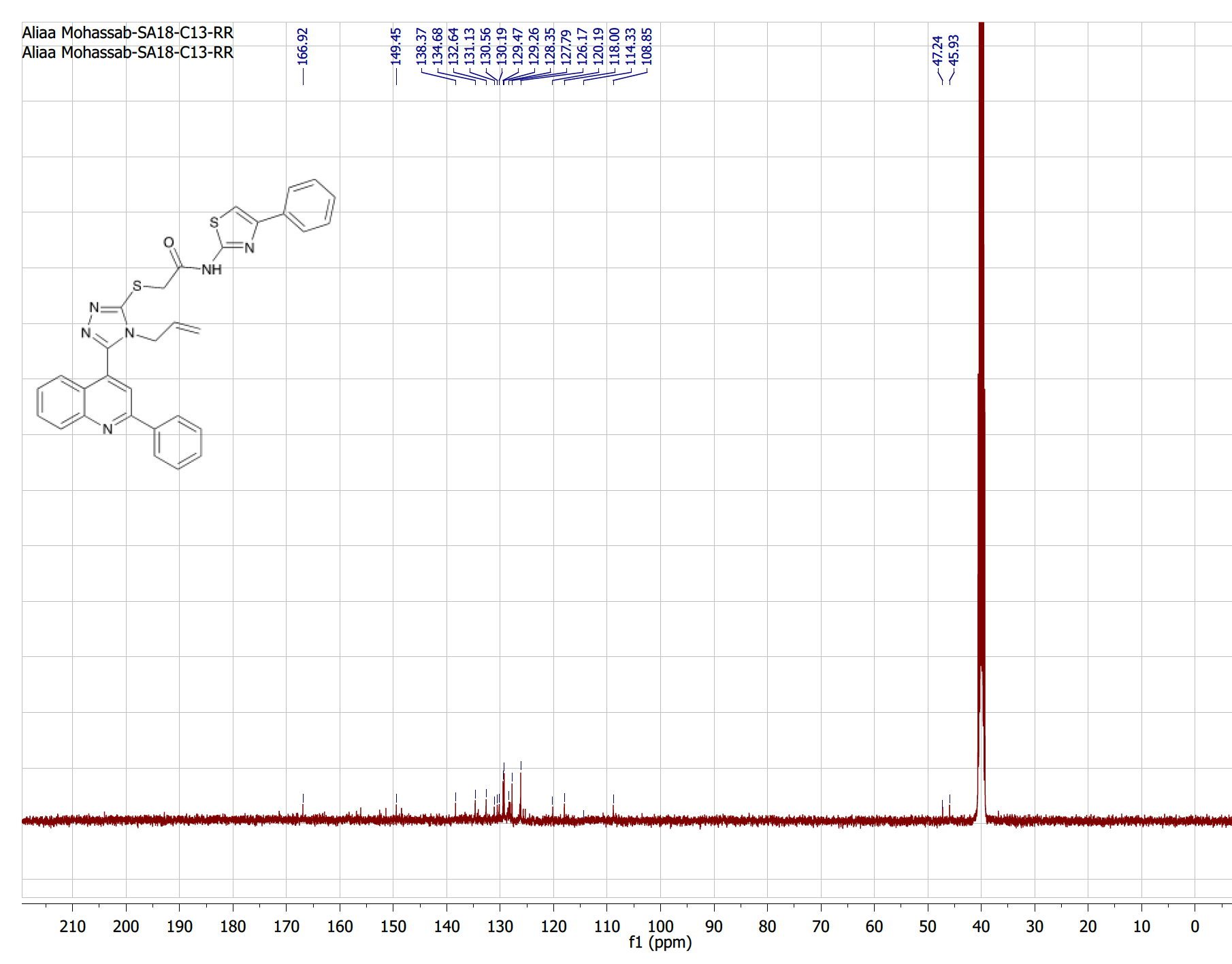 |
| --- |

**Figure S3: ^13^C NMR spectrum of compound 8a**

| **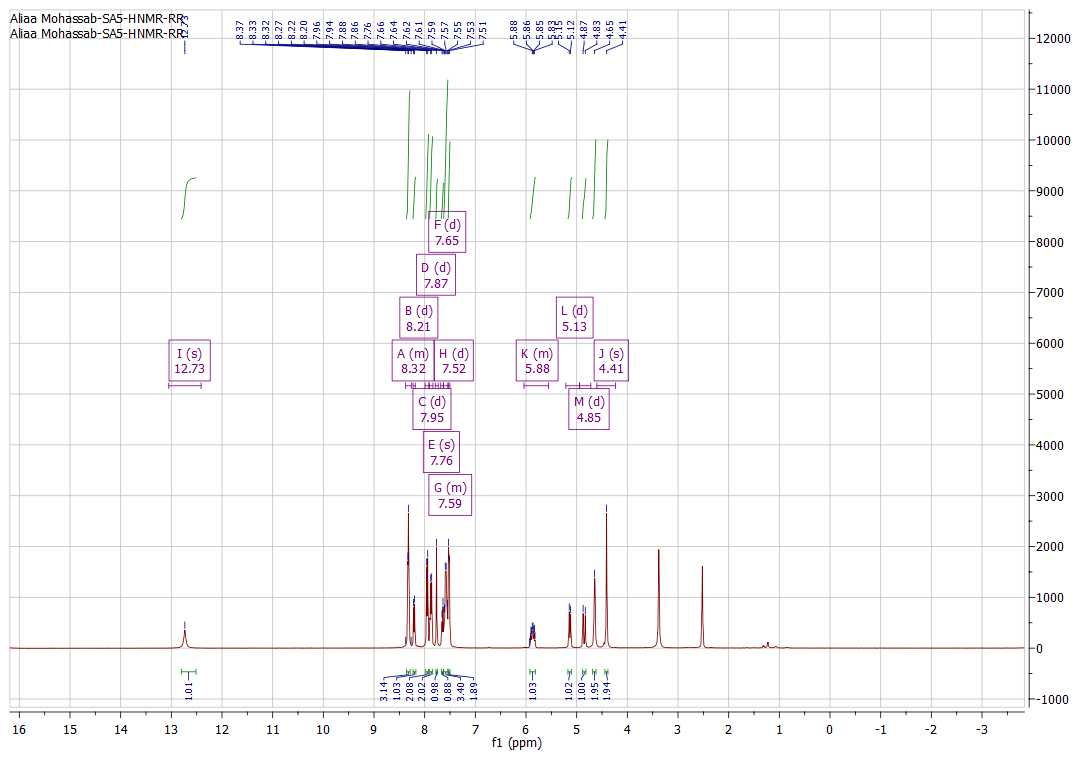** |
| --- |
| **Figure S4: ^1^H NMR spectrum of compound 8b** |
| **^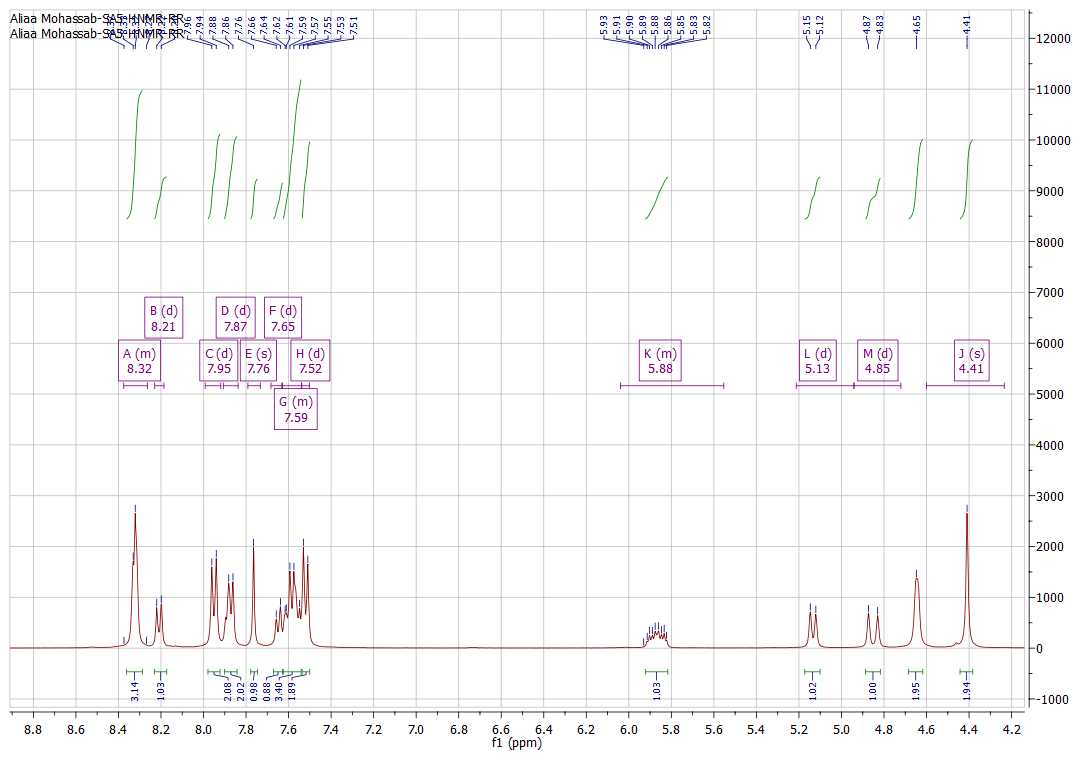^** |
| **Figure S5: Expanded ^1^H NMR spectrum of compound 8b** |
| **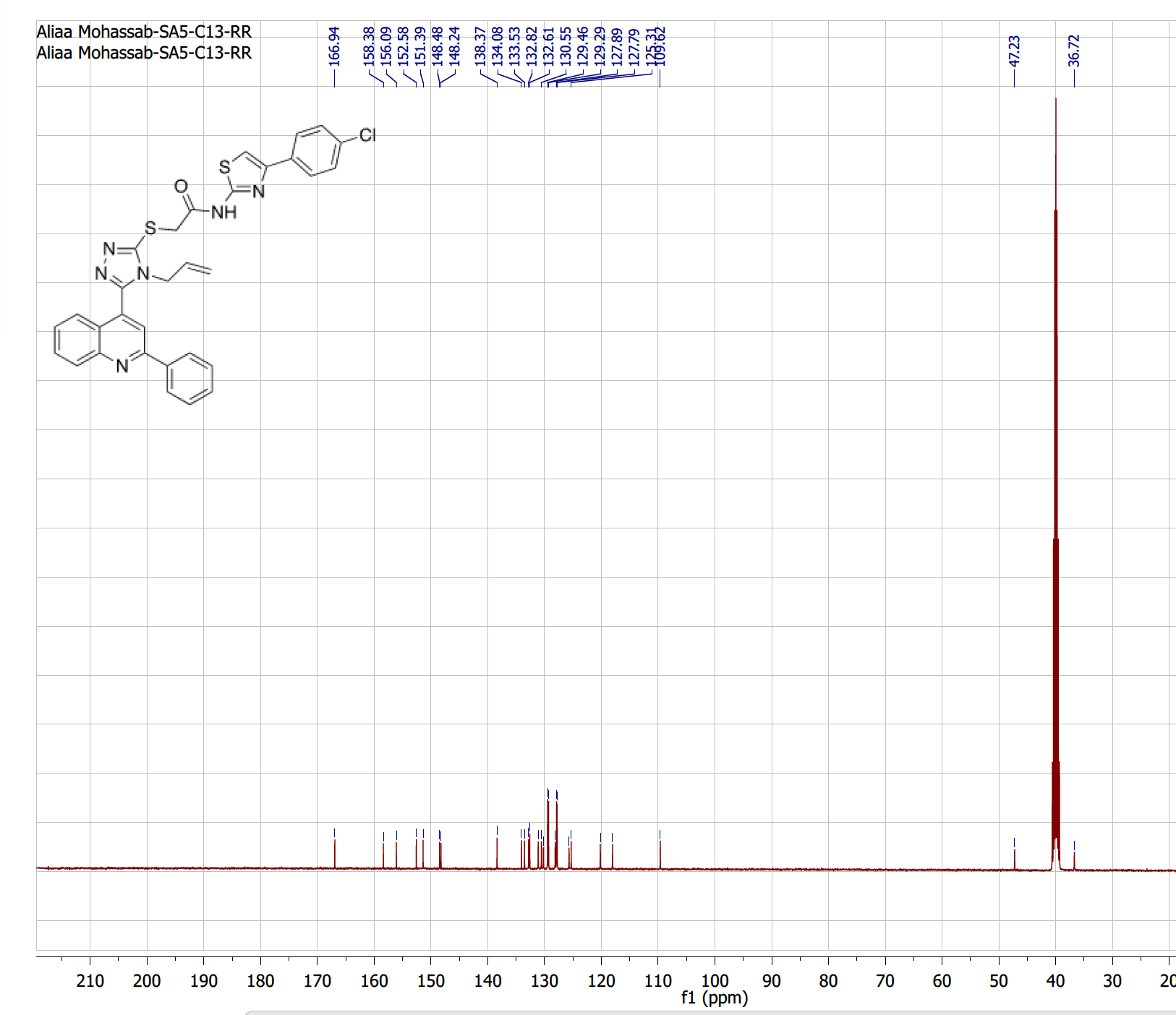** |
| **Figure S6: ^13^C NMR spectrum of compound 8b** |
| **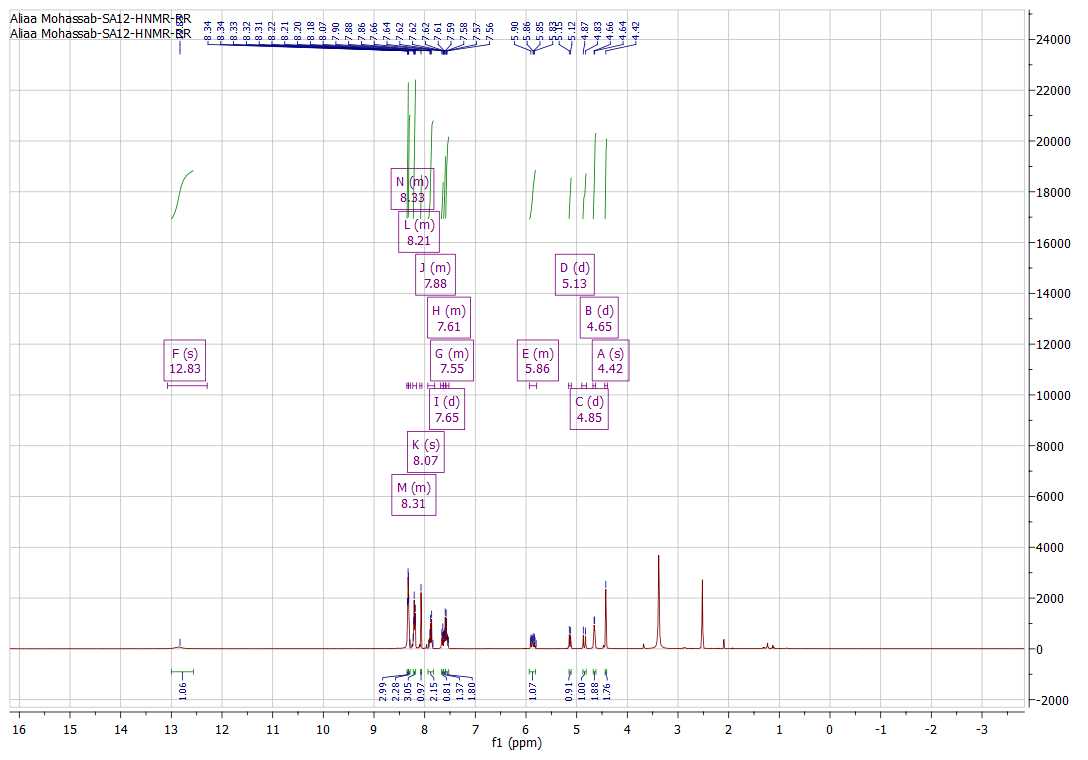** |
| **Figure S7: ^1^H NMR spectrum of compound 8c** |
| **^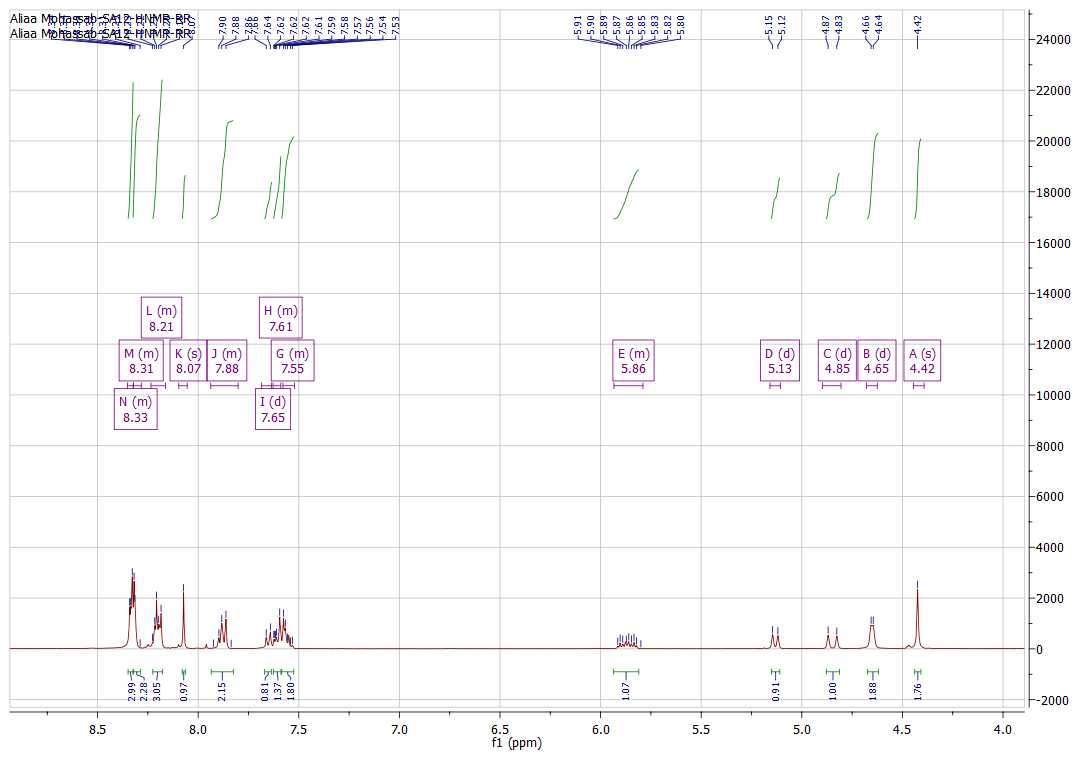^** |
| **Figure S8: Expanded ^1^H NMR spectrum of compound 8c** |
| **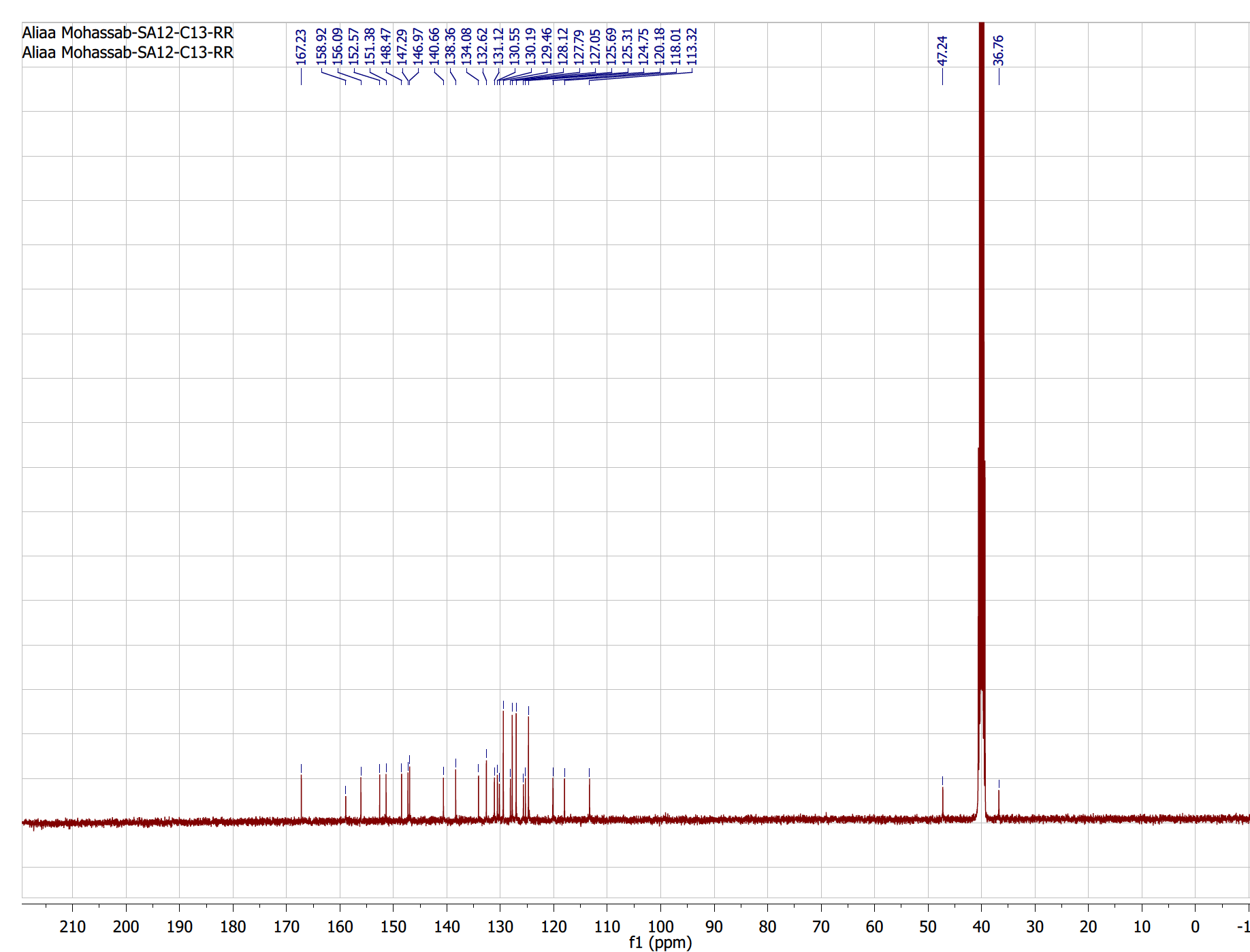** |
| **Figure S9: ^13^C NMR spectrum of compound 8c** |
| **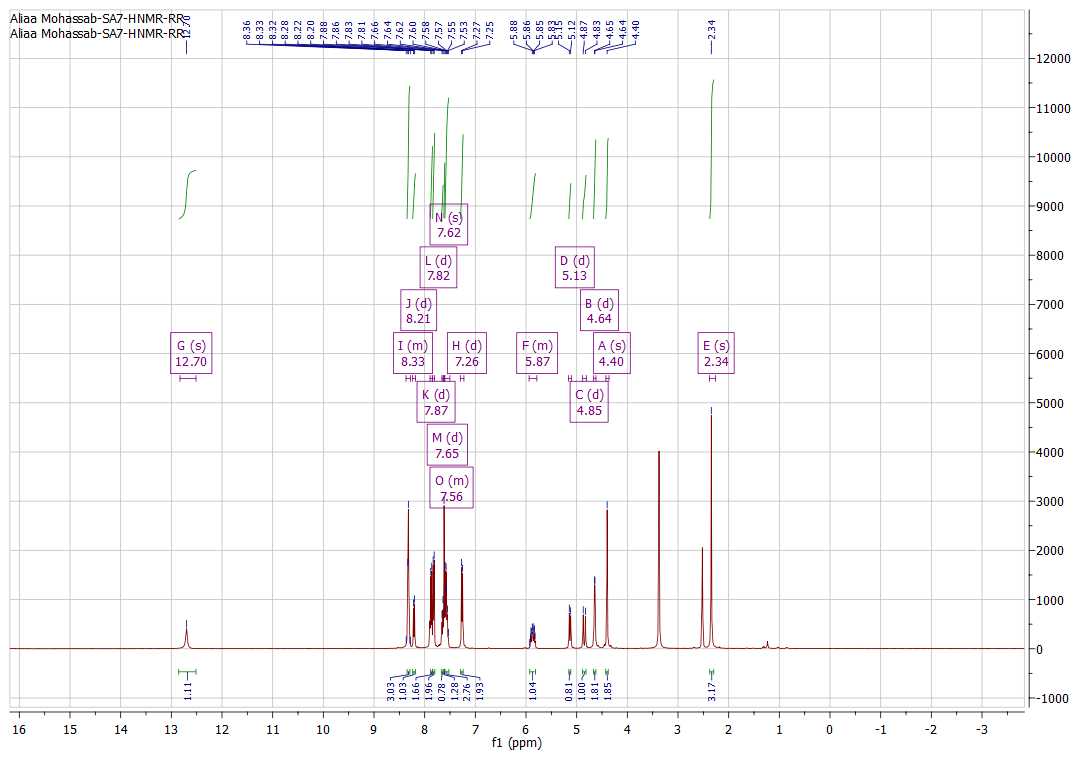** |
| **Figure S10: ^1^H NMR spectrum of compound 8d** |
| **^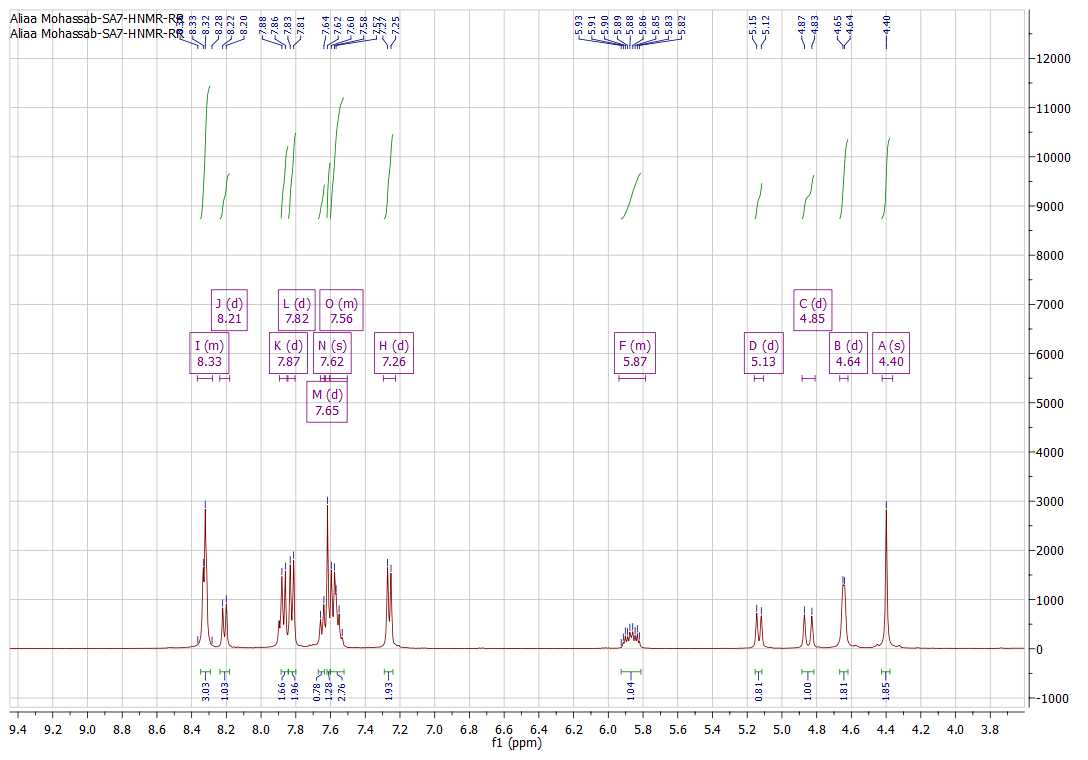^** |
| **Figure S11: Expanded ^1^H NMR spectrum of compound 8d** |
| **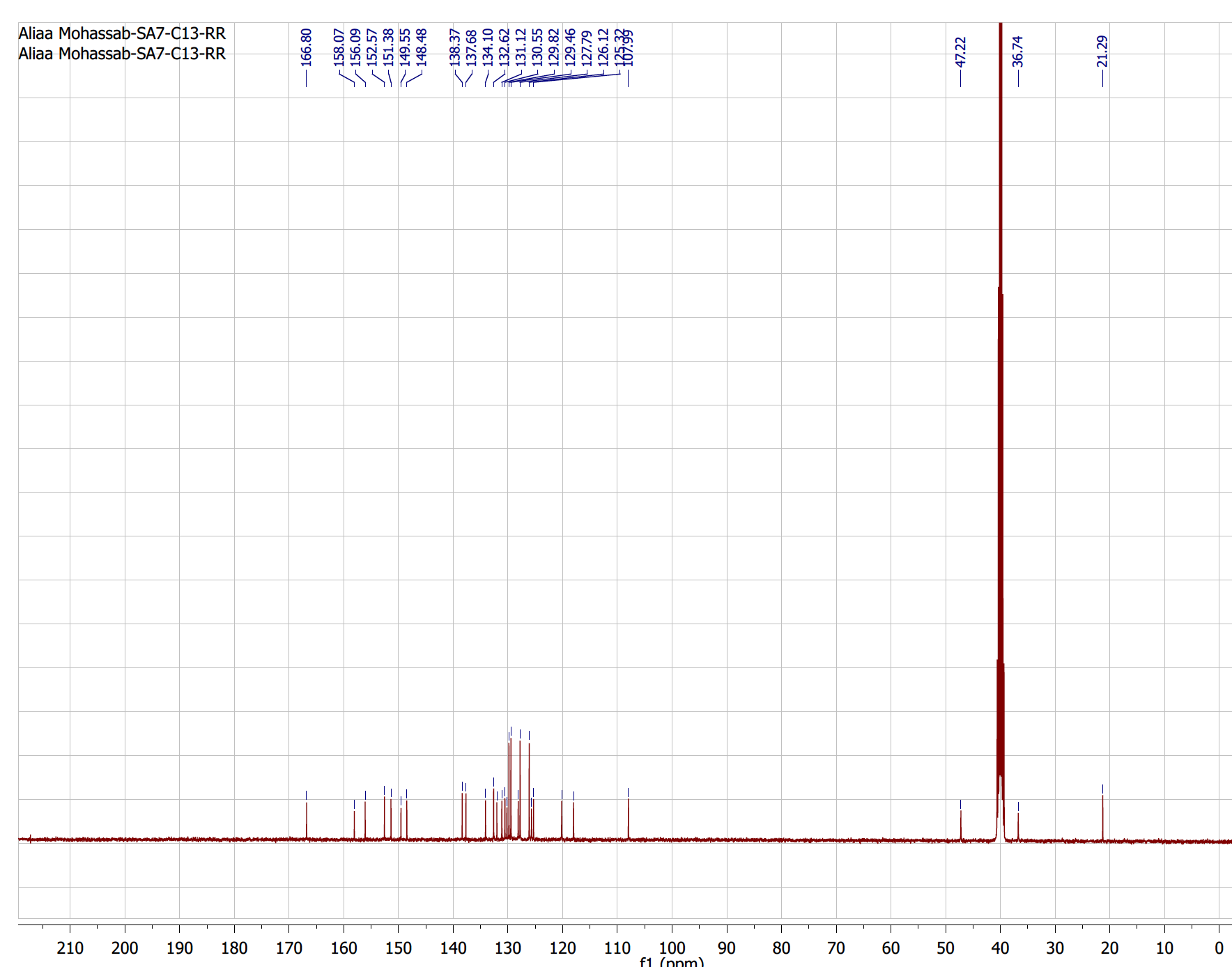** |
| **Figure S12: ^13^C NMR spectrum of compound 8d** |
| **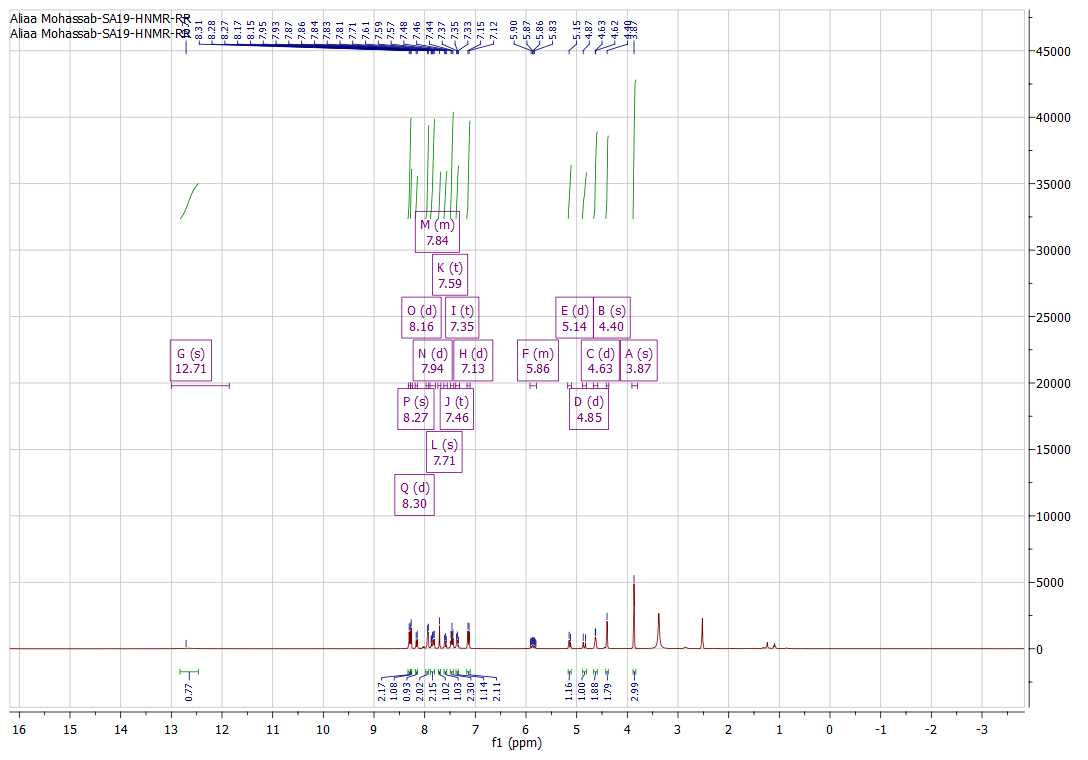** |
| **Figure S13: ^1^H NMR spectrum of compound 8e** |
| **^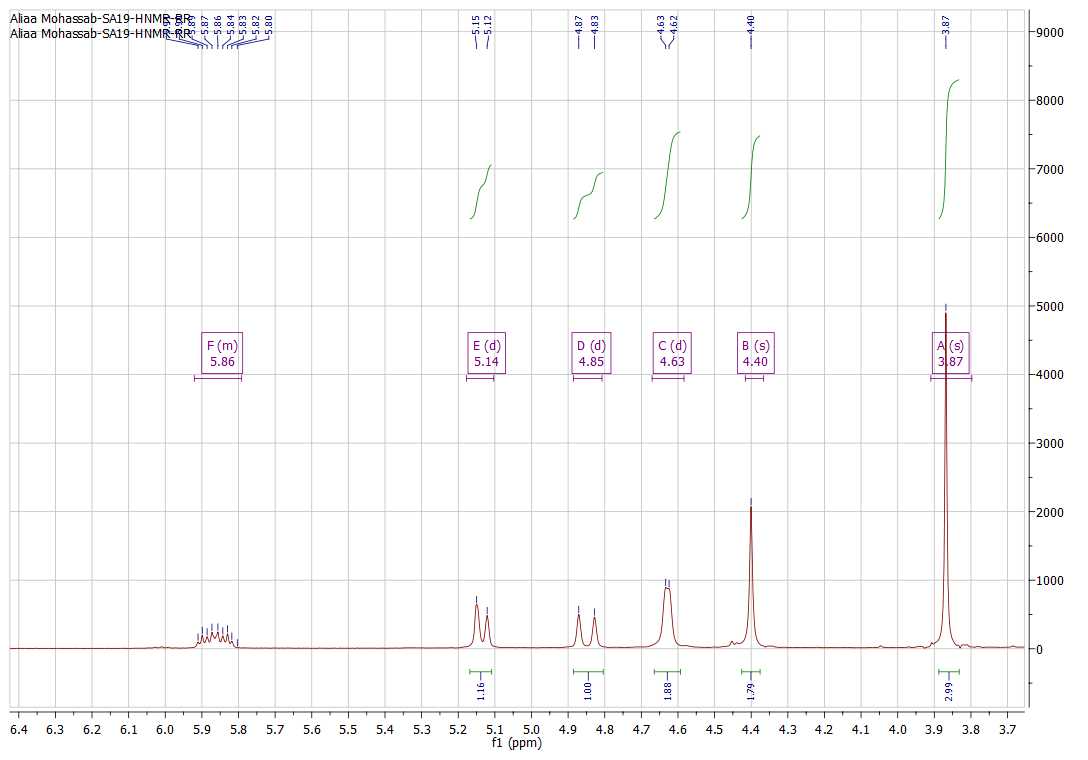^** |
| **Figure S14: Expanded (aliphatic) ^1^H NMR spectrum of compound 8e** |
| **^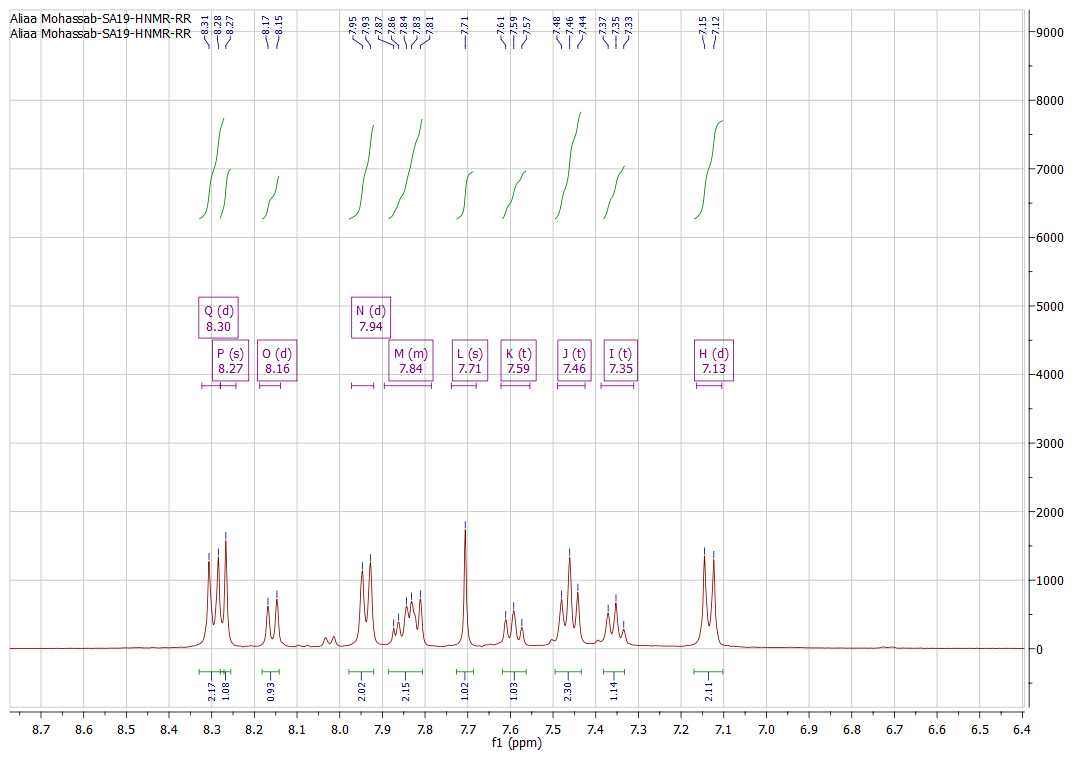^** |
| **Figure S15: Expanded (aromatic) ^1^H NMR spectrum of compound 8e** |
| **^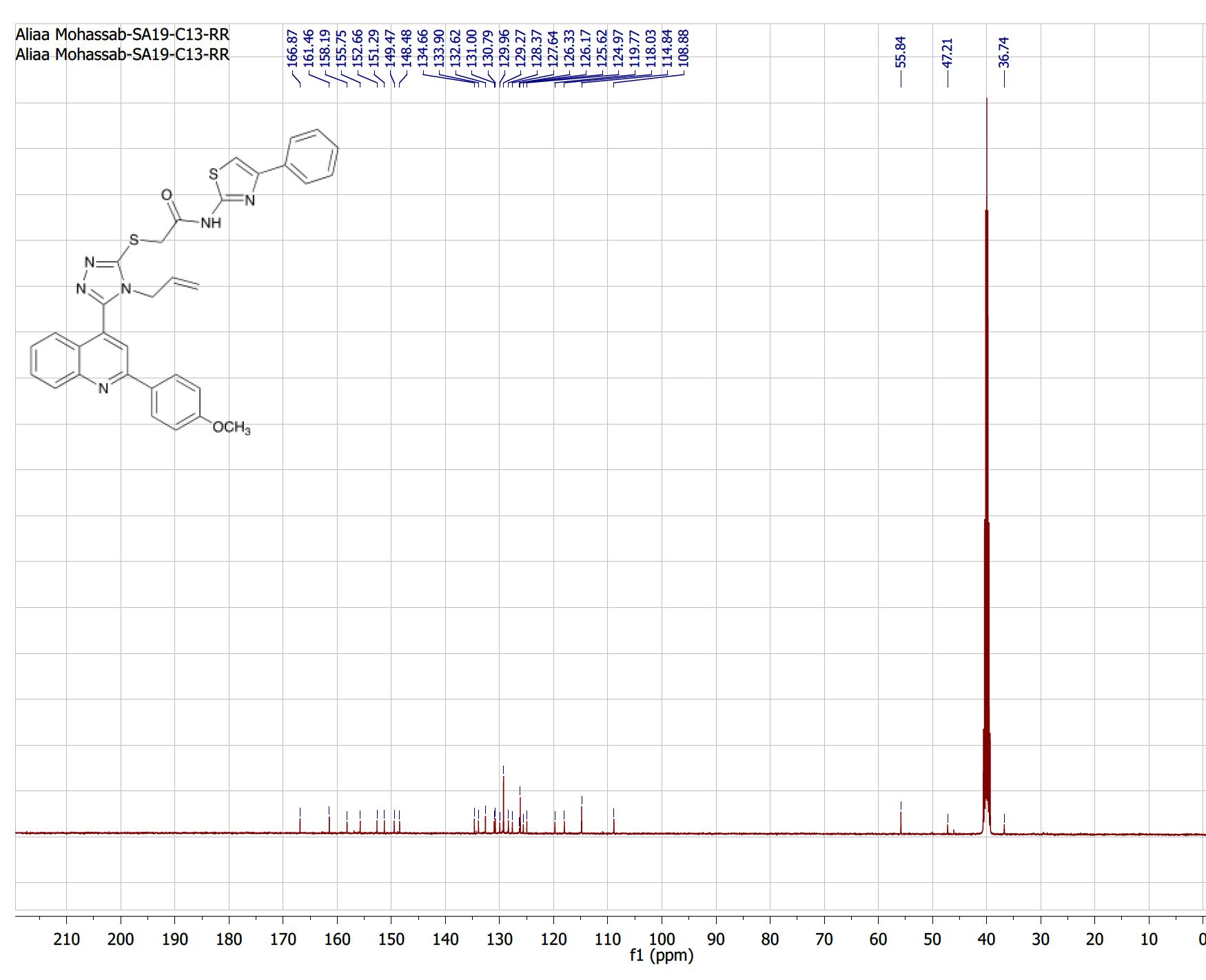^** |
| **Figure S16: ^13^C NMR spectrum of compound 8e** |
| **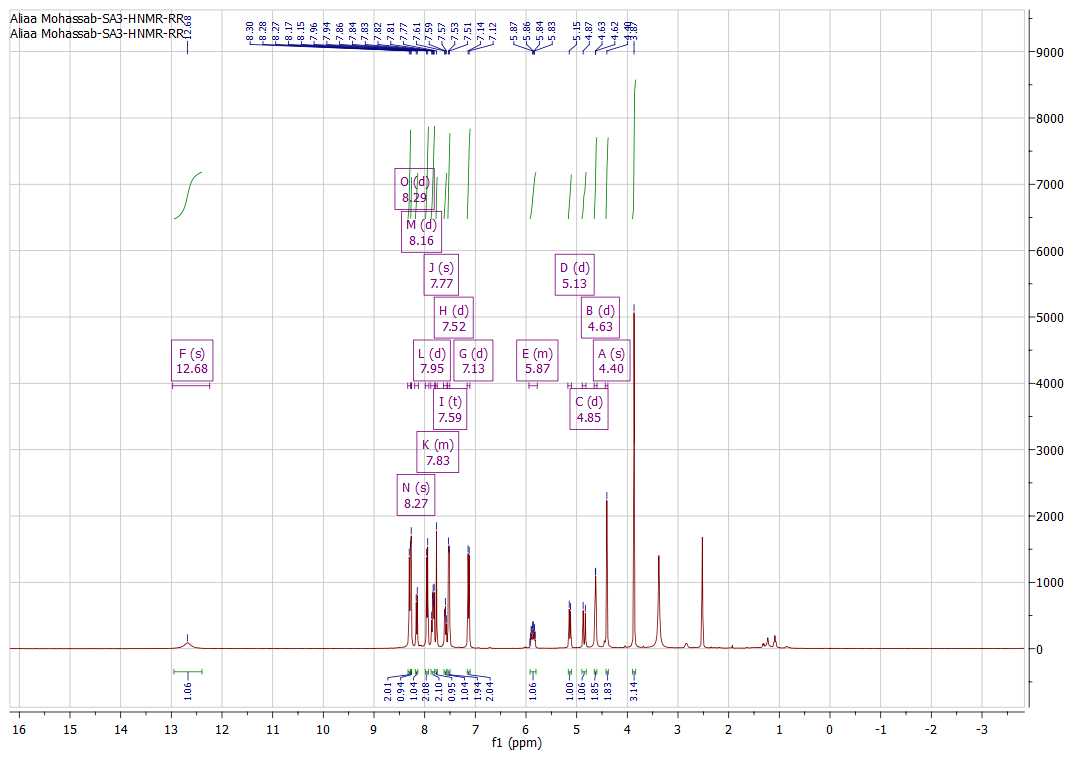** |
| **Figure S17: ^1^H NMR spectrum of compound 8f** |
| **^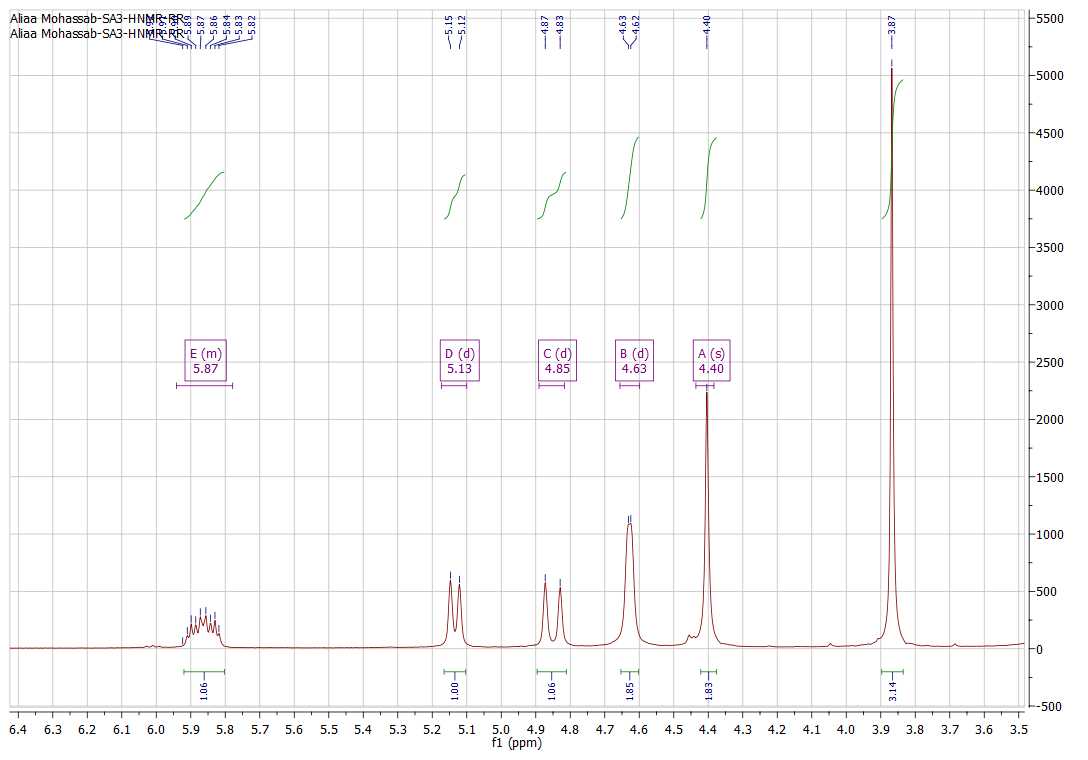^** |
| **Figure S18: Expanded (aliphatic) ^1^H NMR spectrum of compound 8f** |
| **^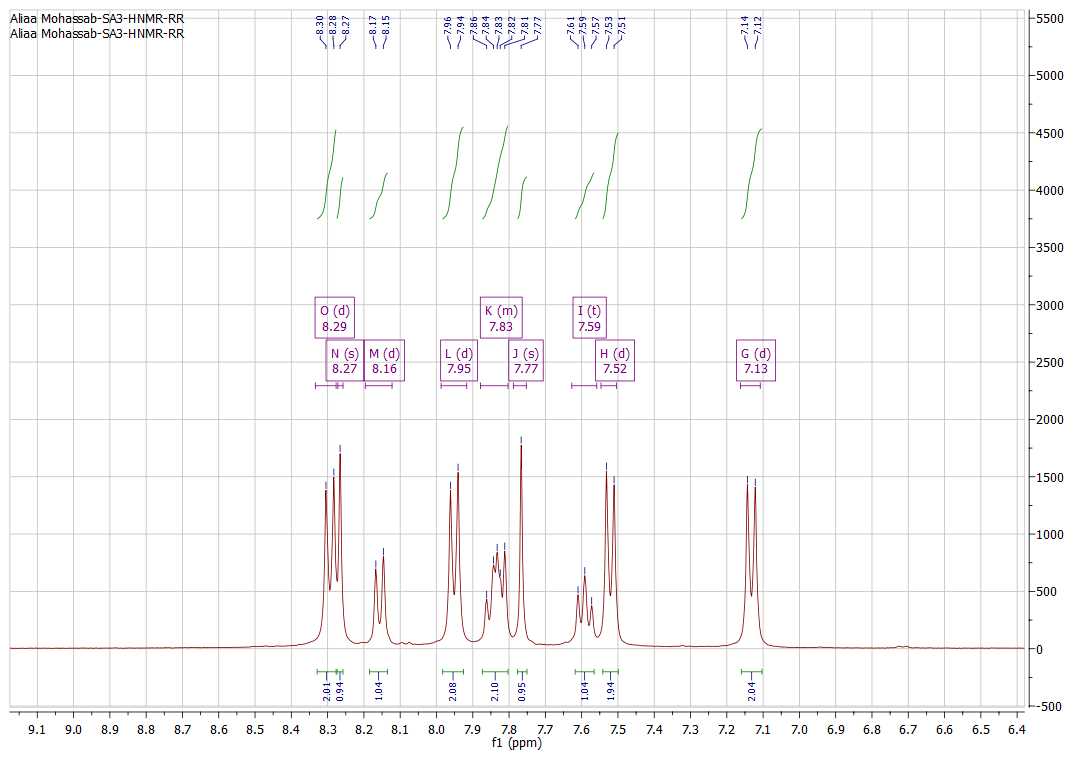^** |
| **Figure S19: Expanded (aromatic) ^1^H NMR spectrum of compound 8f** |
| **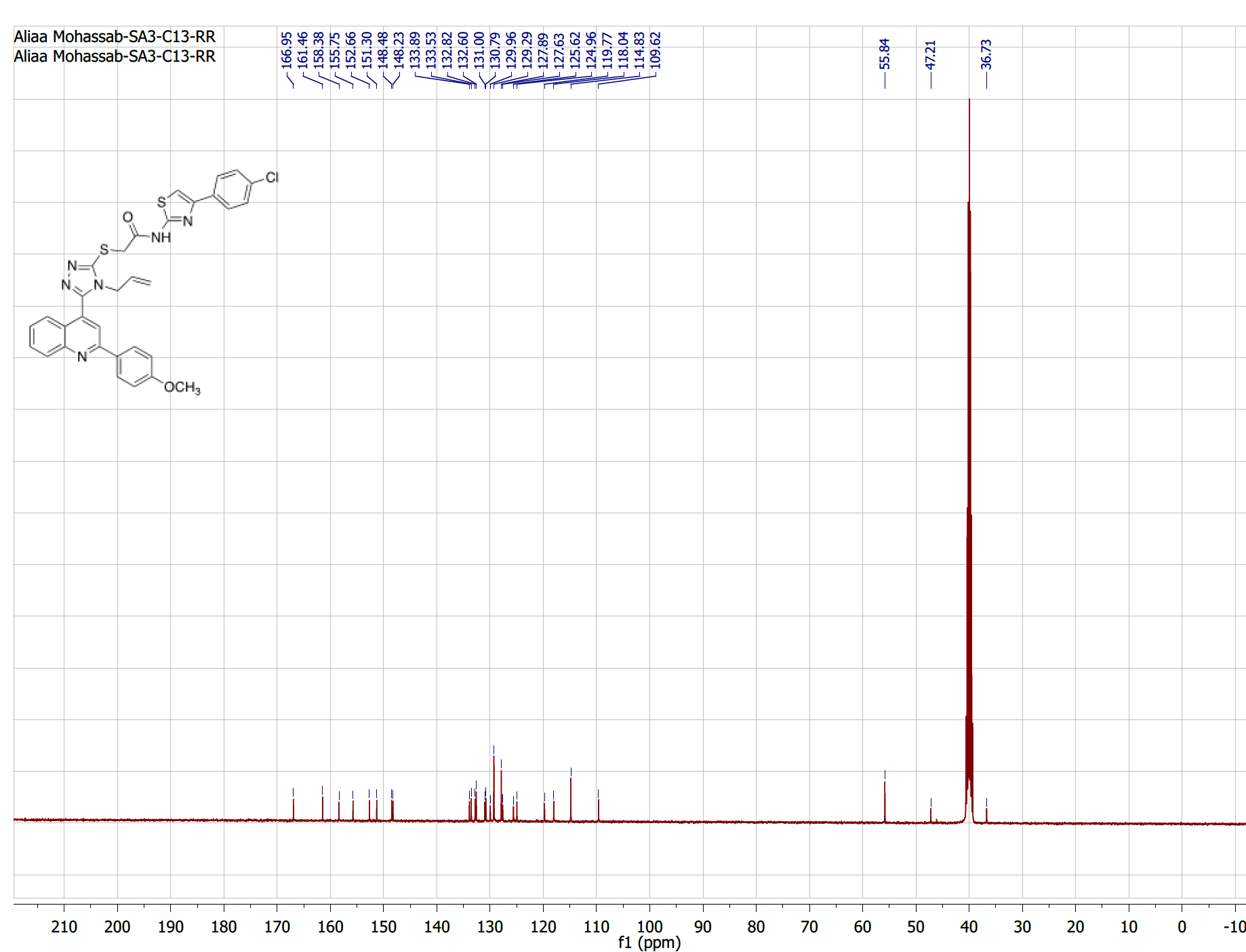** |
| **Figure S20: ^13^C NMR spectrum of compound 8f** |

| **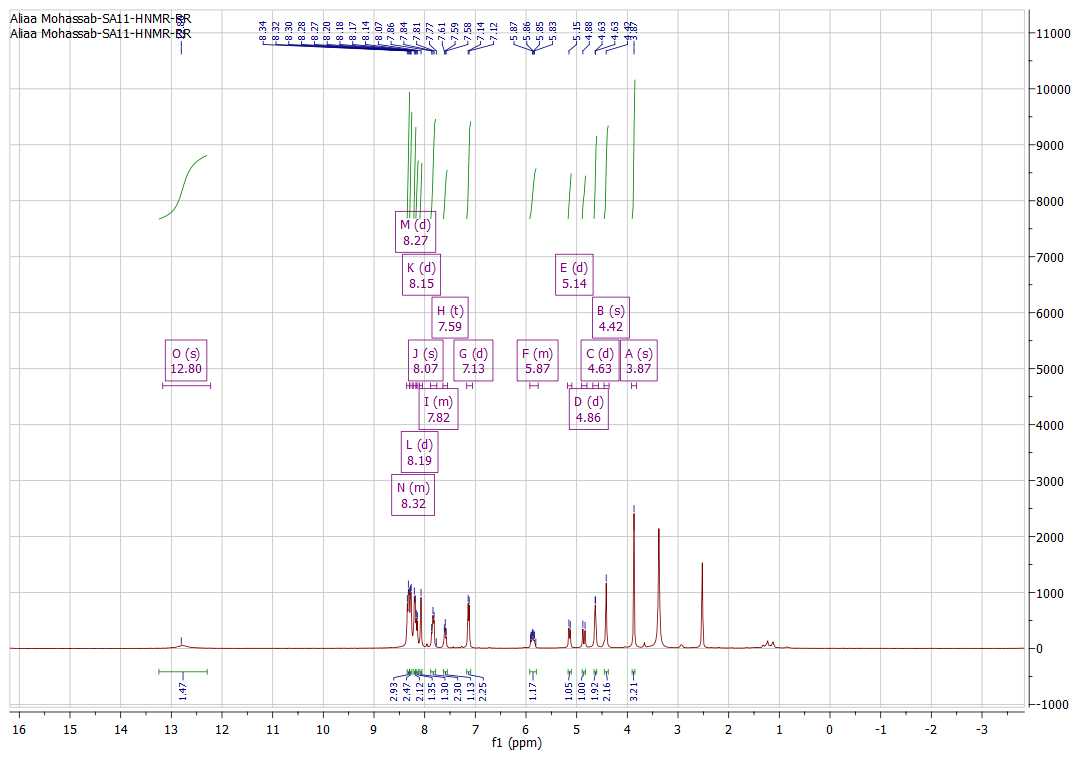** |
| --- |
| **Figure S21: ^1^H NMR spectrum of compound 8g** |
| **^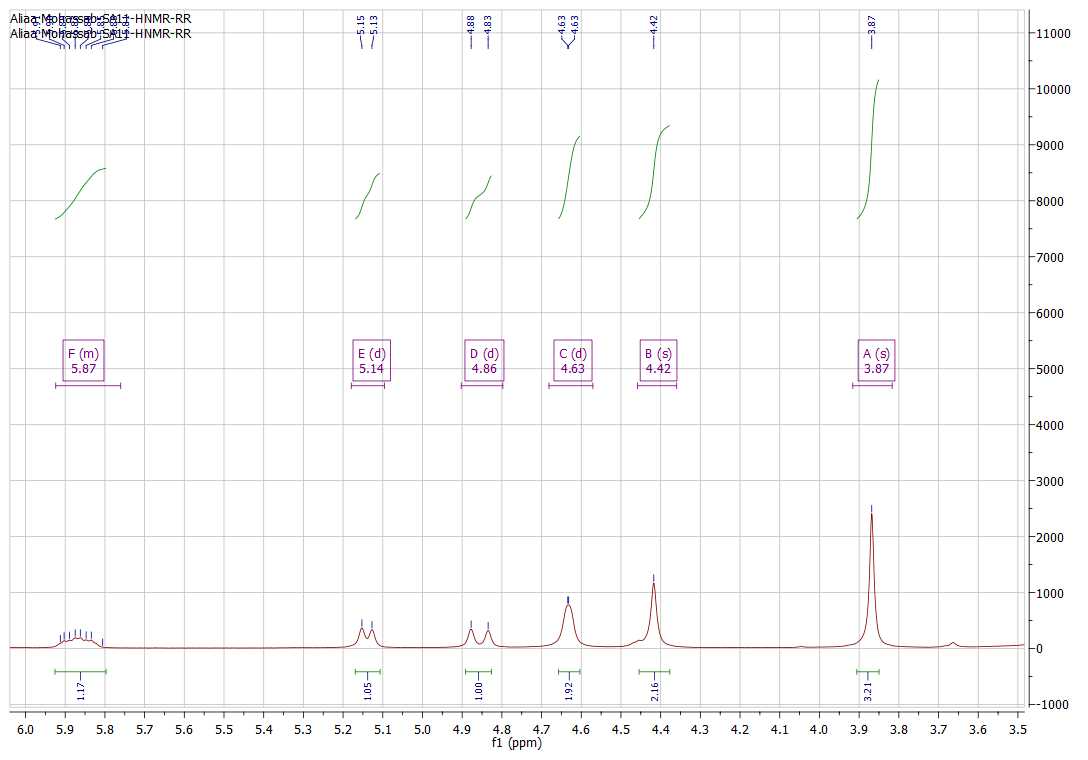^** |
| **Figure S22: Expanded (aliphatic) ^1^H NMR spectrum of compound 8g** |
| **^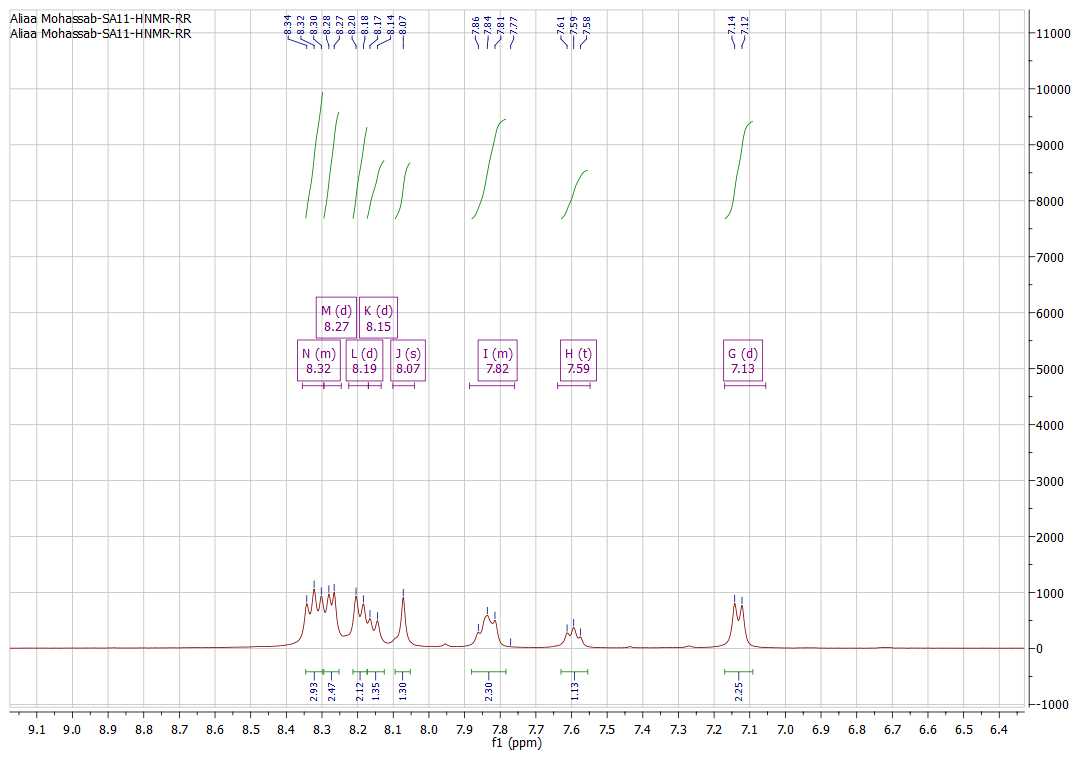^** |
| **Figure S23: Expanded (aromatic) ^1^H NMR spectrum of compound 8g** |
| **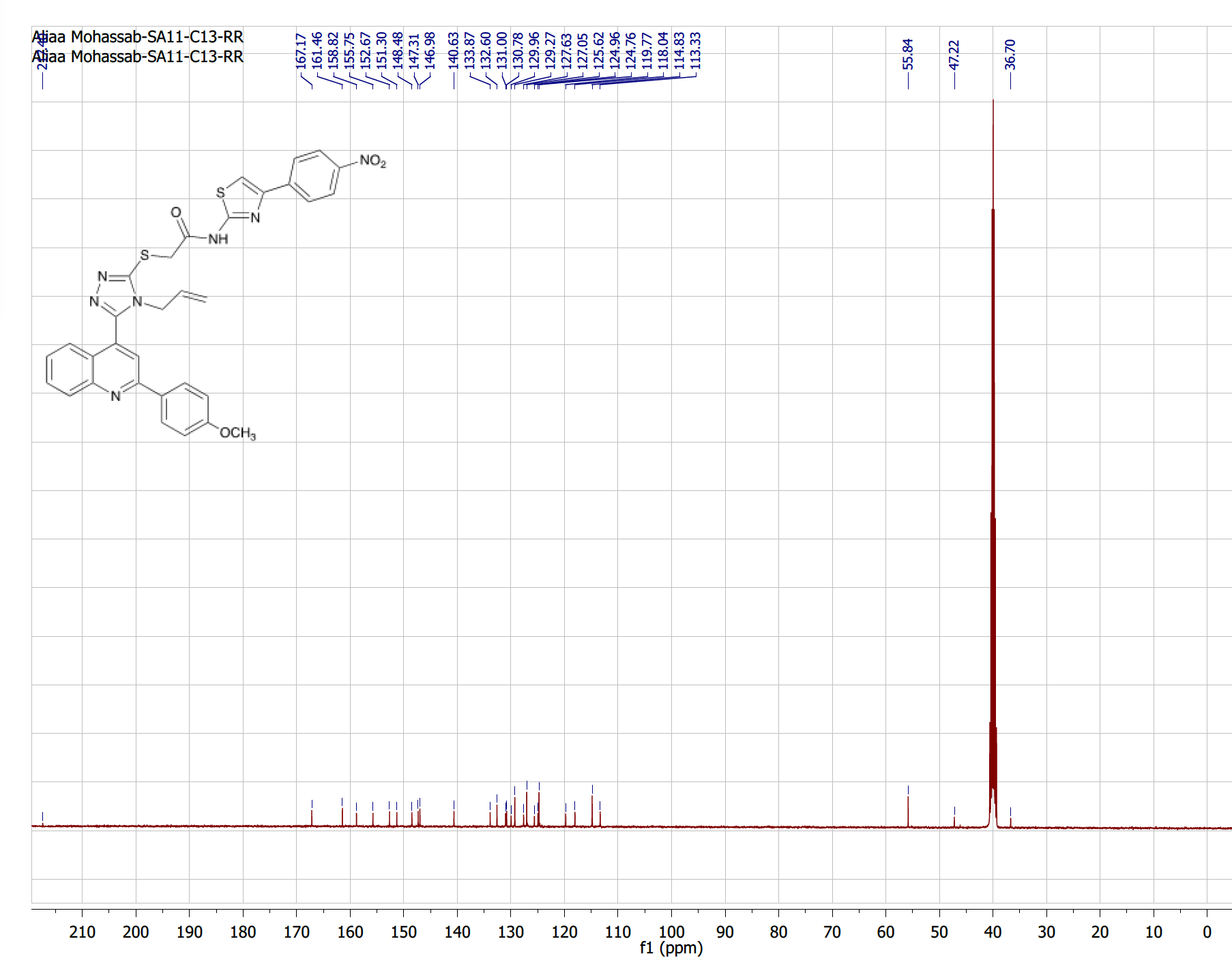** |
| **Figure S24: ^13^C NMR spectrum of compound 8g** |
| **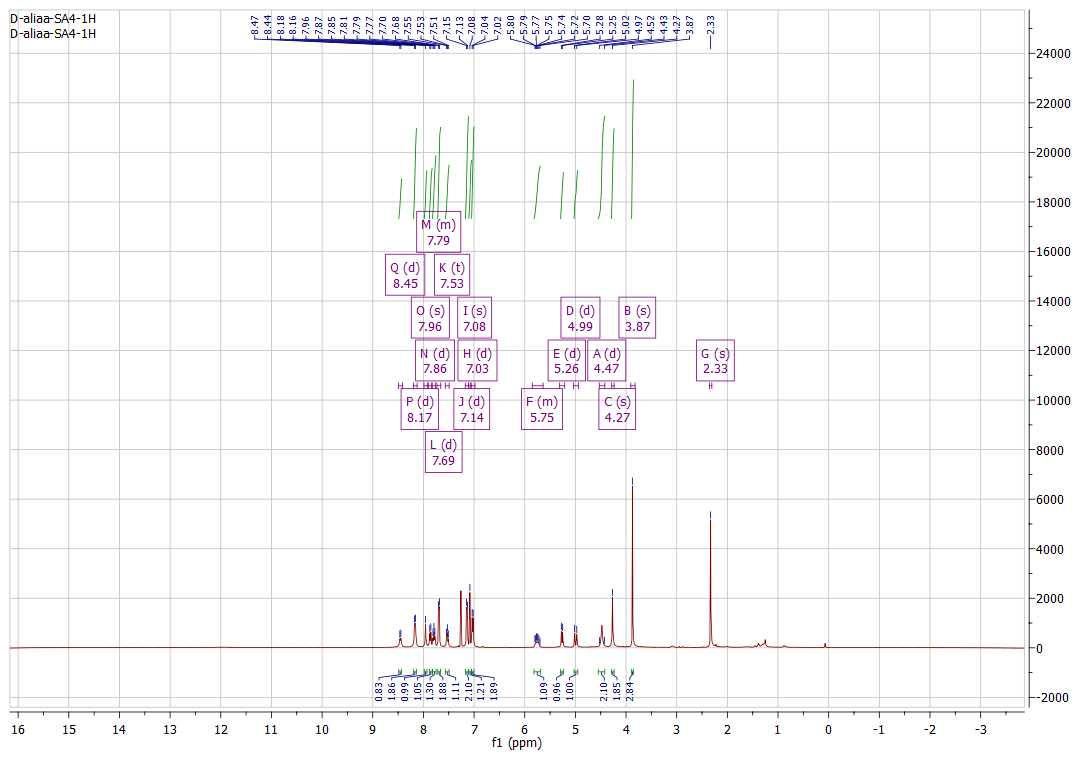** |
| **Figure S25: ^1^H NMR spectrum of compound 8h** |
| **^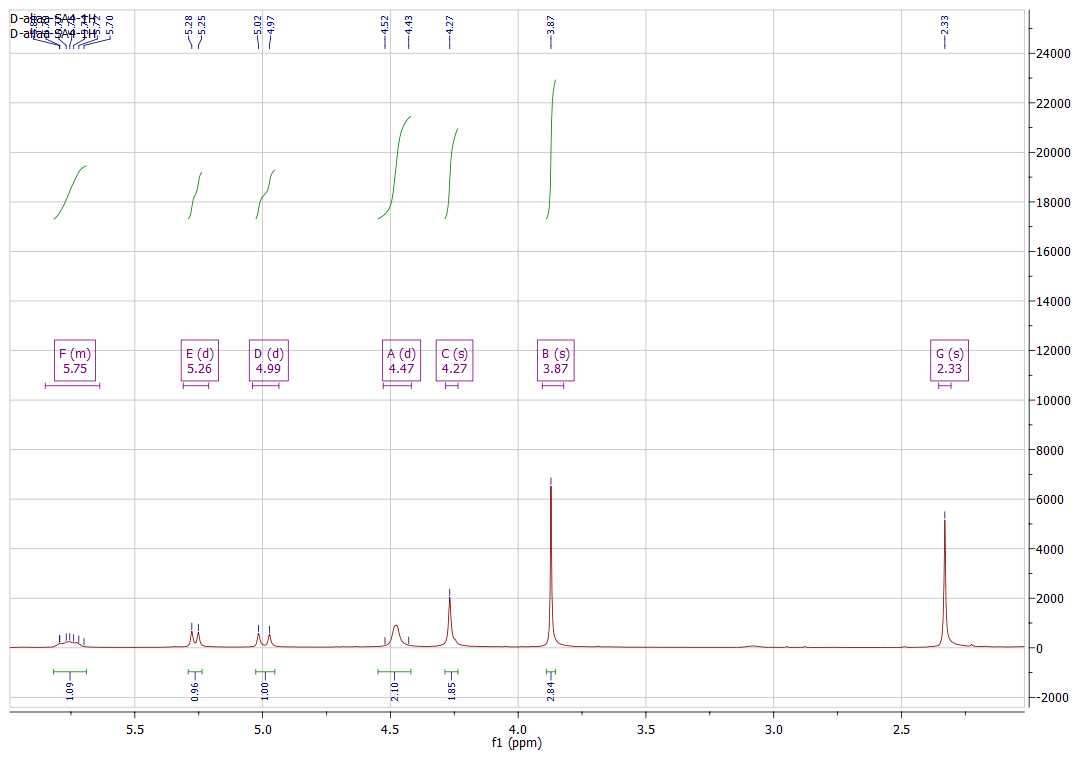^** |
| **Figure S26: Expanded (aliphatic) ^1^H NMR spectrum of compound 8h** |
| **^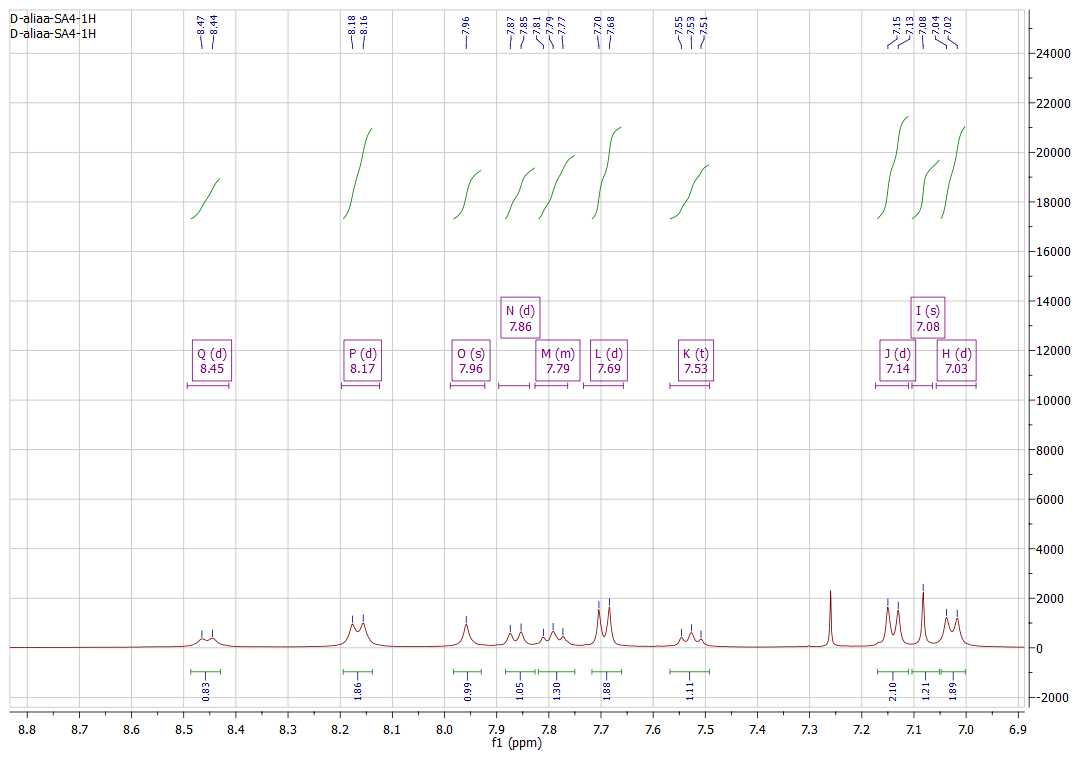^** |
| **Figure S27: Expanded (aromatic) ^1^H NMR spectrum of compound 8h** |
| **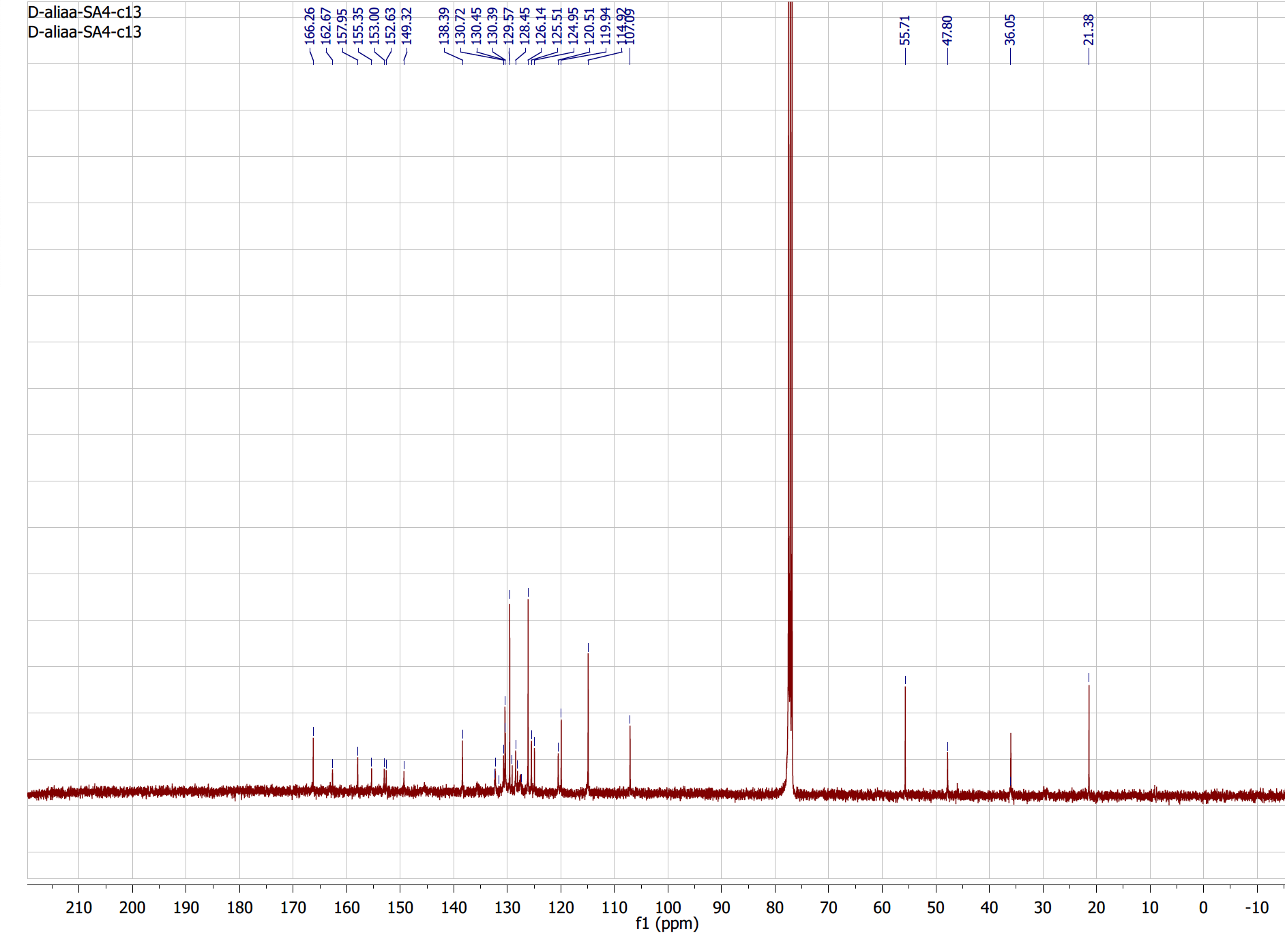** |
| **Figure S28: ^13^C NMR spectrum of compound 8h** |

| **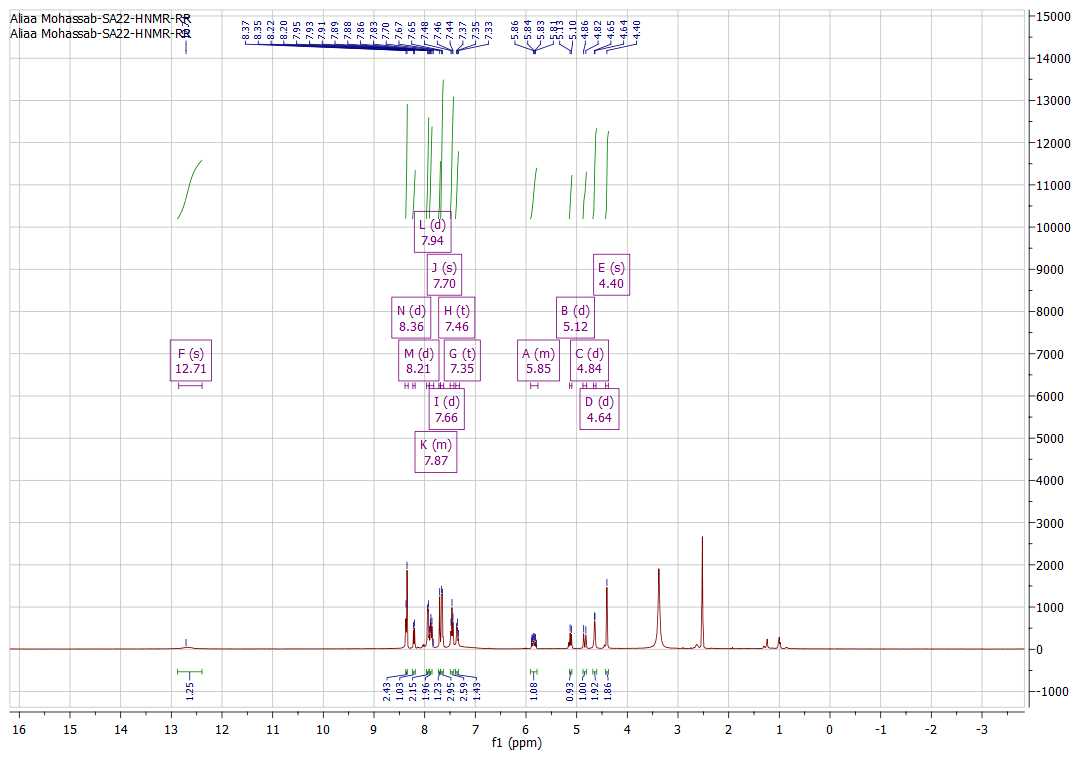** |
| --- |
| **Figure S29: ^1^H NMR spectrum of compound 8i** |
| **^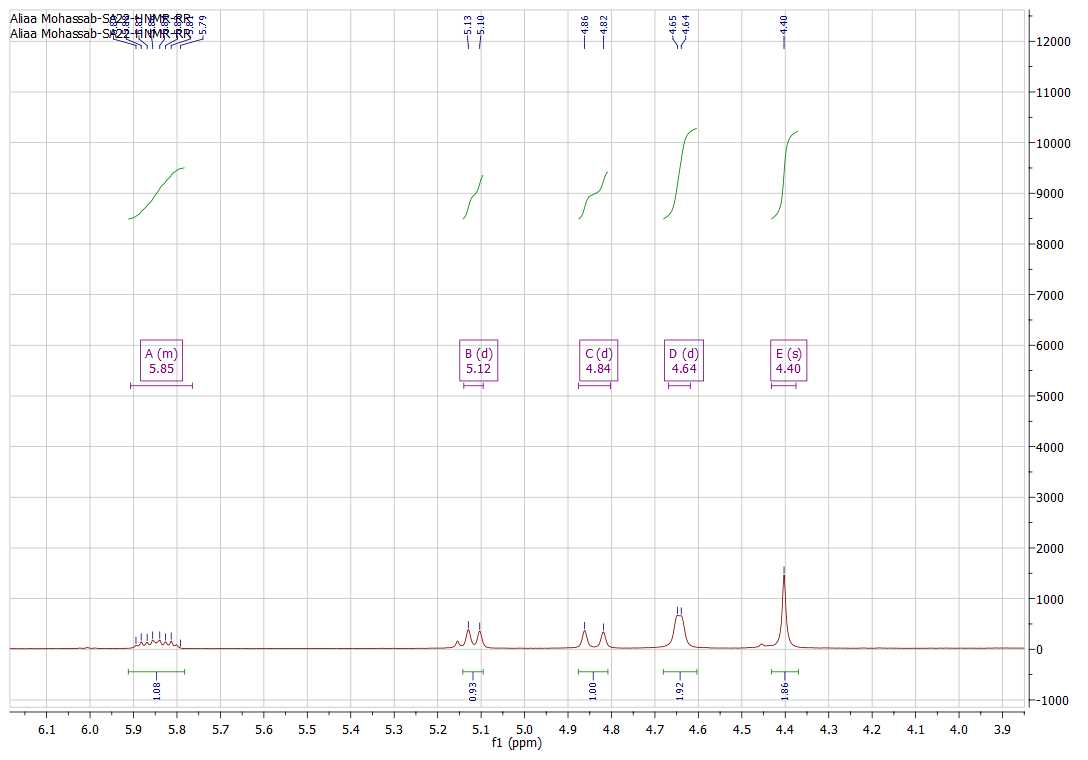^** |
| **Figure S30: Expanded (aliphatic) ^1^H NMR spectrum of compound 8i** |
| **^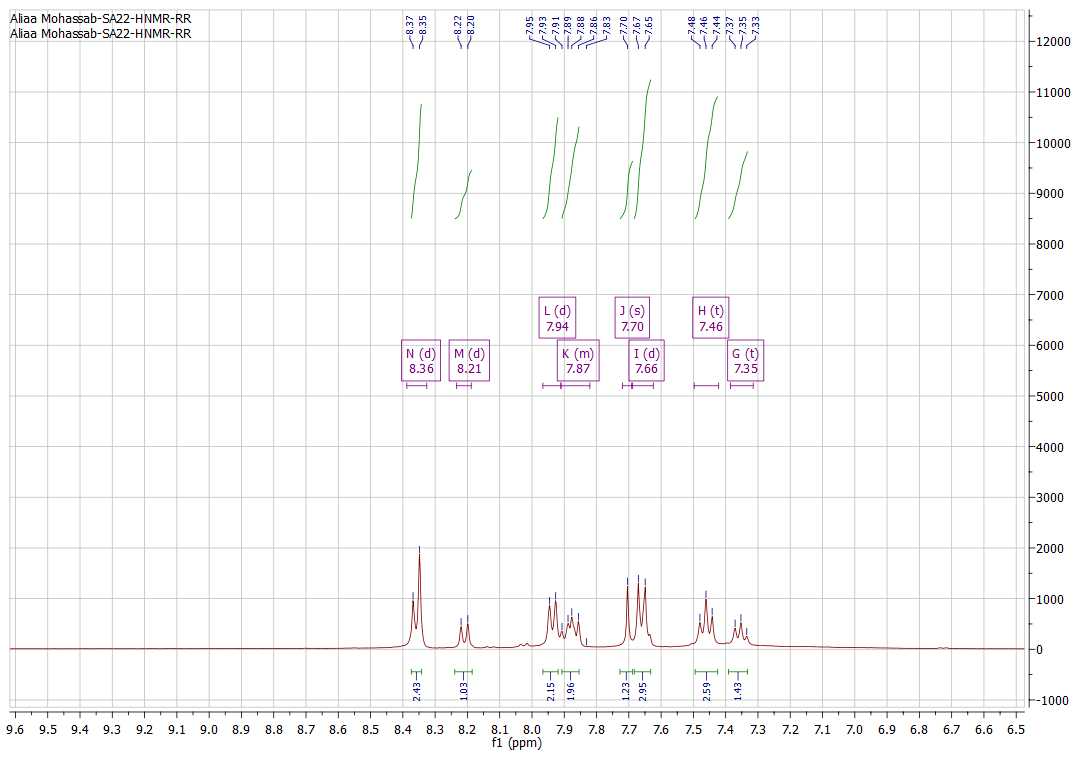^** |
| **Figure S31: Expanded (aromatic) ^1^H NMR spectrum of compound 8i** |
| **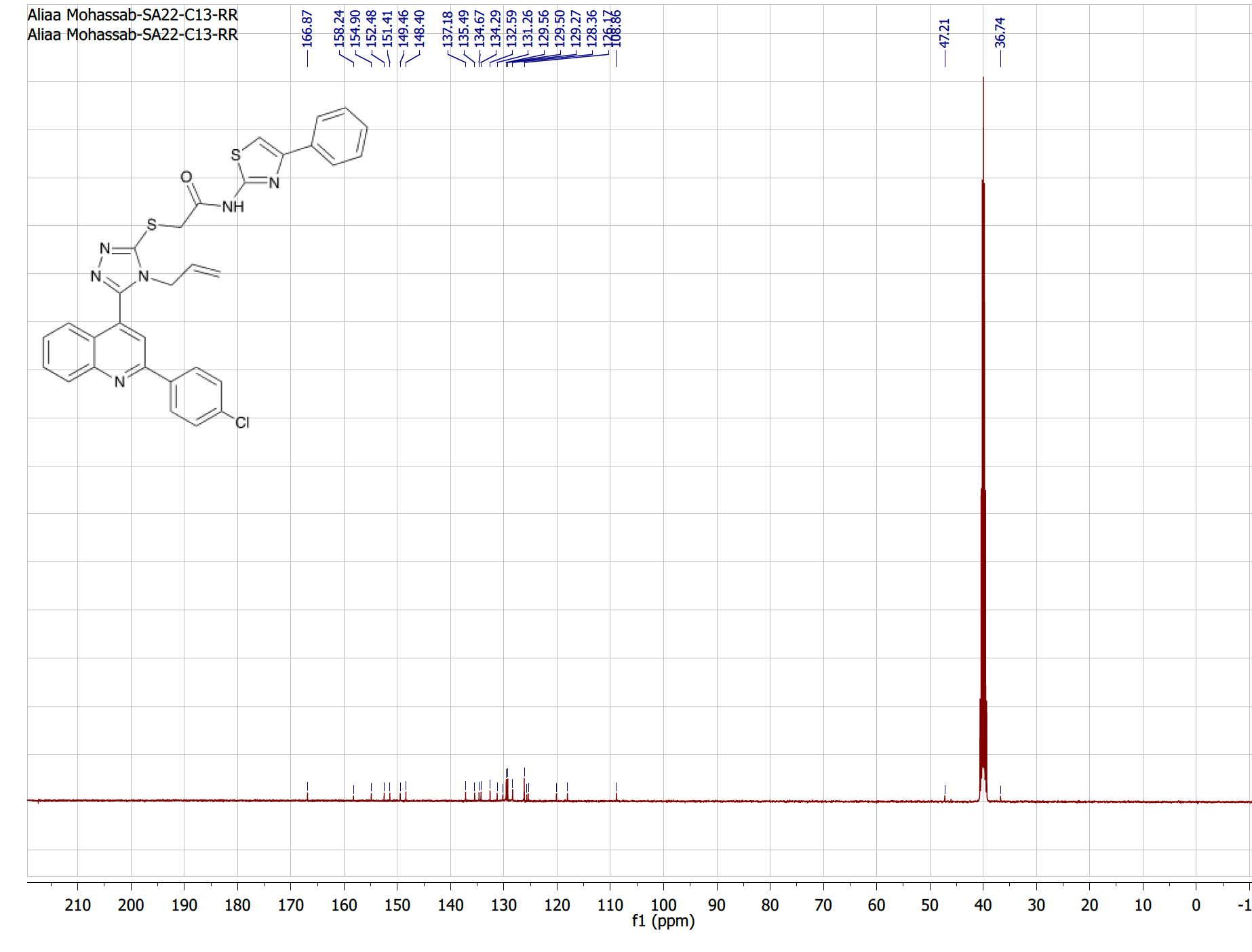** |
| **Figure S32: ^13^C NMR spectrum of compound 8i** |

| **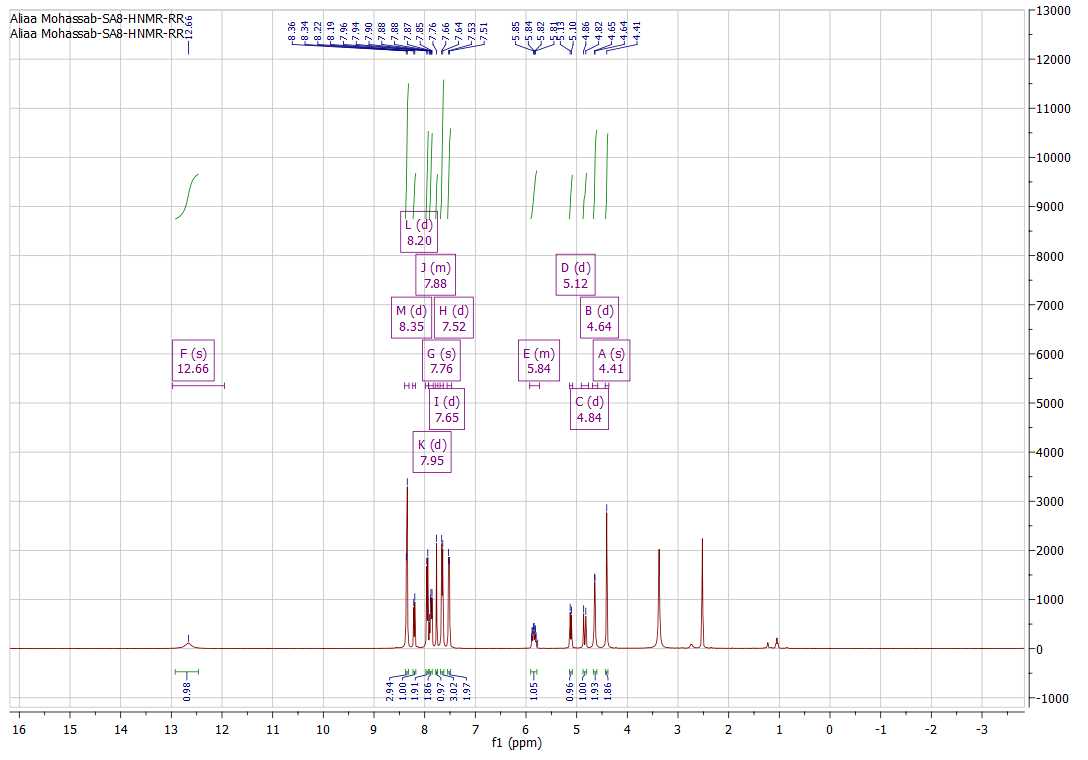** |
| --- |
| **Figure S33: ^1^H NMR spectrum of compound 8j** |
| **^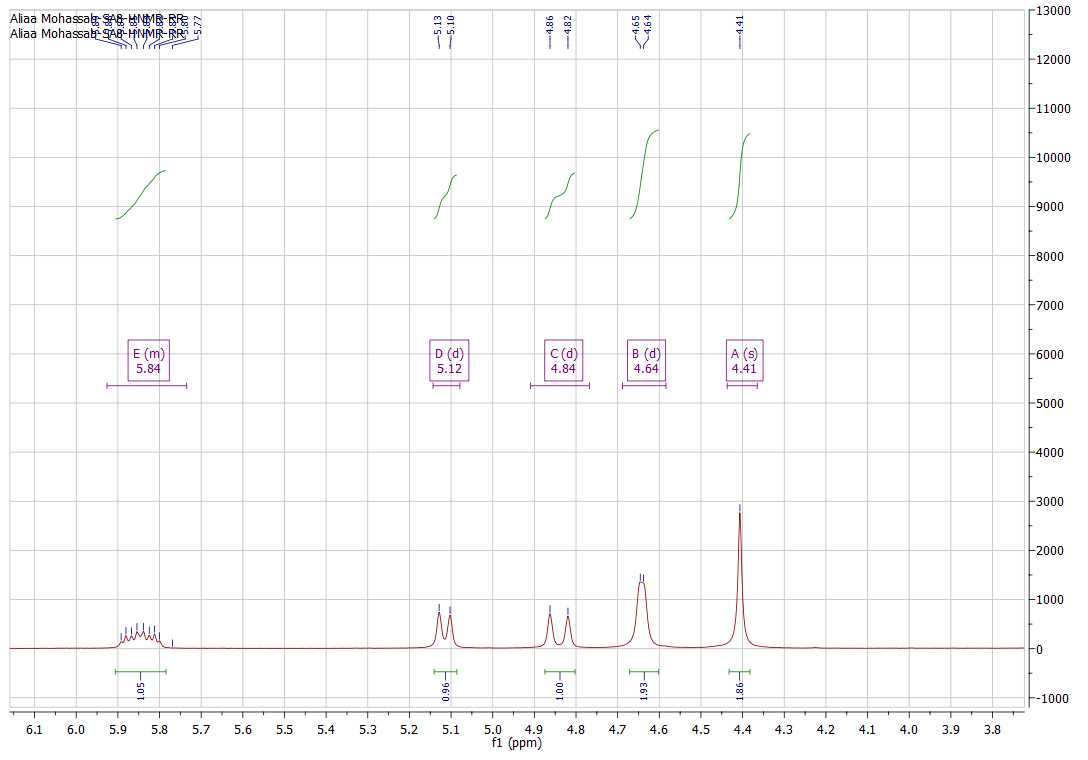^** |
| **Figure S34: Expanded (aliphatic) ^1^H NMR spectrum of compound 8j** |
| **^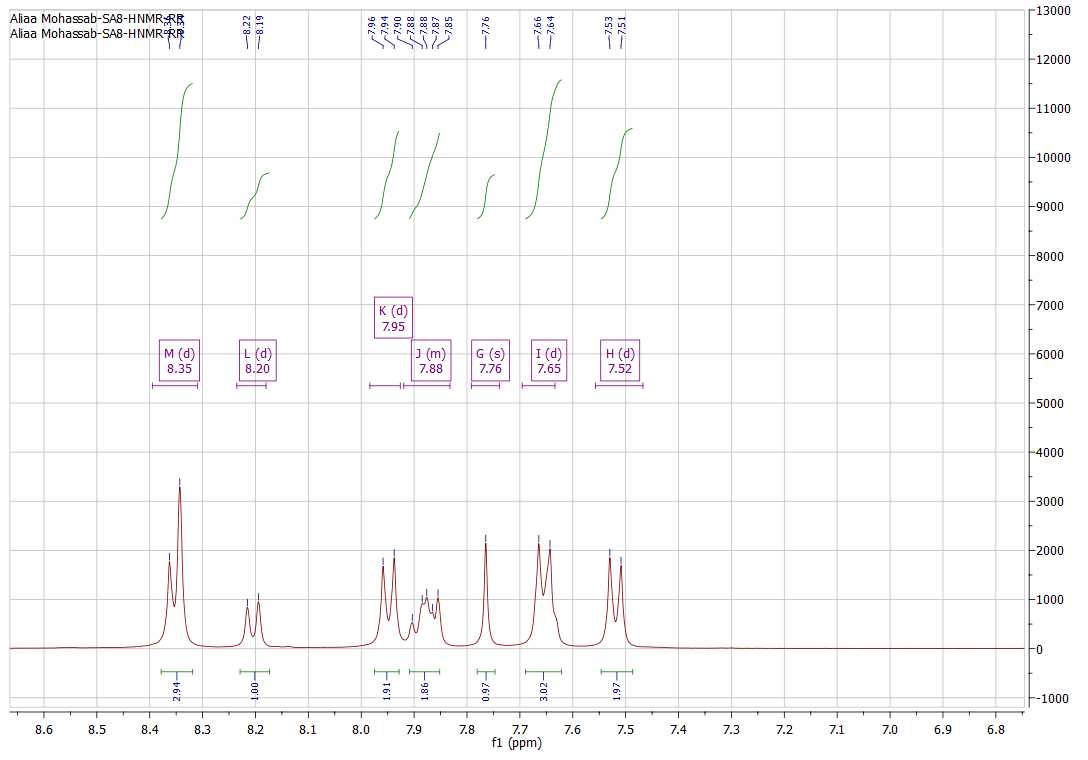^** |
| **Figure S35: Expanded (aromatic) ^1^H NMR spectrum of compound 8j** |
| **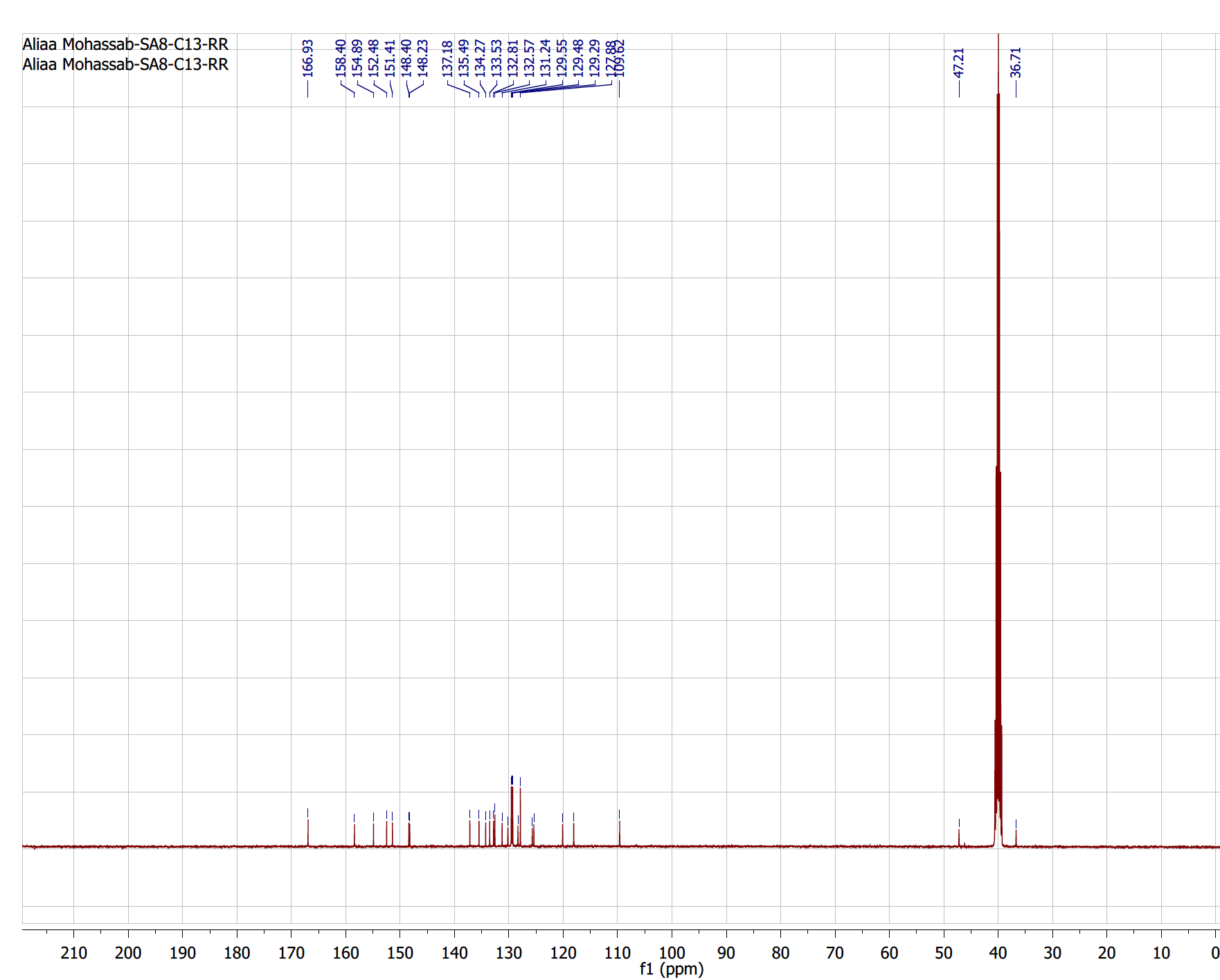** |
| **Figure S36: ^13^C NMR spectrum of compound 8j** |

| **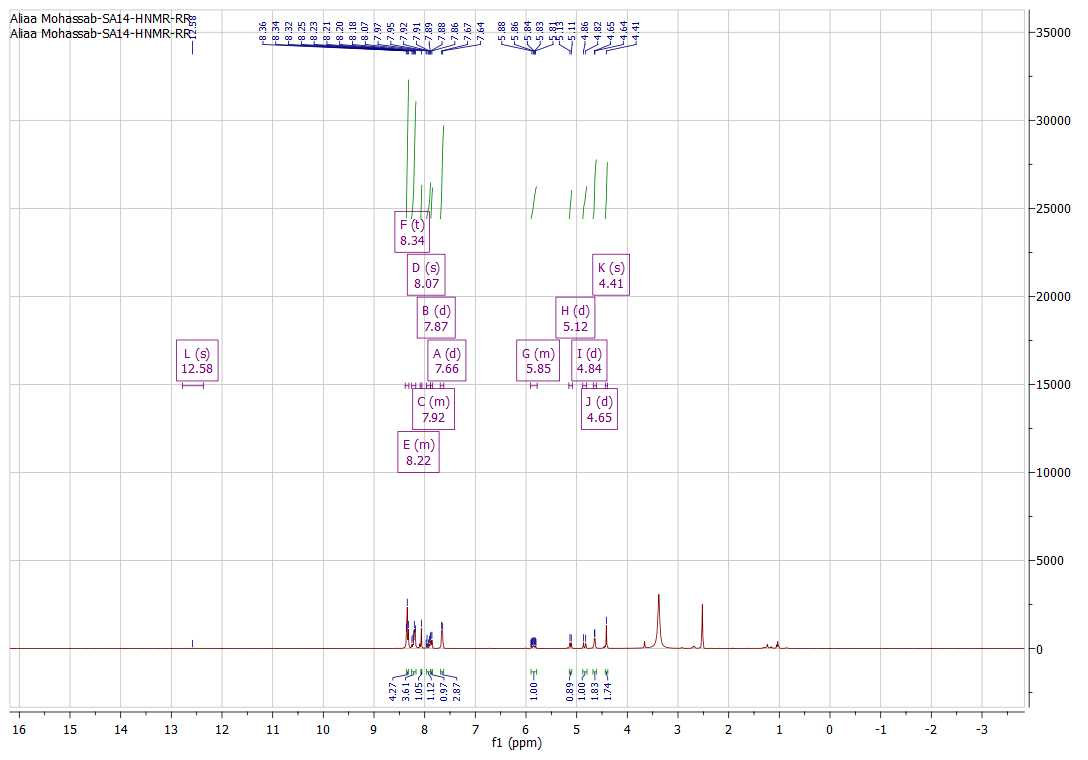** |
| --- |
| **Figure S37: ^1^H NMR spectrum of compound 8k** |
| **^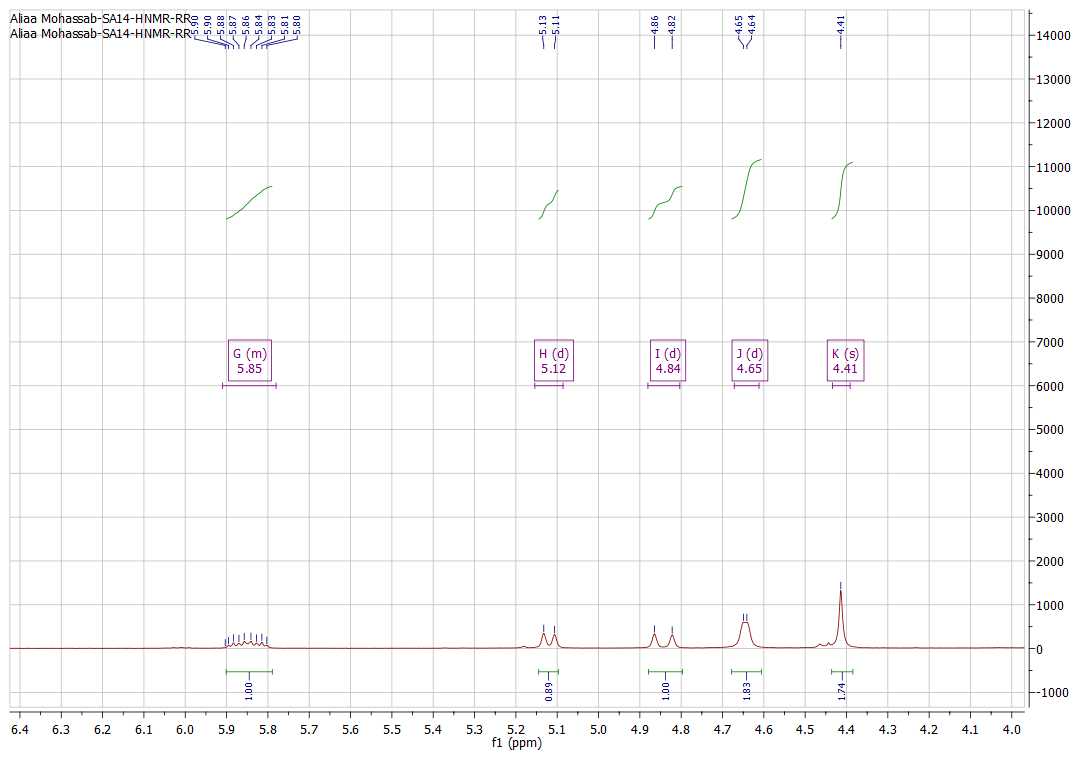^** |
| **Figure S38: Expanded (aliphatic) ^1^H NMR spectrum of compound 8k** |
| **^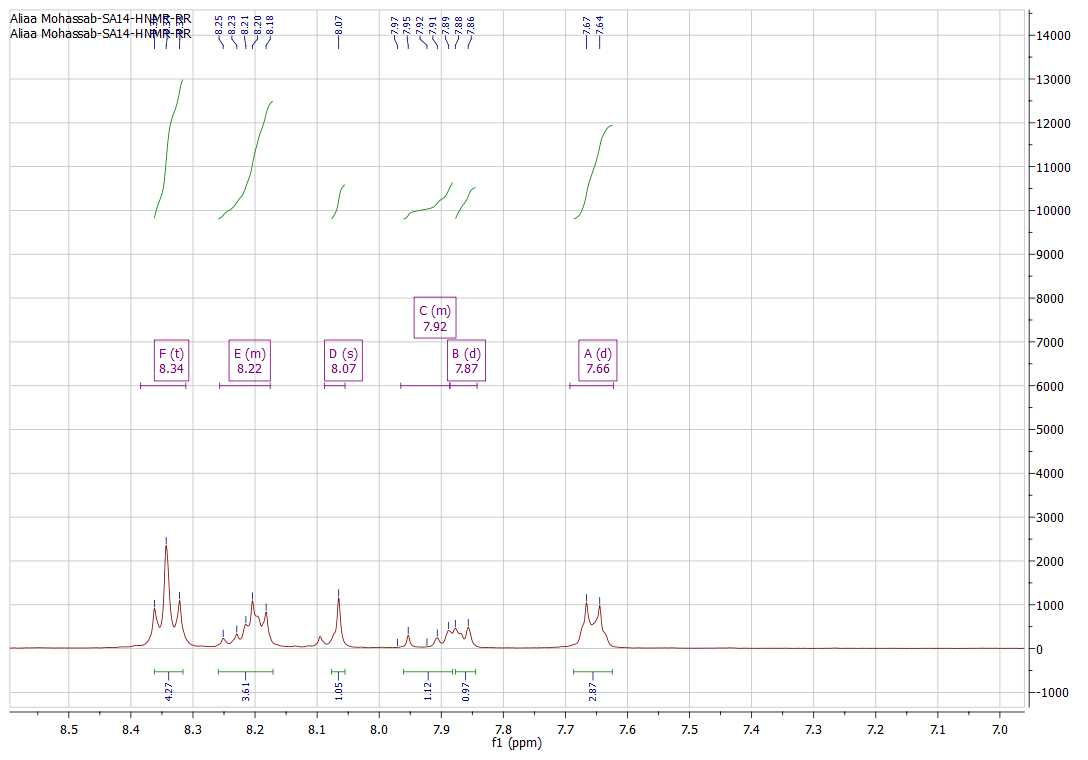^** |
| **Figure S39: Expanded ^1^H NMR spectrum of compound 8k** |
| **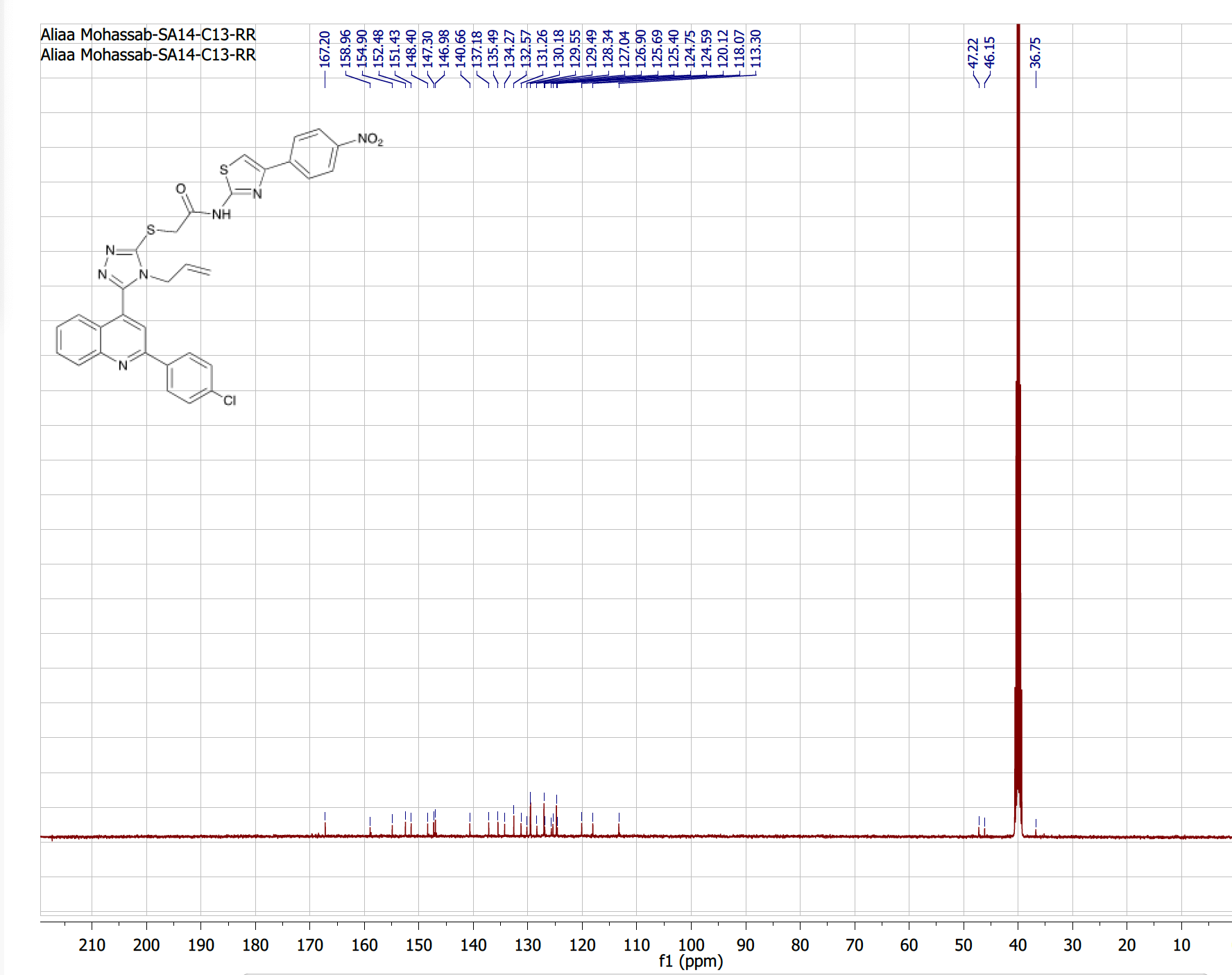** |
| **Figure S40: ^13^C NMR spectrum of compound 8k** |
| **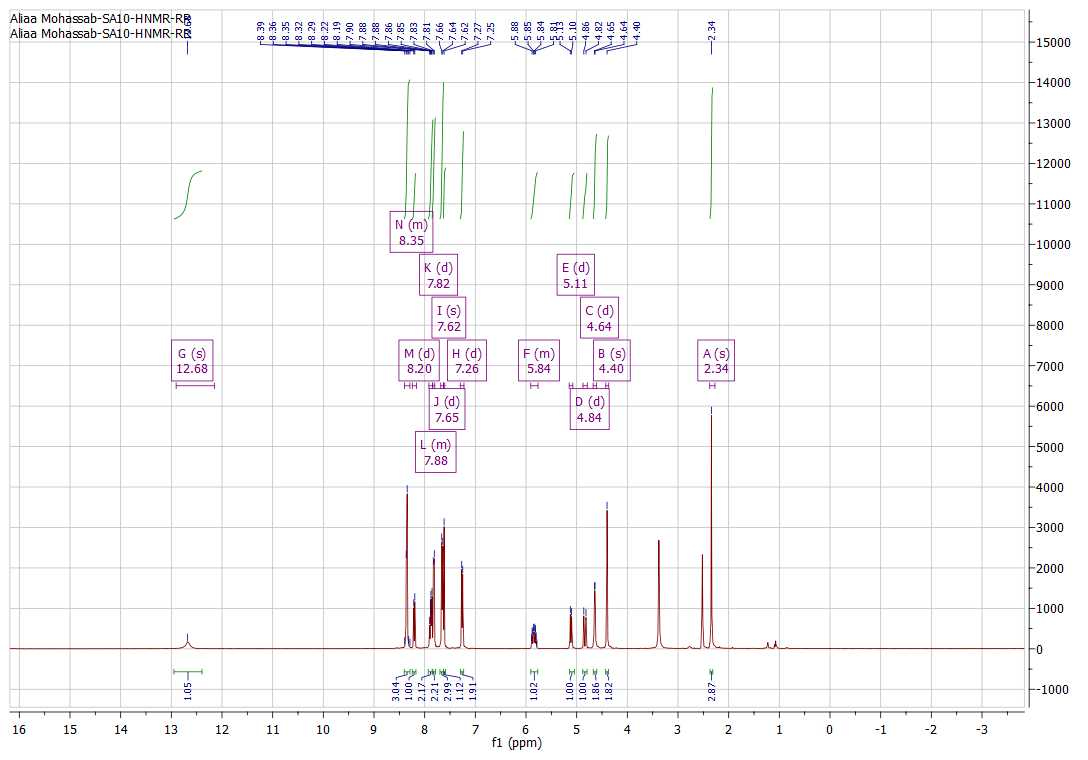** |
| **Figure S41: ^1^H NMR spectrum of compound 8l** |
| **^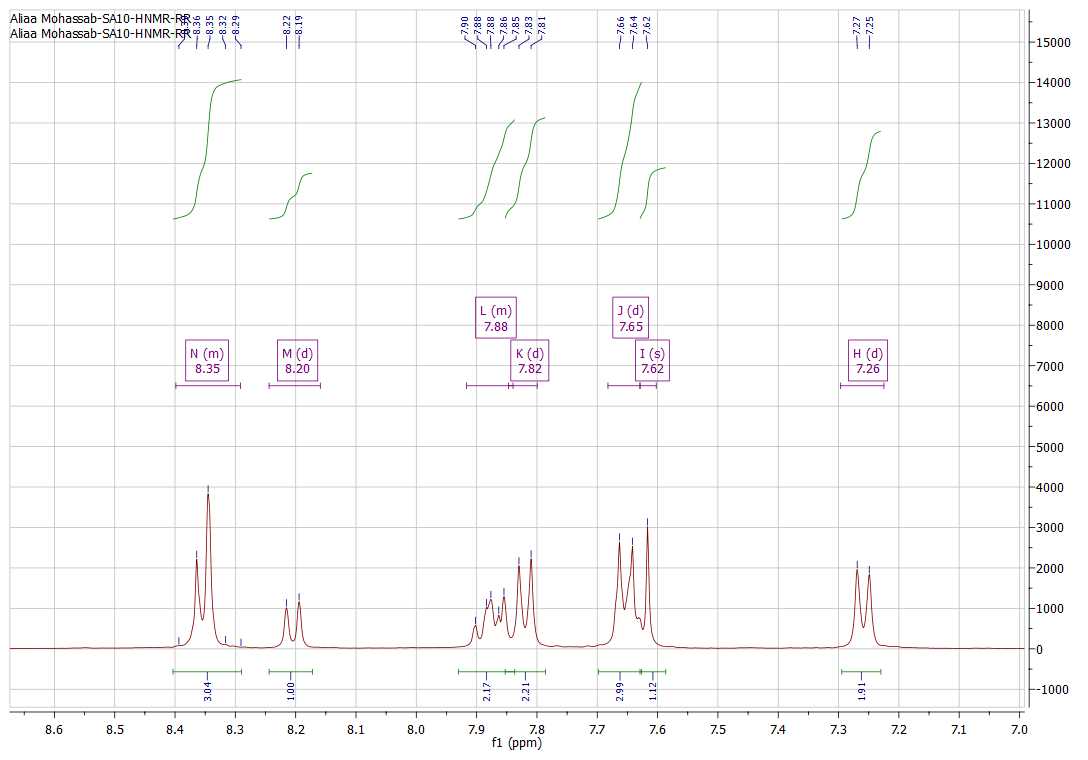^** |
| **Figure S42: Expanded ^1^H NMR spectrum of compound 8l** |
| **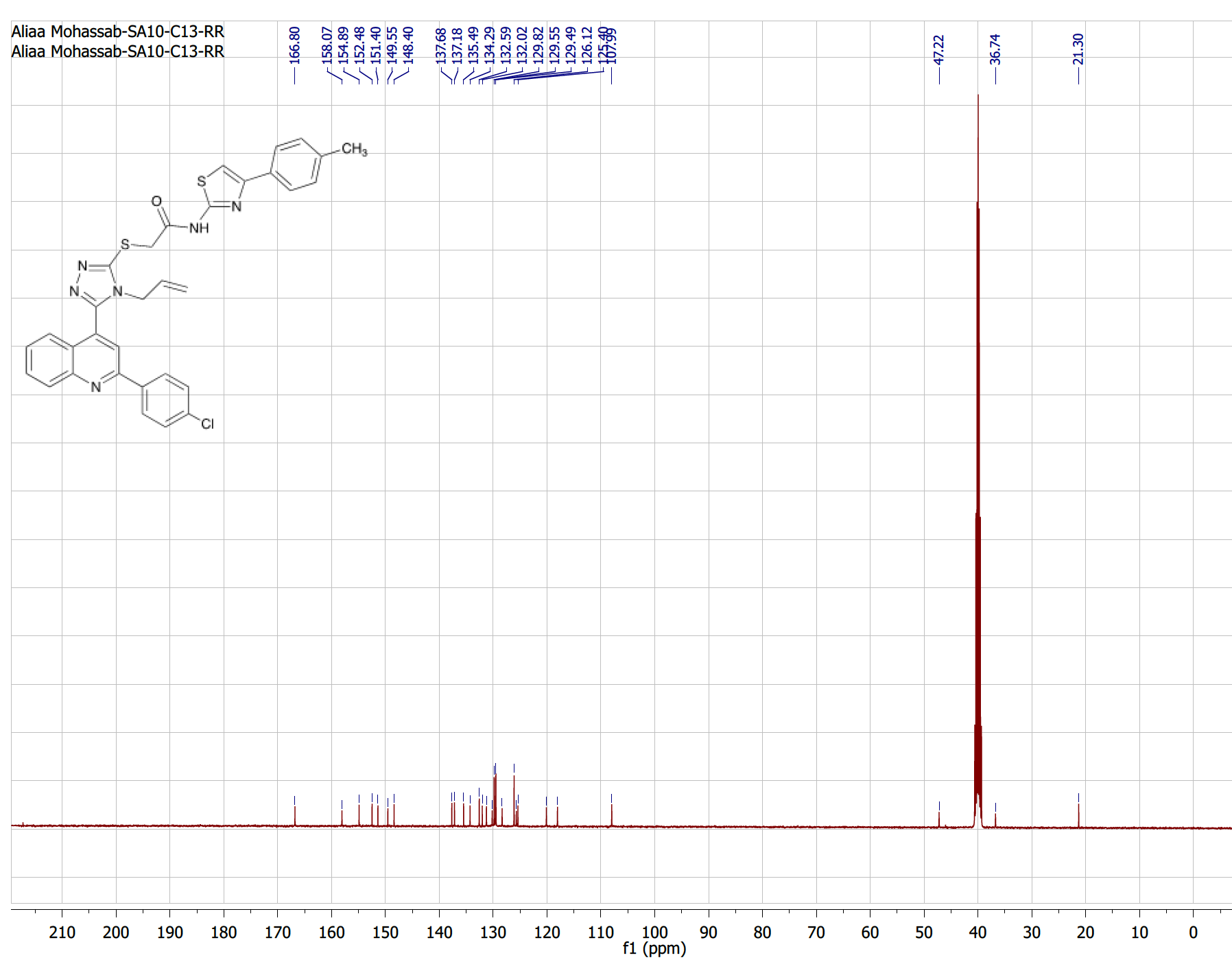** |
| **Figure S43: ^13^C NMR spectrum of compound 8l** |
| **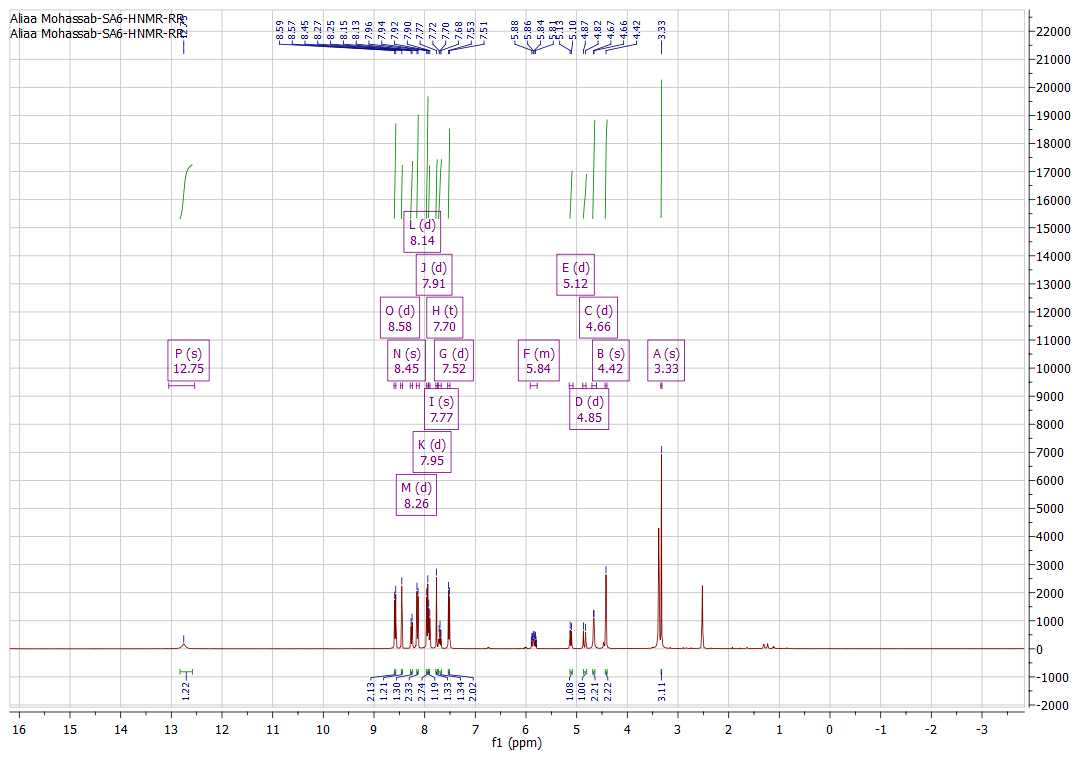** |
| **Figure S44: ^1^H NMR spectrum of compound 8m** |
| 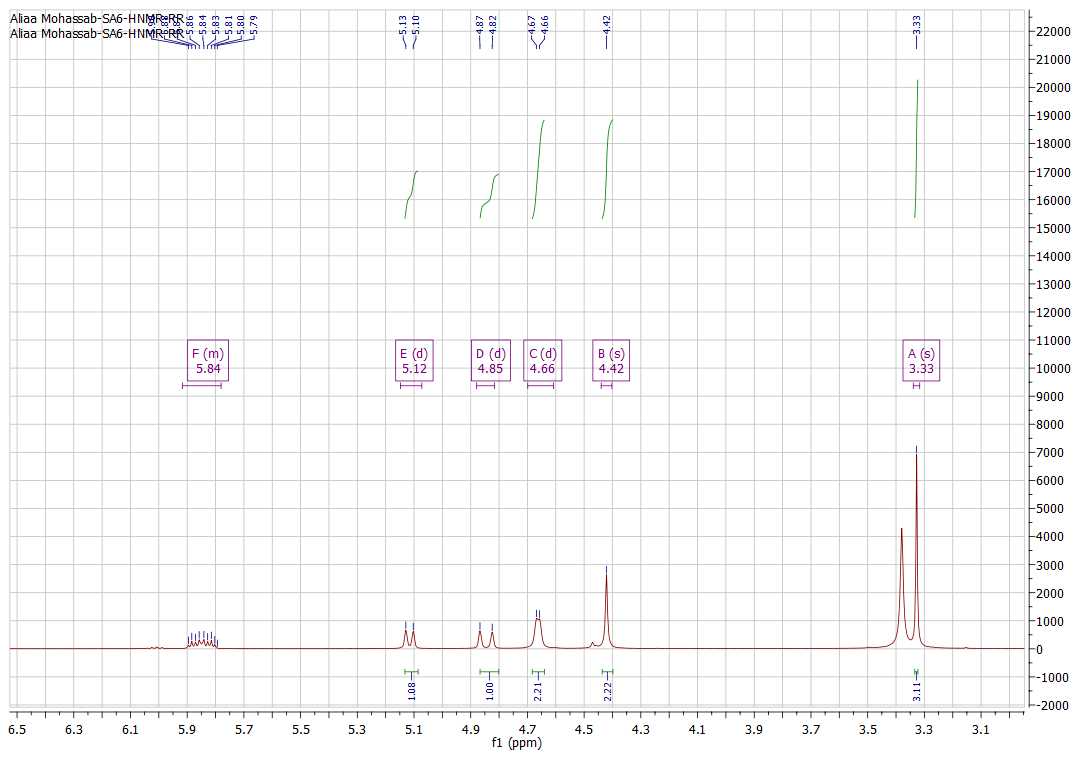 |
| **Figure S45: Expanded ^1^H NMR spectrum of compound 8m** |
| 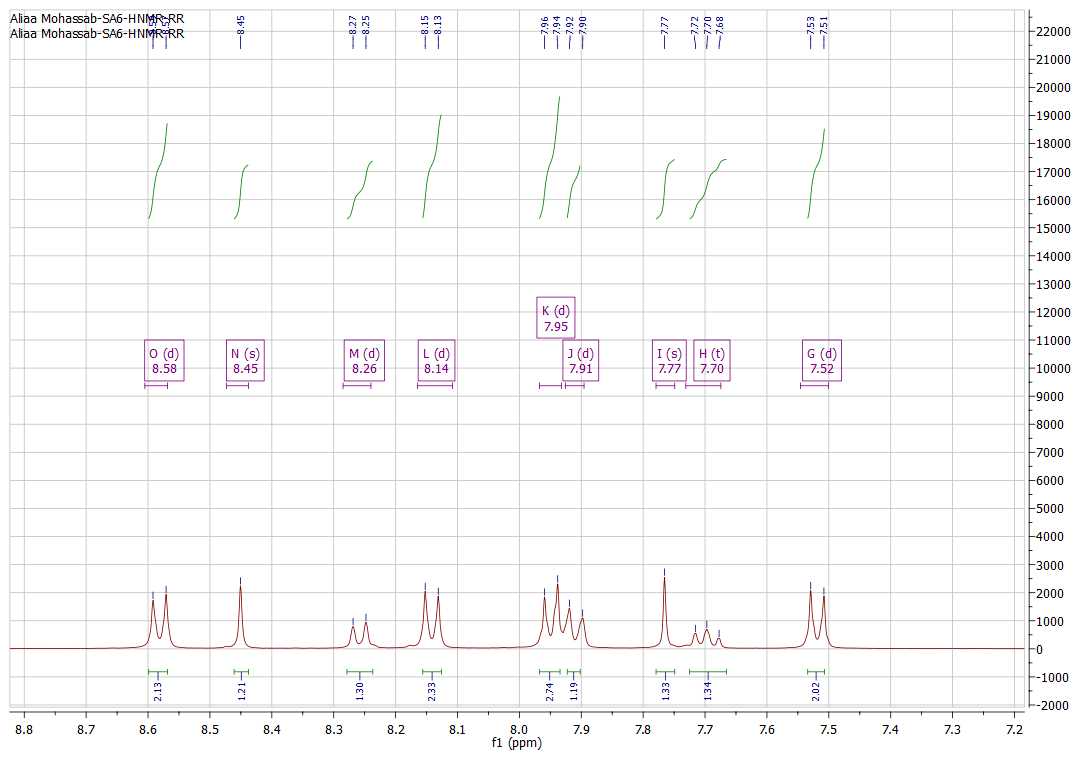 |
| **Figure S46: Expanded ^1^H NMR spectrum of compound 8m** |
| **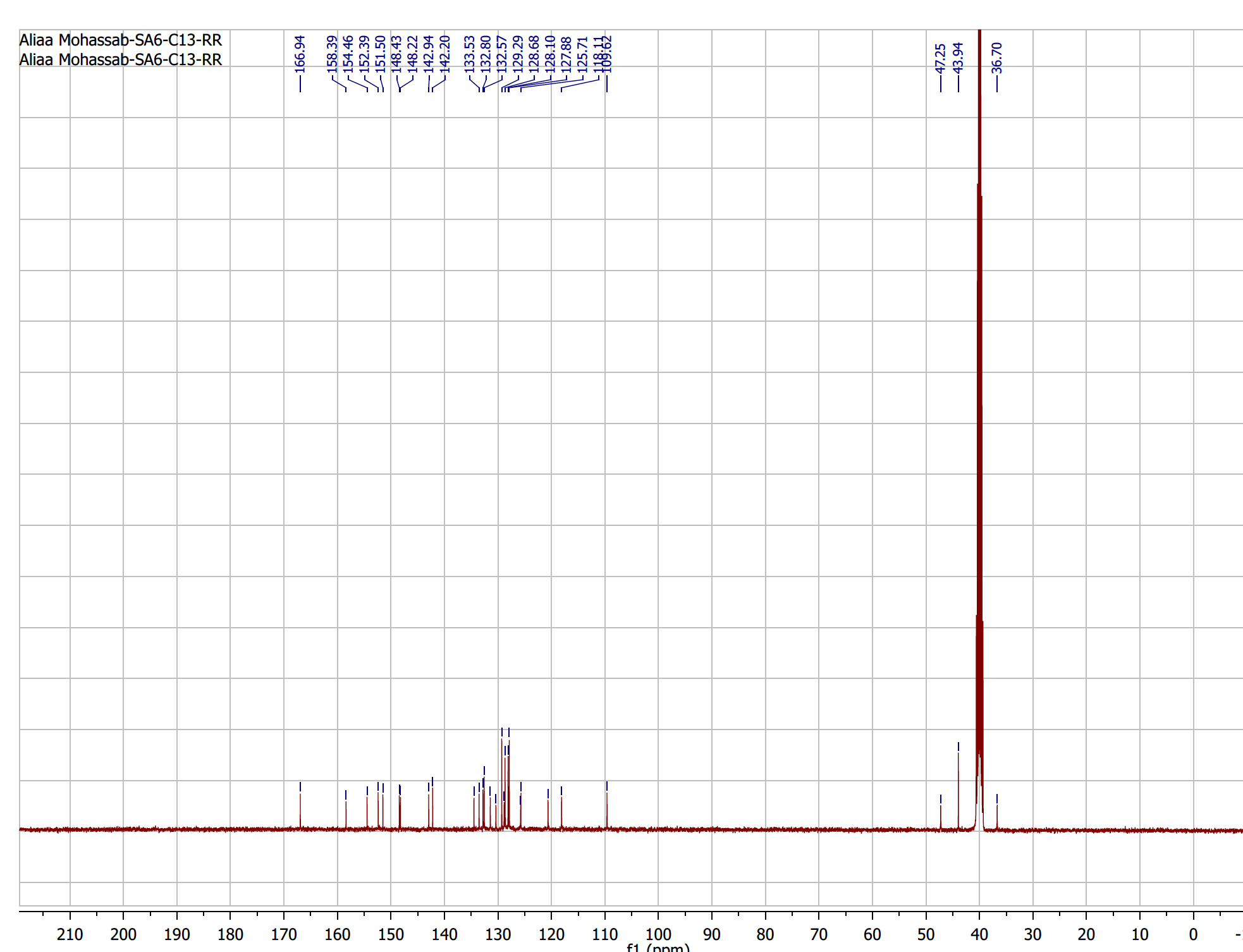** |
| **Figure S47: ^13^C NMR spectrum of compound 8m** |
| **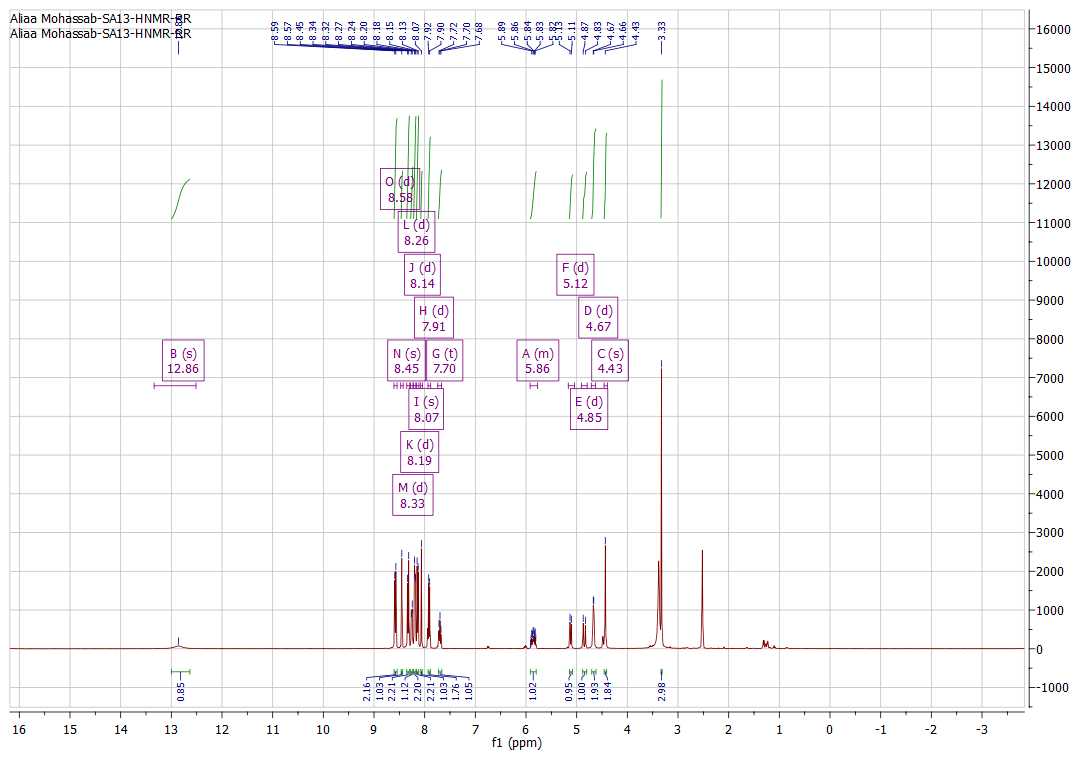** |
| **Figure S48: ^1^H NMR spectrum of compound 8n** |
| **^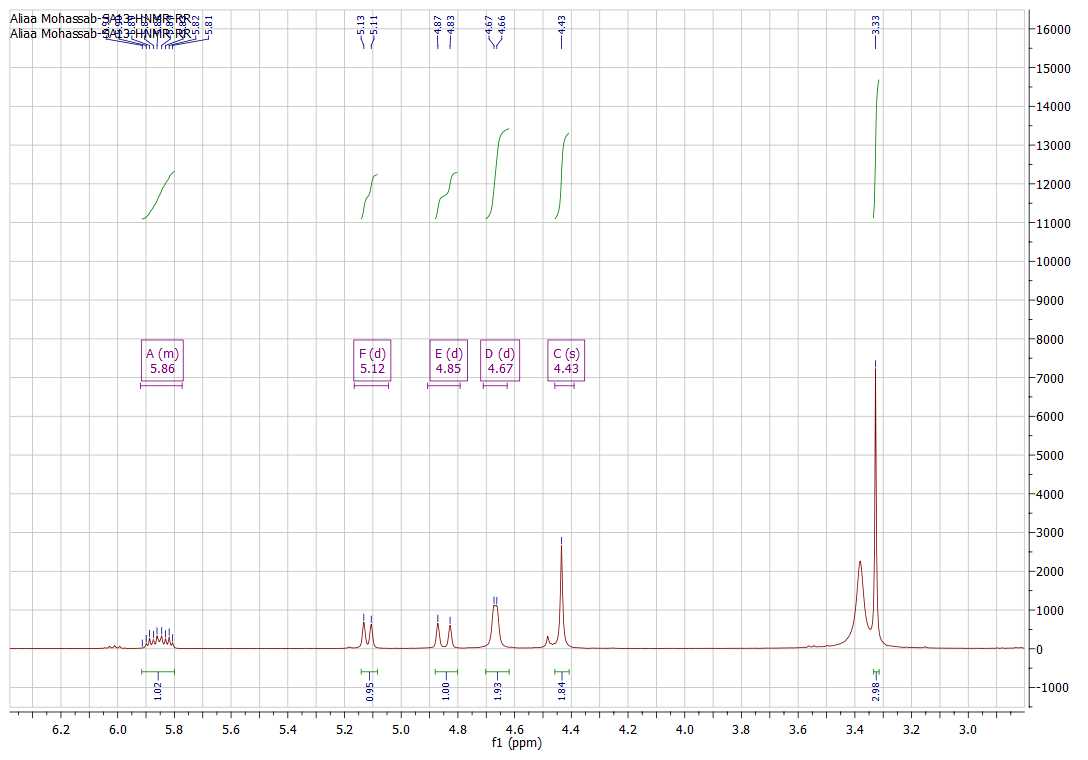^** |
| **Figure S49: Expanded ^1^H NMR spectrum of compound 8n** |
| **^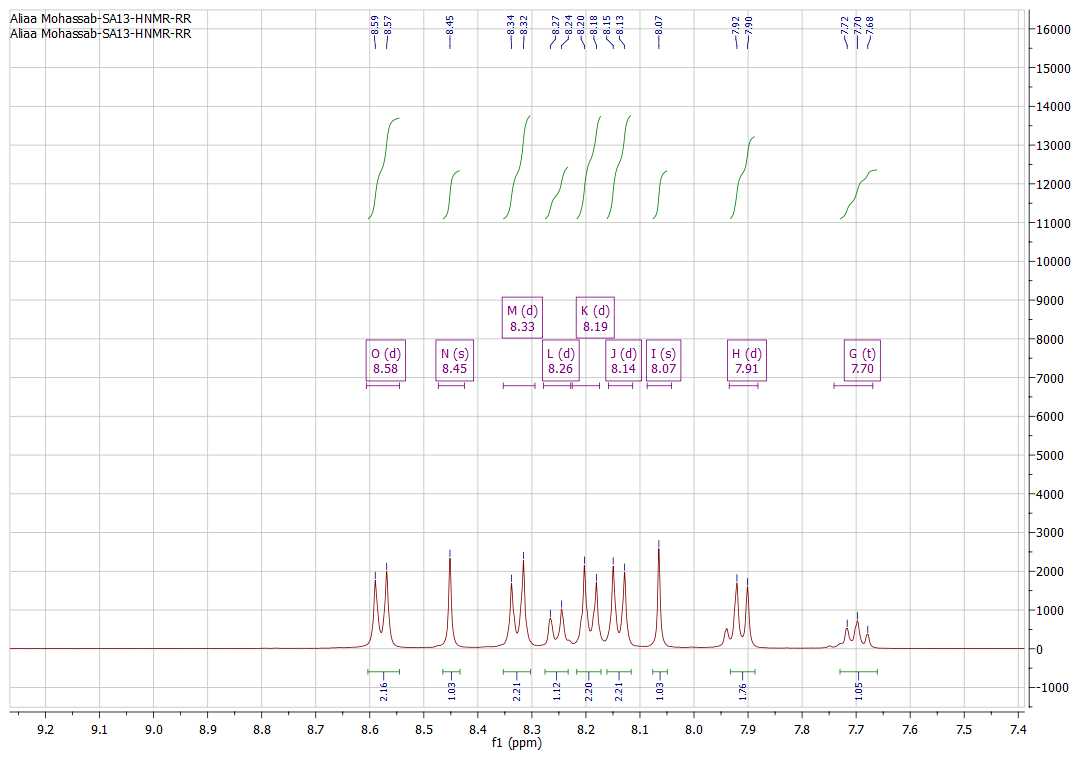^** |
| **Figure S50: Expanded ^1^H NMR spectrum of compound 8n** |
| **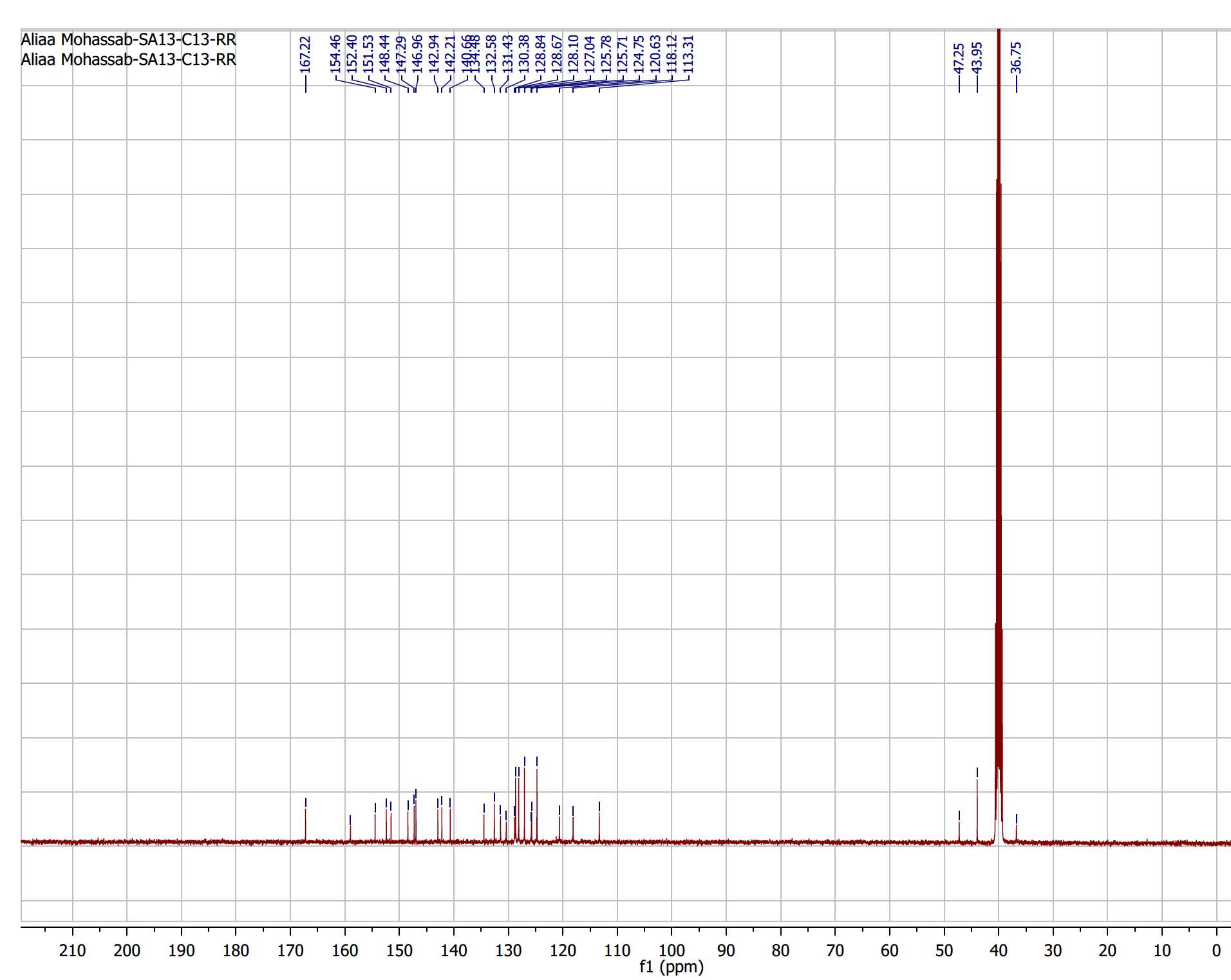** |
| **Figure S51: ^13^C NMR spectrum of compound 8n** |

| **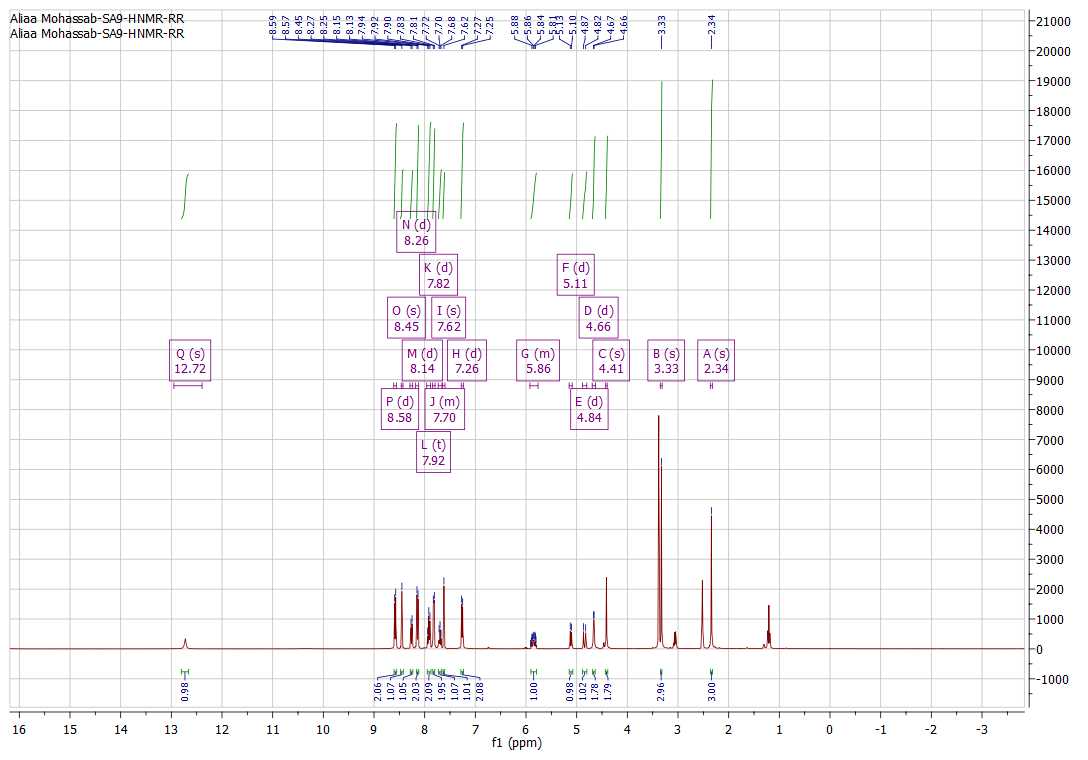** |
| --- |
| **Figure S52: ^1^H NMR spectrum of compound 8o** |
| **^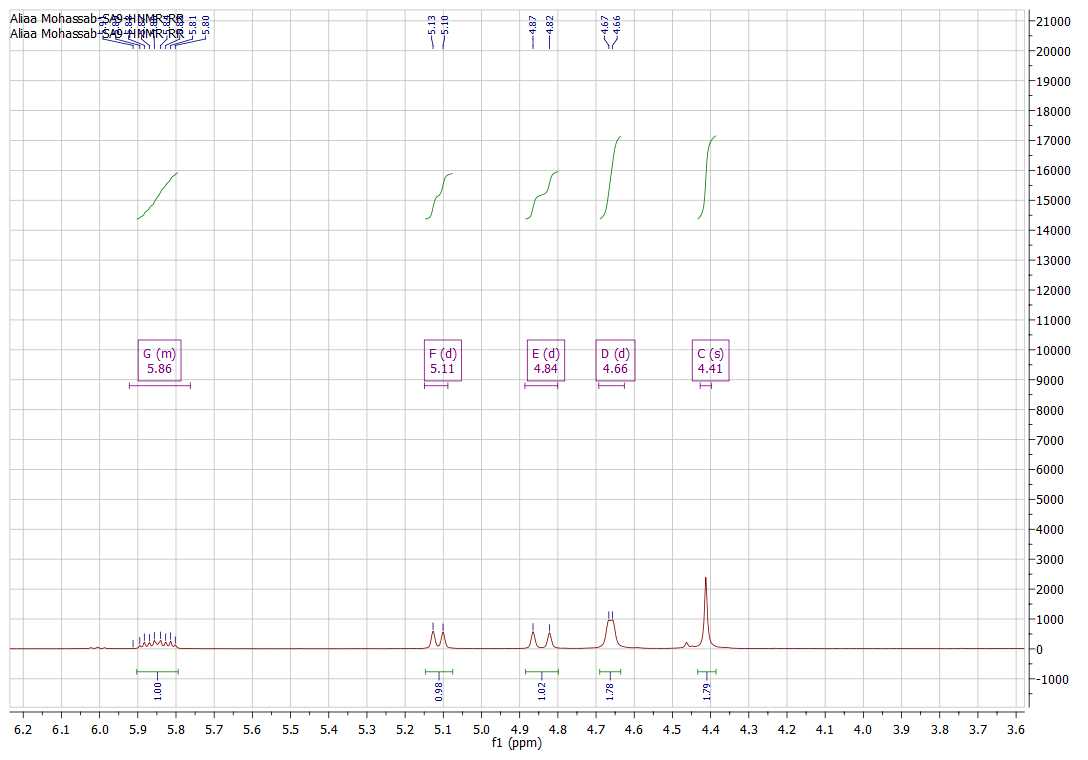^** |
| **Figure S53: Expanded ^1^H NMR spectrum of compound 8o** |
| **^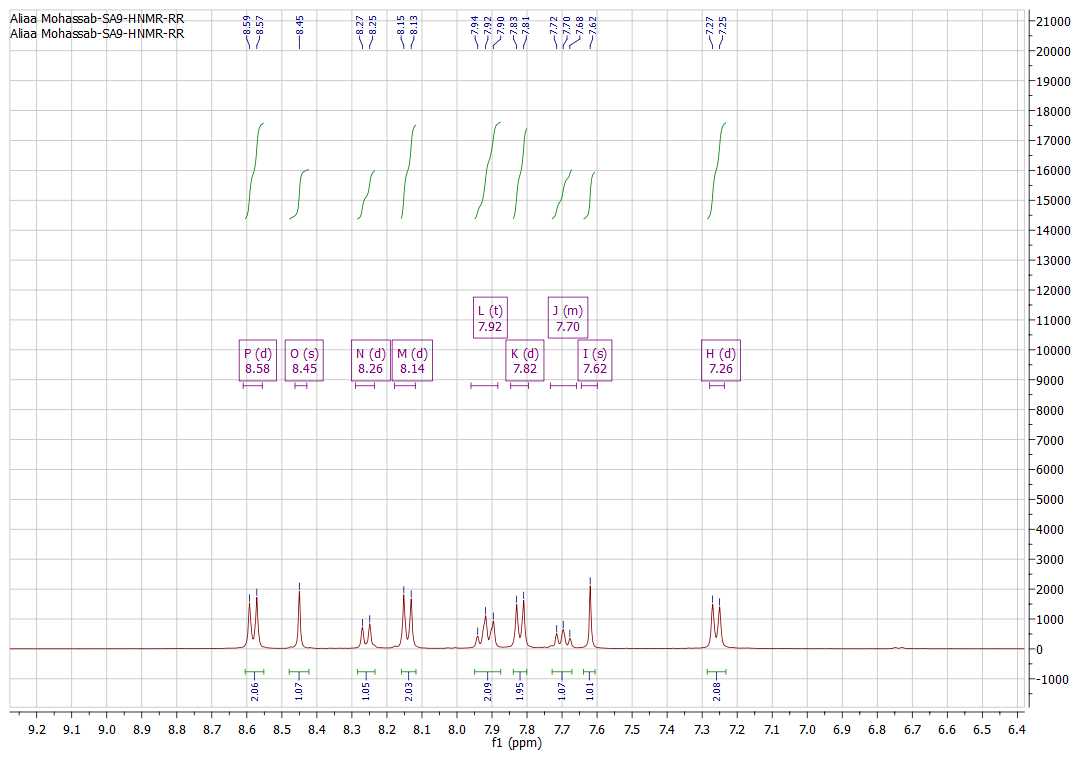^** |
| **Figure S54: Expanded ^1^H NMR spectrum of compound 8o** |
| **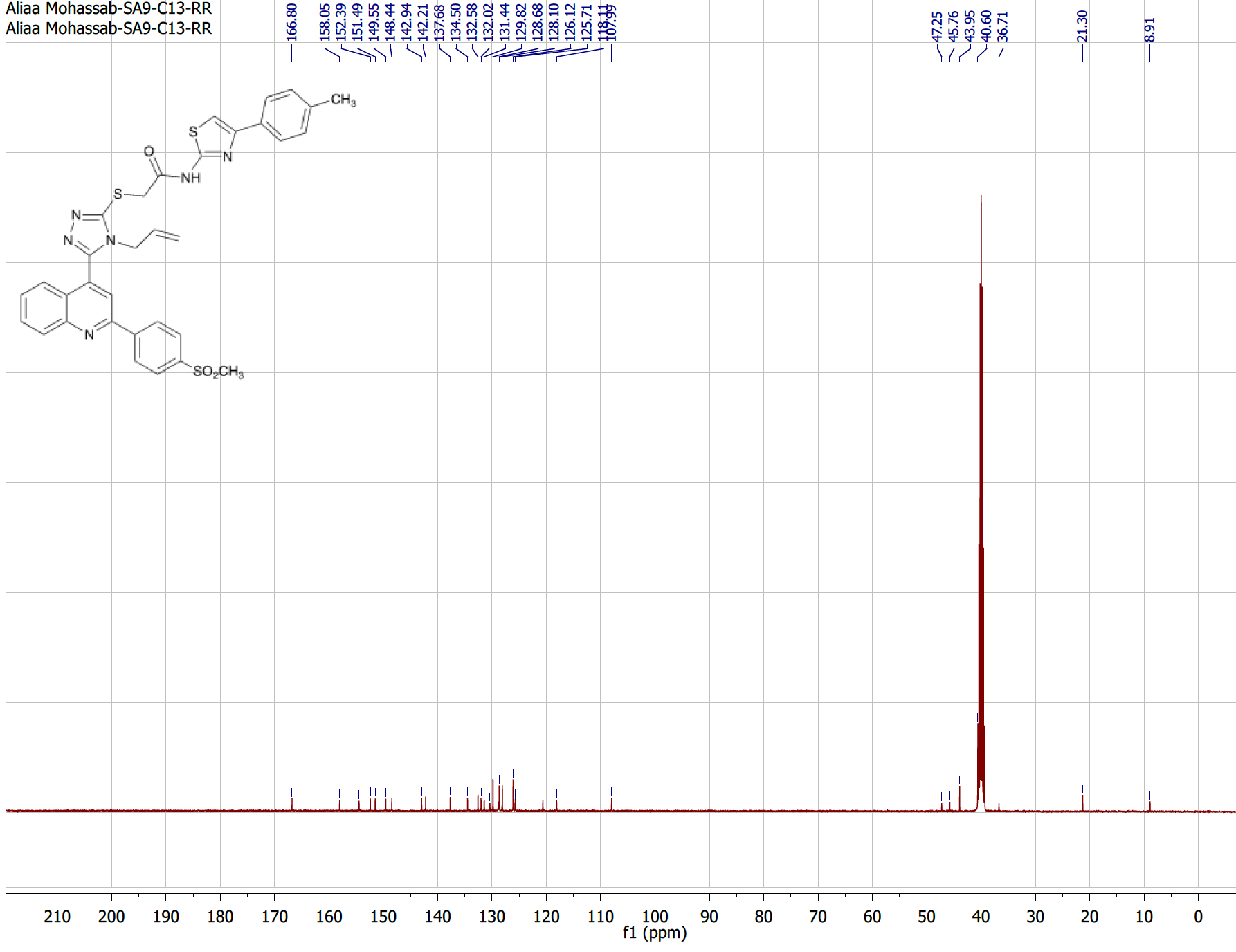** |
| **Figure S55: ^13^C NMR spectrum of compound 8o** |

| **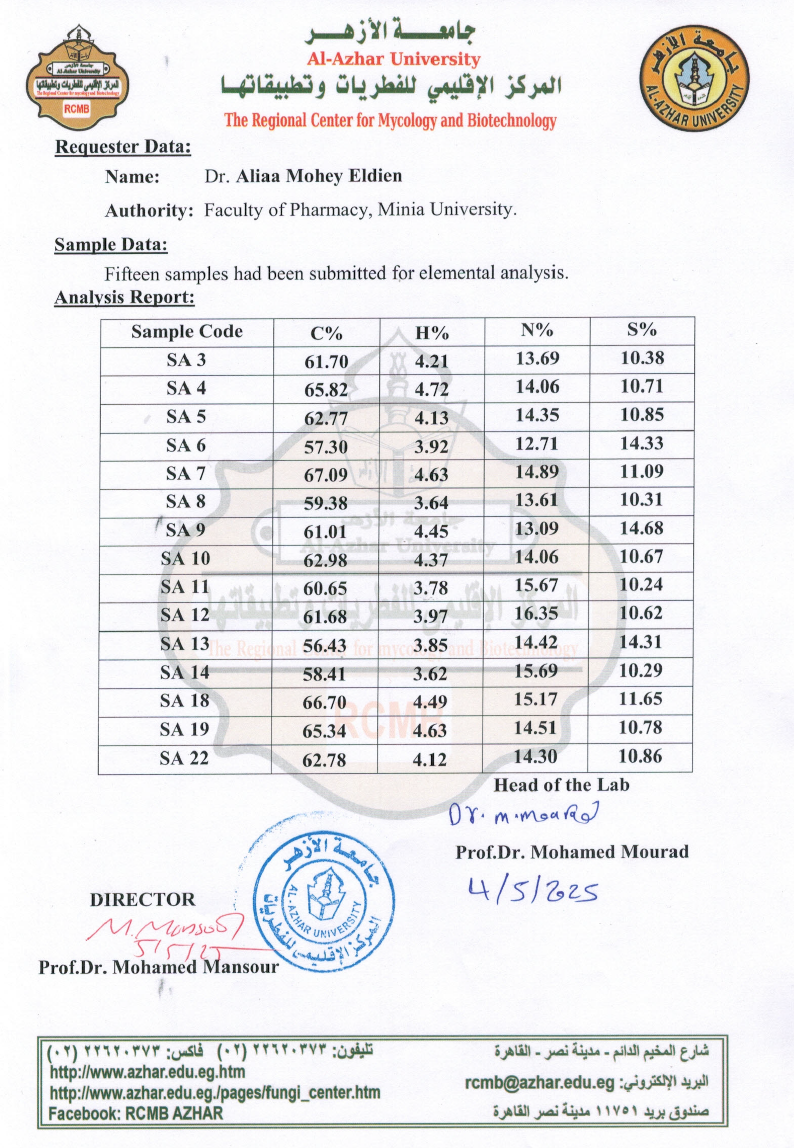** |
| --- |
| **Figure S56: Elemental analyses of compounds 8a-8o** |
